# Translation-dependent mRNA localization to *Caenorhabditis elegans* adherens junctions

**DOI:** 10.1242/dev.200027

**Published:** 2021-12-16

**Authors:** Cristina Tocchini, Michèle Rohner, Laurent Guerard, Poulomi Ray, Stephen E. Von Stetina, Susan E. Mango

**Affiliations:** 1 Biozentrum, University of Basel, 4056 Basel, Switzerland; 2 Cell, Developmental and Regenerative Biology, Icahn School of Medicine at Mount Sinai, New York, NY 10029, USA; 3 MIT Media Lab, Cambridge, MA 02139, USA

**Keywords:** *dlg-1*, Apical junctions, Epithelial morphogenesis, mRNA localization

## Abstract

mRNA localization is an evolutionarily widespread phenomenon that can facilitate subcellular protein targeting. Extensive work has focused on mRNA targeting through ‘zip-codes’ within untranslated regions (UTRs), whereas much less is known about translation-dependent cues. Here, we examine mRNA localization in *Caenorhabditis elegans* embryonic epithelia. From an smFISH-based survey, we identified mRNAs associated with the cell membrane or cortex, and with apical junctions in a stage- and cell type-specific manner. Mutational analyses for one of these transcripts, *dlg-1*/*discs large*, revealed that it relied on a translation-dependent process and did not require its 5′ or 3′ UTRs. We suggest a model in which *dlg-1* transcripts are co-translationally localized with the nascent protein: first the translating complex goes to the cell membrane using sequences located at the C-terminal/3′ end, and then apically using N-terminal/5′ sequences. These studies identify a translation-based process for mRNA localization within developing epithelia and determine the necessary cis-acting sequences for *dlg-1* mRNA targeting.

## Abstract (precis)

**Summary:** An smFISH-based survey identifies a subset of mRNAs encoding junctional components that localize at or in proximity to the adherens junction through a translation-dependent mechanism.

## INTRODUCTION

mRNA localization is an efficient means with which to place the associated translation products in the appropriate subcellular location (Ephrussi et al., 1991; Lecuyer et al., 2007; Takizawa et al., 1997). Large-scale studies in diverse organisms have revealed that many mRNAs are enriched at specific subcellular loci (Jambor et al., 2015; Lecuyer et al., 2007). This mechanism is essential to establish embryonic patterning (Frigerio et al., 1986; Rebagliati et al., 1985), to distribute determinants asymmetrically in precursor cells (Broadus et al., 1998; Li et al., 1997) and to segregate functionally distinct compartments in differentiated and polarized cells like neurons or epithelial cells (Ryder and Lerit, 2018). For example, a global analysis of localized mRNAs in murine intestinal epithelia found that 30% of highly expressed transcripts were polarized, and that their localization coincided with highly abundant regions in ribosomes (Moor et al., 2017). The frequent close apposition of mRNAs and their translated proteins indicates that one function of mRNA localization is to enrich proteins at their final destinations through localized translation (Das et al., 2021; Ryder and Lerit, 2018). However, other functions exist, such as targeted protein degradation (Chouaib et al., 2020), translational repression (Kourtidis et al., 2017) and RNA stabilization or storage (Standart and Weil, 2018).

Cells use a variety of mechanisms to position mRNAs within cells. UTRs often harbor localization elements (‘zip-codes’) that dictate where an mRNA should be delivered (Katz et al., 2012; Kislauskis et al., 1994; Nagaoka et al., 2012). Correct splicing and the presence of exon junction complexes (EJCs) can also play a role in mRNA enrichment to certain subcellular localizations (Hachet and Ephrussi, 2004; Kwon et al., 2021). On the other hand, mRNAs encoding transmembrane or secreted proteins can be localized through a translation-dependent mechanism. For example, the localization factor signal recognition particle binds the nascent signal peptide of endoplasmic reticulum (ER)-bound proteins, arrests cytoplasmic translation and docks at the ER. Translation is resumed after docking, and a transmembrane machinery allows the translocation of the fully synthetized proteins into the ER (Weis et al., 2013). More recently, studies with translation inhibitors puromycin and cycloheximide have implicated nascent peptides for mRNA localization to other membranous organelles or non-P body foci, but the exact mechanisms for delivery are unknown for most genes (Chouaib et al., 2020).

In *C. elegans*, mRNA localization has been studied mainly in the context of post-transcriptional gene silencing in membraneless organelles, specifically germline P granules, somatic P-bodies and stress granules, where transcripts are stabilized (Scheckel et al., 2012), protected from degradation or small RNA-mediated gene silencing (Gallo et al., 2008; Ouyang et al., 2019; Shukla et al., 2020), or repressed translationally (Voronina, 2013). In these instances, mRNAs are commonly post-transcriptionally regulated and localized through their 3′UTRs (Parker et al., 2020; Wright et al., 2011). 3′UTR-dependent mRNA localization also occurs in axons of adult neurons (Yan et al., 2009), where mRNA localization is paired with local translation. However, not all localized mRNAs rely on their 3′UTRs. A recent study on the early *C. elegans* embryo demonstrated the dispensability of 3′UTR to localize at least two mRNAs (*erm-1* and *imb-2*; Parker et al., 2020). However, the mechanisms that localize these mRNAs are currently unknown.

In this study, we focused on mRNA localization during development of *C. elegans* embryonic epithelia. *C. elegans* embryogenesis is highly stereotyped, giving rise to an invariant number and positioning of epithelial cells. Epithelial morphogenesis starts from the embryonic stage of eight endodermal cells (8E stage) (Sulston et al., 1983), when cell junctions, commonly referred to as the *C. elegans* apical junction (*Ce*AJ) (McMahon et al., 2001), begin to form. *Ce*AJs are fully established during the so-called bean and comma stages (names attributed to the early elongation stages based on the shape of the embryo; Sulston et al., 1983). *C. elegans* possesses a single type of apical junction that comprises two adjacent adhesion systems: AS-I and AS-II (Bossinger et al., 2015). AS-I is composed of a cadherin-catenin complex (CCC), constituted by HMR-1/E-cadherin, HMP-1/α-catenin, HMP-2/β-catenin and JAC-1/p120-catenin, which links to intermediate filaments of the cytoskeleton and F-actin (Costa et al., 1998; Pettitt et al., 2003). Additional cytoskeletal organizers (e.g. SMA-1) contribute to the correct architecture of AS-I (McKeown et al., 1998). In AS-II, a DLG-1/Discs Large and AJM-1 complex (DAC) provides a link between the proposed adhesion molecule of the AS-II, called SAX-7/L1CAM (Chen and Zhou, 2010), and cytoskeletal-associated components SMA-1/βH-spectrin, ERM-1/ezrin/radixin/moesin, and actin filaments (Bernadskaya et al., 2011; Gobel et al., 2004; McKeown et al., 1998; Van Furden et al., 2004). A series of evolutionarily conserved ancillary proteins (actin filaments, claudins, spectrins and PAR proteins, etc.) help form and maintain the *Ce*AJs (Armenti and Nance, 2012).

To investigate the existence of mRNA localization during embryonic development, we conducted a single molecule fluorescence *in situ* hybridization (smFISH)-based survey on the *C. elegans* embryo and tested the localization of mRNAs coding for factors belonging to AS-I and AS-II, as well as for proteins involved in *Ce*AJ formation and maintenance. We identified transcripts enriched at the *Ce*AJ in a stage- and cell type-specific manner. Genetic and imaging analyses of transgenic lines for one of the identified localized mRNAs, *dlg-1/discs large*, mapped domains required for localization. Our data demonstrate that the *dlg-1* UTRs are dispensable, whereas translation in *cis* is required for localization, therefore providing an example of a translation-dependent mechanism for mRNA delivery in *C. elegans*.

## RESULTS

### mRNAs coding for the main components of the cell adhesion system II are enriched at the *Ce*AJ

**Fig. 1.**
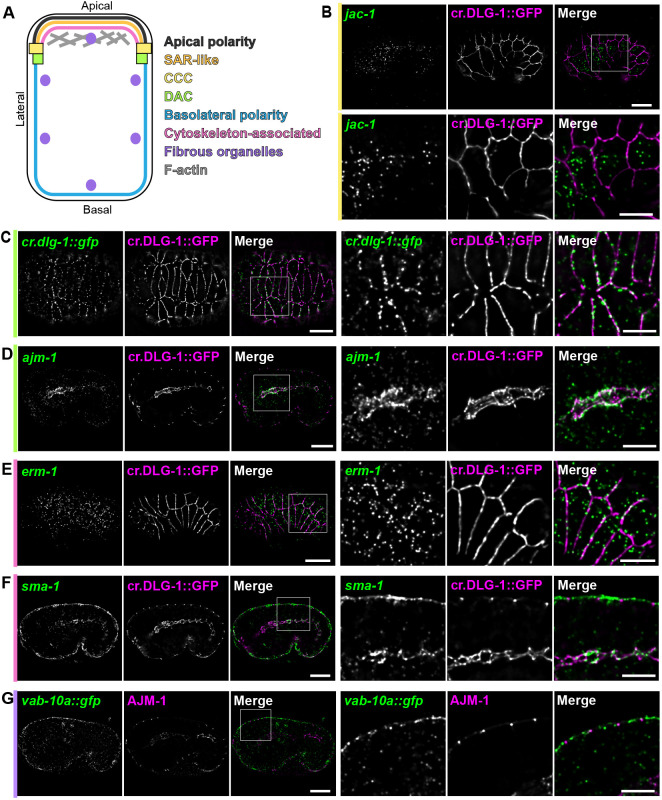
**Five mRNAs encoding DAC components, basolateral polarity factors and a fibrous organelle-bound protein are enriched at the cell membrane.** (A) Simplified color-coded schematics of a *C. elegans* epithelial cell, highlighting the classes of factors involved in apicobasal polarity and maintenance of epithelial morphology. Black line indicates apical polarity factors. Orange line indicates subapical region-like (SAR-like). Pink line indicates cytoskeletal-associated components. Yellow rectangles indicate cadherin-catenin complex (CCC). Green squares indicate the DLG-1/AJM-1 complex (DAC). Blue line indicates basolateral polarity factors. Purple circles indicate fibrous organelles. Gray lines indicate F-actin filaments (AF). (B) Fluorescent micrographs of a *C. elegans* embryo at the comma stage (upper panels) and zoom-ins (lower panels) showing smFISH signal of an unlocalized mRNAs (*jac-1*, green), fluorescent signal of the endogenous CRISPR-engineered GFP-tagged DLG-1 protein (cr.DLG-1::GFP, magenta) and merges. To the left of the images, bars are color-coded as in A to indicate the sub-class of the factor the mRNA encodes. (C-G) Fluorescent micrographs of entire *C. elegans* embryos (left panels) and zoom-ins (right panels) showing smFISH signal of localized mRNAs [*dlg-1::gfp* in epidermal cells of a bean stage (C), *ajm-1* in pharyngeal cells of a late comma stage (D), *erm-1* in epidermal cells of a bean stage (E), *sma-1* in epidermal and pharyngeal cells of a late comma stage (F) and *vab-10a::gfp* in epidermal cells of a comma stage (G); green], fluorescent signal of *Ce*AJ markers (cr.DLG-1::GFP or endogenous AJM-1, magenta) and merges. Specific embryonic stages were selected for each transcript based on the highest degree of mRNA localization they exhibit. To the left of each image, bars are color-coded as in A to indicate the sub-class of factors the mRNAs encode. White squares indicate the region of the embryo shown in the zoom-ins. Scale bars: 10 µm (entire embryos); 5 µm (zoom-ins).

We began our analysis of mRNA localization by surveying 25 transcripts that code for the major factors involved in cell polarity and *Ce*AJ formation, as well as some cytoskeletal components (Fig. 1A and Table S1) in epithelial cells during morphogenesis. The protein products of the tested mRNAs are localized differentially along the cell membrane/cortex and cytoplasm (Fig. 1A and Table S1). We identified epithelial cells and the *Ce*AJ using a CRISPR-engineered DLG-1::GFP fusion (Heppert et al., 2018). Our survey revealed mRNAs with varying degrees of localization within epithelia, which we divided into three classes: *Ce*AJ/membrane localized, perinuclearly localized and unlocalized (Fig. 1B-G; Figs S1 and S2 and Table S1). Five of these transcripts were enriched at specific loci at or near the cell membrane: laterally and at the *Ce*AJ for *dlg-1* (Fig. 1C for endogenous/GFP CRISPR-tagged *dlg-1::gfp* mRNA and Fig. S1A for endogenous/non-tagged *dlg-1* mRNA), solely at the *Ce*AJ for *ajm-1* and *erm-1* (Fig. 1D,E), apically and at the *Ce*AJ for *sma-1* (Fig. 1F) and apically for *vab-10a* (Fig. 1G). The degree of enrichment varied for these transcripts with some dramatically enriched (*ajm-1* and *dlg-1*) and others only mildly enriched (*erm-1*). Interestingly, all these transcripts apart from *vab-10a* encode the main cytoplasmic components of the AS-II.

Beyond AS-II-coding and *vab-10a* transcripts belonging to the *Ce*AJ/membrane localized mRNA class, our survey detected four mRNAs (*hmr-1*, *sax-7*, *eat-20* and *let-805*) that showed a few instances of perinuclear localization (Fig. S1B). HMR-1/E-cadherin and SAX-7/L1CAM constitute the transmembrane components (putative for SAX-7) of the CCC and the DAC, respectively (Chen and Zhou, 2010; Costa et al., 1998). EAT-20, a Crumbs-like factor involved in apicobasal polarity, and LET-805/fibronectin 1 are also transmembrane proteins (Armenti and Nance, 2012; Shibata et al., 2000). Bioinformatic analyses of their sequences confirmed the presence of signal peptides in all the four proteins (see Materials and Methods). Therefore, the perinuclear localization of their transcripts likely reflects classical ER-associated translation (Hermesh and Jansen, 2013). The rest of our tested mRNAs did not possess any evident subcellular localization at any of the analyzed embryonic stages/tissues and were not further investigated (Fig. S2 and Table S1). Taken together, our smFISH survey revealed nine localized mRNAs, five at the cell membrane and four perinuclear. These data indicate that mRNA membrane localization is a feature of the AS-II cell-adhesion system, except for the putative transmembrane protein-coding *sax-7* and actin mRNAs.

### *dlg-1* and *ajm-1* mRNA enrichment at the apical junction varies in a stage- and cell type-specific manner

**Fig. 2.**
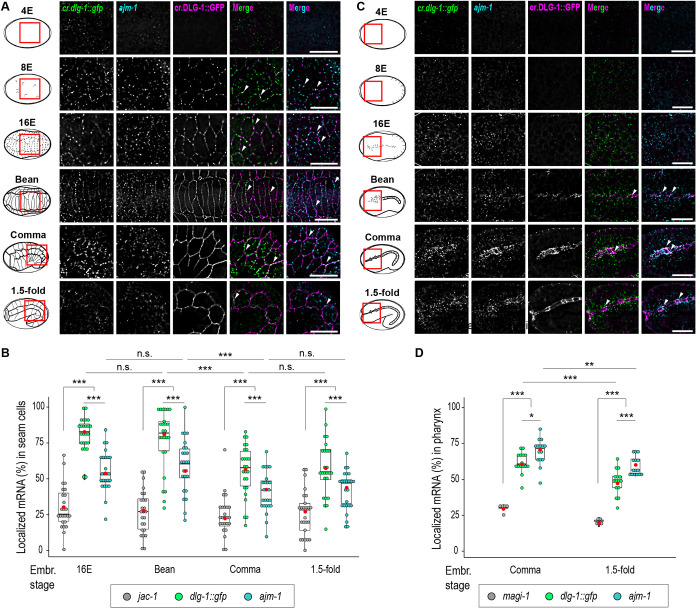
***dlg-1* and *ajm-1* mRNA localization changes dynamically during epithelial morphogenesis.** (A) Left: names and schematics of the analyzed embryonic stages (4E, no junctions; 8E, nascent junctions; 16E, junction maturation; bean, junction formation; comma and 1.5-fold, established junctions). Red squares indicate the region of the embryo shown on the right. Right panels show fluorescent micrographs of epidermal and seam cells of *C. elegans* embryos showing smFISH signal of two localized mRNAs, *dlg-1* (*cr.dlg-1::gfp*, green) and *ajm-1* (cyan), fluorescent signal of the CRISPR-engineered GFP-tagged DLG-1 protein (cr.DLG-1::GFP, magenta), and merges. Scale bars: 5 µm. (B) Box and whisker plot with each dot representing the percentage of laterally localized versus total cellular *jac-1* (unlocalized control, gray), *dlg-1::gfp* (green) and *ajm-1* (cyan) mRNAs (*y*-axis) in each seam cell analyzed at the stated embryonic stages [*x*-axis: 16E (*n*=25), bean (*n*=25), comma (*n*=25) and 1.5-fold (*n*=25)]. Data are derived from five different embryos for each stage. Significance of statistical analyses (*t*-test, two tailed): n.s., not significant; ***P<0.001. (C) As in A but for pharyngeal cells. (D) Box and whisker plot. Each dot represents the percentage of apically localized versus total cellular *magi-1* (unlocalized control, gray), *dlg-1::gfp* (green) and *ajm-1* (cyan) mRNAs (*y*-axis) in each pharynx analyzed at the stated embryonic stages [*x*-axis: comma (*n*=5 for *magi-1* and *n*=15 for *dlg-1::gfp* and *ajm-1*) and 1.5-fold (*n*=5 for *magi-1* and *n*=15 for *dlg-1::gfp* and *ajm-1*]. Significance of statistical analyses (*t*-test, two tails): *P<0.05; **P<0.01; ***P<0.001. In the box and whisker plots, a thick black line represents the median, the two hinges represent first and third quartiles, the two whiskers define the upper and lower limits, and dots represent individual results. Red dots represent the mean.

Close examination of the smFISH data showed that mRNA localization varied in a stage- and cell type-specific manner, including transcripts encoding components of the same complex. Specifically, DLG-1 and AJM-1 form a complex (Bossinger et al., 2001), yet differed in the spatiotemporal localization of their mRNAs during epithelial morphogenesis (Fig. 2A,B). *dlg-1* and *ajm-1* start to be expressed at the 4E embryonic stage (Von Stetina et al., 2017). Although epidermal *Ce*AJs (e*Ce*AJ) do not yet exist at the 4E stage (Fig. 2A, upper-most panels), we detected some *dlg-1* mRNA localized near the cell membrane, marked by the basolateral factor LET-413 (Fig. S3A). During e*Ce*AJ maturation and formation (8E, 16E and bean stages), *dlg-1* and *ajm-1* mRNAs showed a peak in enrichment at or next to the membrane (Fig. 2A,B; Table S2), although *dlg-1* mRNA was more enriched than *ajm-1* [81 versus 55% at the 16E stage (negative control, *jac-1*: 30%) and 77 versus 57% at the bean stage (negative control, *jac-1*: 26%)]. When e*Ce*AJs were fully established (comma and 1.5-fold stages), forming the typical continuous and circumferential belt-like structure at the apical side of the cell membrane, both *dlg-1* and *ajm-1* showed a decrease in enrichment, although *dlg-1* was consistently more enriched than *ajm-1* [54 versus 41% at the comma stage (negative control, *jac-1*: 24%), and 58 versus 42% at the 1.5-fold stage (negative control, *jac-1*: 26%); Fig. 2A,B; Table S2].

Analyses of transverse sections of lateral membranes of epidermal (seam) cells at the bean stage demonstrated that *dlg-1* mRNA did not only colocalize with the *Ce*AJ but was also present laterally (Fig. S3B). The lateral localization of *dlg-1* mRNA diminished at later stages of development (comma stage) in favor of a more consistent colocalization with the fully mature *Ce*AJ (Fig. S3C).

Morphogenesis of the digestive track showed a different pattern for *dlg-1* and *ajm-1* mRNA junctional localization (Fig. 2C,D). Visually, during foregut or pharyngeal *Ce*AJ (p*Ce*AJ) maturation at the 16E stage, and after full formation at the bean stage, *dlg-1* and *ajm-1* mRNA were only mildly colocalized with *Ce*AJ markers (Fig. 2C). Only at the comma stage, when p*Ce*AJ were fully established, was a higher degree of localized mRNA observed, especially for *ajm-1* mRNA [61% for *dlg-1* and 68% for *ajm-1* (negative control, *jac-1*: 29%); Fig. 2C,D; Table S2]. At later stages of pharyngeal morphogenesis (1.5-fold stage), as observed for the epidermis, mRNA enrichment at the p*Ce*AJ decreased gradually [47% for *dlg-1* and 58% for *ajm-1* (negative control, *magi-1*: 20%); Fig. 2C,D; Table S2]. These data demonstrate enrichment at the *Ce*AJ for two of our identified localized mRNAs at distinct stages and cell types of embryogenesis.

### Localization of *dlg-1* mRNA at the *Ce*AJ does not depend on its UTRs

**Fig. 3.**
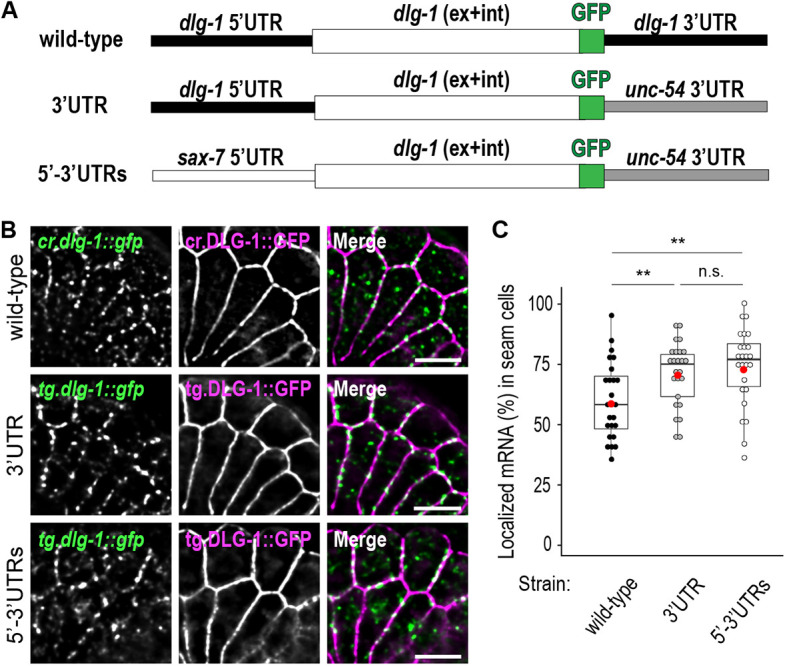
***dlg-1* endogenous 5′ and 3′UTR are not required for its localization.** (A) Schematic representations of the three analyzed transgenes carrying a GFP-tagged *dlg-1* [comprising exons (‘ex’) and introns (‘int’) combined with endogenous or exogenous UTRs that are not competent to localize their own mRNAs. Wild type indicates a CRISPR-engineered line with endogenous *dlg-1* 5′ and 3′UTRs (black). 3′UTR indicates a multicopy extrachromosomal transgenic line with *dlg-1* 5′UTR (black) and *unc-54* 3′UTR (gray). 5′-3′UTRs indicate a multicopy extrachromosomal transgenic line with *sax-7* 5′UTR (white) and *unc-54* 3′UTR (gray). The schematics are not to scale relative to the actual size of the corresponding sequences. UTR lengths: *dlg-1* 5′UTR, 61 nucleotides; *sax-7* 5′UTR, 63 nucleotides; *dlg-1* 3′UTR, 815 nucleotides; *unc-54* 3′UTR, 280 nucleotides. (B) Fluorescent micrographs of a lateral region of seam cells and ventral epithelial cells at the comma stage of *C. elegans* embryos showing smFISH signal of CRISPR or extrachromosomal transgenic *dlg-1* mRNAs (*cr.dlg-1::gfp* and *tg.dlg-1::gfp*, respectively, green), fluorescent signal of CRISPR or extrachromosomal transgenic GFP-tagged DLG-1 protein (cr.DLG-1::GFP and tg.DLG-1::GFP, magenta) and merges. Scale bars: 5 µm. (C) Box and whisker plot. Each dot represents the percentage of laterally localized versus total cellular *dlg-1::gfp* mRNA in ‘wild-type’ [black; mean=60.13; standard deviation (StDev)=15.70], ‘3′UTR’ (gray; mean=70.95; StDev=13.16) and ‘5′-3′UTRs’ (white; mean=73.85; StDev=16.18) (*y*-axis) in each seam cell analyzed at comma stages (*n*=25 for each transgenic line). Data are derived from five different embryos. Significance of statistical analyses (*t*-test, one tailed): n.s., not significant; **P<0.01. A thick black line represents the median, the two hinges represent first and third quartiles, the two whiskers define the upper and lower limits, and dots represent individual results. Red dots represent the mean.

mRNA localization commonly involves recognition of zip-codes located within UTRs (Chaudhuri et al., 2020; Jambhekar and Derisi, 2007). To test whether the localization of one of the identified localized mRNAs, *dlg-1*, relied on zip-codes, we generated extrachromosomal transgenic lines carrying a *dlg-1* gene whose sequence (exons and introns) was fused to an in-frame GFP and to endogenous or exogenous UTRs (Fig. 3A). We used UTRs from mRNAs that do not localize near cell membranes, namely *sax-7* and *unc-54* (Fig. S4A,B). One construct (‘3′UTR’ reporter) substituted the endogenous *dlg-1* 3′UTR with an *unc-54* 3′UTR (Lockwood et al., 2008; McMahon et al., 2001), and a second construct exchanged both the endogenous 5′ and 3′UTRs (‘5′-3′UTRs’ reporter) by additionally substituting the endogenous *dlg-1* 5′UTR with a *sax-7* 5′UTR to the 3′UTR reporter construct (Fig. 3A). The transgenic constructs were expressed in a wild-type background, and smFISH experiments were conducted with probes against the GFP RNA sequence to assess specifically the localization of the transgenic *dlg-1::gfp* mRNAs (*cr.dlg-1::gfp* and *tg.dlg-1::gfp*). The mRNA localization patterns of the two UTR reporters were compared with the localization of *dlg-1::gfp* transcripts from the CRISPR line (‘wild-type’, Fig. 3A; Heppert et al., 2018). Both reporter strains were enriched at the *Ce*AJ and resembled the wild-type *cr.dlg-1::gfp* (means: wild-type=60%; 3′UTR=71%; 5-3′UTR=74%; Fig. 3B,C; Table S2). A slight increase in mRNA localization for the two reporter strains may reflect their different transgenic nature (extra-chromosomal) compared with the wild-type reference (CRISPR). These results indicate that the UTR sequences of *dlg-1* mRNA are not required for localization.

### Localization of *dlg-1* mRNA at the *Ce*AJ is translation dependent

**Fig. 4.**
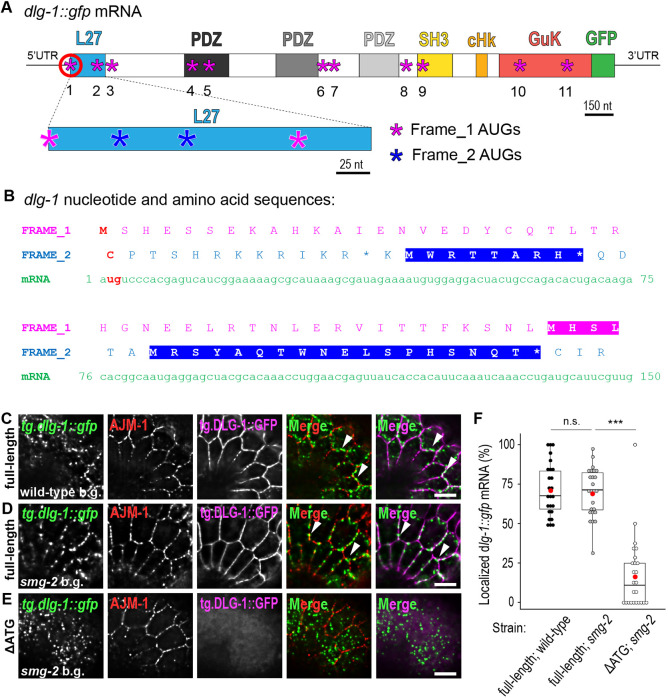
***dlg-1* mRNA localization depends on its translation.** (A) Upper part: schematic representation of transgenic *dlg-1::gfp* mRNA and domain-coding regions. Magenta asterisks indicate the 11 possible in-frame AUGs along the coding sequence. Red circle indicates the first AUG whose corresponding TG nucleotides were elicited from the transgenic ‘ΔATG’ sequence. Scale bar: 150 nucleotides (nt). UTRs and GFP are not in scale. Lower part: zoom-in of the L27-coding sequence. Magenta asterisks indicate the first two AUGs belonging to the main frame (‘Frame_1’). Blue asterisks indicate the first two AUGs out of frame (‘Frame_2’). Scale bar: 25 nucleotides (nt). (B) Nucleotide sequence (green) and its corresponding amino acid translation for the first (magenta) and the second (blue) frames. The amino acid sequence highlighted in blue represents full coding sequences (from a methionine to a stop codon) that are out of frame (‘Frame_2’). The amino acid sequence highlighted in magenta represents the first alternative in frame sequences in the transgenic ‘ΔATG’. The two nucleotides elicited from the transgenic ‘ΔATG’ sequence and the corresponding amino acid that cannot be translated in the two frames are indicated in red. (C-E) Fluorescent micrographs of multicopy extrachromosomal transgenic lines of a lateral region of seam and epidermal cells at a comma stage of *C. elegans* embryos. smFISH signal of wild-type (‘full-length’; C,D) and altered ATG (‘ΔATG’, line 1; E) *tg.dlg-1::gfp* mRNAs (green), immunofluorescent signal of the endogenous AJM-1 protein (red), fluorescent signal of the corresponding tg.DLG-1::GFP protein (magenta) and merges. Corresponding genotypes are at the bottom: the ‘full-length’ transgene in C is expressed in a wild-type background (‘wild-type b.g.’); ‘full-length’ and ‘ΔATG’ transgenes in D and E are expressed in a null mutant background for an NMD component (‘*smg-2* b.g.’). Arrowheads indicate examples of localized mRNA. Scale bars: 5 µm. (F) Box and whisker plots. Each dot represents the percentage of laterally localized versus total cellular *dlg-1::gfp* in ‘full-length; wild-type’ (black; mean=71.74; StDev=16.88), ‘full-length; *smg-2*’ (gray; mean=69.61; StDev=15.64) and ‘ΔATG; *smg-2*’ (white; mean=17.50; StDev=22.80) mRNAs (*y*-axis) in each seam cell analyzed at comma stages (*n*=25). Data are derived from five different embryos. Significance of statistical analyses (*t*-test, two tails for ‘full-length; wild-type’ versus ‘full-length; *smg-2*’, one tail for ‘full-length; *smg-2*’ versus ‘ΔATG; *smg-2*’): n.s., not significant; ***P<0.001. A thick black line represents the median, the two hinges represent first and third quartiles, the two whiskers define the upper and lower limits, and dots represent individual results. Red dots represent the mean.

Co-translational mechanisms for mRNA delivery have been described for mRNA encoding transmembrane and secreted proteins (Nyathi et al., 2013). Recent studies have suggested that co-translational mRNA localization can also exist for transcripts encoding proteins in other subcellular locations (Chouaib et al., 2020; Li et al., 2021; Hirashima et al., 2018; Safieddine et al., 2021; Sepulveda et al., 2018). To determine whether *dlg-1* mRNA localization occurs co-translationally, we designed a transgene (tg) to interfere with normal translation by deleting two nucleotides (TG) within the start codon of an otherwise wild-type sequence that contained both exons and introns (Fig. 4A,B). Ribosomes scanning transgene mRNA from the 5′ end would encounter two new AUG start codons that are each out-of-frame compared with the wild type (Fig. 4B). The first in-frame AUG after the deletion is located at position 47 (Fig. 4A,B and Fig. S5A; Table S3). We generated transgenic lines in a *smg-2* mutant strain (Hodgkin et al., 1989) to avoid mRNA degradation by nonsense-mediated decay (NMD), which recognizes and destroys mRNAs with precocious translation termination (Huang and Wilkinson, 2012; Mango, 2001). As a control, we verified by smFISH that wild-type *tg.dlg-1* mRNA was localized normally in *smg-2* mutant embryos, demonstrating that NMD does not interfere with targeting *dlg-1* transcripts to the *Ce*AJ (Fig. 4C,D,F).

Next, we examined our non-translatable *dlg-1::gfp* mRNA (‘ΔATG’) in *smg-2* mutant embryos using smFISH paired with fluorescence analysis for DLG-1::GFP protein, to tract the degree of in-frame translation. The C-terminal position of the GFP moiety ensures that mRNAs that initiate in-frame translation anywhere within the DLG-1-coding sequence are GFP positive. AJM-1 antibody staining was used to identify the *Ce*AJ (Francis and Waterston, 1991; Mohler et al., 1998). The embryos of one of our ΔATG transgenic lines (line1) lacked any detectable DLG-1::GFP protein and displayed a dramatic decrease of mRNA at the *Ce*AJ compared with controls (means: full-length; wild-type=72%; full-length; *smg-2*=70%; ΔATG; *smg-2*=18%; Fig. 4E,F; Table S2). We conclude that translation is required for mRNA localization. Embryos from our second ΔATG transgenic line (line 2) displayed a little GFP protein (Fig. S5B,C). We speculate that truncated DLG-1 protein may be generated by one or more of the ten alternative in-frame AUGs that can be found within the *dlg-1* mRNA (Fig. 4A and Fig. S5A; Table S3). For the second line, we observed some mRNA localized at the *Ce*AJ, and it was always in proximity to DLG-1::GFP protein (Fig. S5B). These data suggest that *dlg-1* mRNA localization depends on its ongoing translation (e.g. line 1; Fig. 4E,F), and that even low amounts of translation are sufficient for mRNA delivery to its final location (e.g. line 2; Fig. S5B).

As a second test for the involvement of translation in *dlg-1* mRNA localization, we inhibited total translation in a drug-free manner. Drugs like cycloheximide or puromycin, commonly used to block translation, do not penetrate the worm embryo eggshell easily. Instead, we exposed the *C. elegans* embryos to heat. One of the early responses to heat stress is a block of translation caused by ‘ribosome drop-off’ (Spriggs et al., 2010), leading to a global decrease in polysome occupancy (Arnold et al., 2014). Embryos from our *dlg-1::gfp* CRISPR line grown at 20°C for generations were subjected to a 1 h heat-shock at 34°C or 37°C on plates and immediately processed for smFISH experiments (Fig. S6A-C). In both conditions, we observed a significant loss in mRNA localization at the *Ce*AJ (means: 20°C=65%; 34°C=27%; 37°C=19%; Fig. S6D and Table S2). These results show the loss of mRNA localization upon heat shock. In both the ATG deletion strains and the heat-shock conditions, endogenous DLG-1 was present at the *Ce*AJ (Fig. S6 and data not shown). Therefore, we conclude that translation of *dlg-1* mRNA in *cis* is required for enrichment at the *Ce*AJ.

### The C-terminal region is necessary and key for mRNA localization to the membrane

**Fig. 5.**
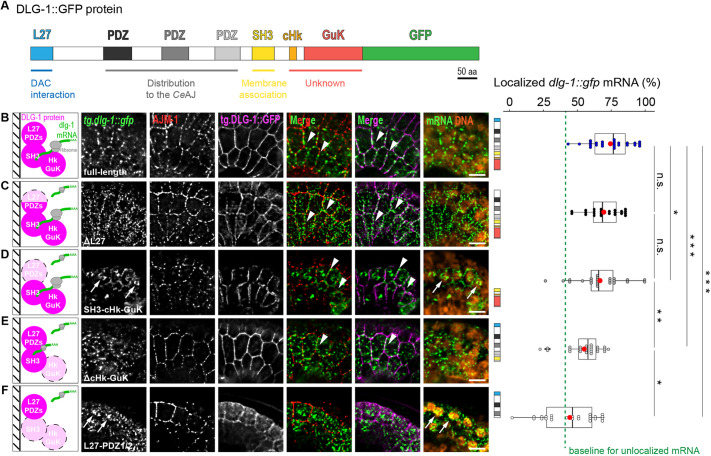
**Specific domain-coding sequences of *dlg-1* mRNA are required for its normal lateral localization.** (A) Schematic representation of the full-length transgenic DLG-1::GFP protein, highlighting domains and their known functions (Firestein and Rongo, 2001). Blue indicates L27 domain. Shades of gray indicate the three PDZ domains. Yellow indicates the SH3 domain. Orange indicates the conserved stretch of the Hook domain (cHk). Red indicates the GuK domain. Green indicates GFP, which is C-terminally tagged. Scale bar: 50 amino acids (aa). (B-F) Left: schematic representations of wild-type (‘full-length’; B) and truncated versions of DLG-1 [ΔL27 (C), SH3-cHk-GuK (D), ΔcHk-GuK (E) and L27-PDZ1/2 (F)]. Domains that are present in the transgene are depicted in magenta, deleted ones are in light pink surrounded by black dashed lines. mRNAs undergoing translation (with ribosomes in gray) whose size represents a rough estimation of their abundance, quantified at the very right of each panel, are indicated in green. Small black parallel lines on the left of the schematics represent the lateral cell membrane (*Ce*AJ included). Top views of fluorescent micrographs (maximum intensity projection of seven *z*-stacks) of a lateral region of seam and epidermal cells at the bean stage of *C. elegans* embryos showing smFISH signal of transgenic *tg.dlg-1::gfp* mRNAs (green), immunofluorescent signal of the endogenous AJM-1 protein (red), fluorescent signal of the transgenic GFP-tagged DLG-1 protein coded by the corresponding transgene (magenta) and merges. The right-most merge images show mRNA with DNA (orange) to mark the nuclei. Arrowheads indicate examples of laterally localized mRNAs. Arrows indicate examples of fluorescent mRNA signal of overexpressed transgenes in the nucleus. Scale bars: 5 µm. Horizontal box and whisker plot. Each dot represents the percentage of laterally localized versus cellular *tg.dlg-1::gfp* in the different lines analyzed (schematics of the domain structure as in A are on the left of each box plot): ‘full-length’ (blue; *n*=25; mean=74.22; StDev=15.09), ‘ΔL27’ (black; *n*=25; mean=69.21; StDev=12.33), ‘SH3-cHk-GuK’ (dark gray; *n*=25; mean=66.41; StDev=17.71), ‘ΔcHk-GuK’ (light gray; *n*=25; mean=54.22; StDev=13.81) and ‘L27-PDZ1/2’ (white; *n*=25; mean=43.40; StDev=20.19) mRNAs (*y*-axis) in each seam cell analyzed at bean stages. Data are derived from five different embryos for each line (six for SH3-cHk-GuK). A vertical green dashed line represents the baseline of localization (40.98%) for an unlocalized mRNA, *jac-1*, determined with the same method used for the transgenic lines (see Fig. S7 and Table S2 for details). Significance of statistical analyses (*t*-test, one tail): n.s., not significant; *P<0.05; **P<0.01; ***P<0.001. A thick black line represents the median, the two hinges represent first and third quartiles, the two whiskers define the upper and lower limits, and dots represent individual results. Red dots represent the mean.

Given the requirement for translation to localize *dlg-1* mRNA, we considered mRNA targeting in the context of the DLG-1 protein that would be produced. DLG-1 is a complex protein, with different domains that establish protein localization and function, as diagrammed in Fig. 5A (Firestein and Rongo, 2001). To define critical regions for mRNA localization, we deleted these domains using existing (Firestein and Rongo, 2001; Lockwood et al., 2008) and newly generated transgenic lines (see Materials and Methods) (Table S4). Immunostaining for endogenous AJM-1 provided a spatial reference for the *Ce*AJ that was unaffected in any of our transgenic strains, which also expressed endogenous wild-type DLG-1. smFISH with GFP probes were specific for transgenic *dlg-1*::GFP (‘*tg.dlg-1*’) and GFP fluorescence from transgenic DLG-1 (‘tg.DLG-1’) provided a readout for the localization of the transgenic protein. We began by analyzing images of top views of epidermal seam cells to determine association to lateral membranes. We focused on the bean stage, when wild-type *dlg-1* RNA is highly localized (Fig. 5B-F and Fig. 2). Fluorescent images and quantitation of our full-length (FL) control revealed lateral enrichment of *tg.dlg-1^FL^* mRNA (Fig. 5B; mean=74%; Table S2). Such lateral and *Ce*AJ enrichment of *tg.dlg-1^FL^* mRNA resembled what was observed with the CRISPR line, indicating that transgenes reflected appropriate regulation (Fig. S3B).

First, we examined the N-terminal domains. The L27 protein domain is involved in DLG-1 multimerization as well as interactions with the DAC component, AJM-1 (Firestein and Rongo, 2001; Lockwood et al., 2008). Removal of the sole L27 domain (ΔL27; Fig. 5C) did not significantly impair the lateral enrichment of *tg.dlg-1^ΔL27^* mRNA compared with the full length (Fig. 5C; mean=69%; Table S2). These data suggest that the L27 domain sequences make minor contributions to the accumulation of *dlg-1* mRNA to the lateral membranes. Given the interactions of the L27 domain with junctional proteins, this effect may reflect detachment of mutant DLG-1 protein and mRNA from junctions.

A larger N-terminal truncation removed the PDZ domains as well as the L27 domain, but left the SH3, Hook and GuK domains intact (SH3-cHk-GuK; Fig. 5D). *tg.dlg-1^SH3-cHk-GuK^* mRNA was also enriched laterally to a degree that was similar to the ΔL27 line (Fig. 5D; mean=66%; Table S2). These data suggest that the PDZ domains are not required for lateral mRNA enrichment. We speculate that the C-terminal sequences are largely sufficient to direct *dlg-1* mRNA to lateral membranes.

We examined a construct lacking the Hook and GuK domains (ΔcHk-GuK; Fig. 5E), and observed a significant decrease in mRNA lateral localization compared with both the full-length and the SH3-cHk-GuK construct (Fig. 5E; mean=54%; Table S2), but higher than those of an unlocalized mRNA (Fig. S7A,B; mean=41%; Table S2). These data suggest that Hook and GuK domains are key for *dlg-1* mRNA localization to the lateral membrane, but that additional, N-terminal sequences contribute somewhat. As the Hook and GuK domains have no known role in protein localization (Lockwood et al., 2008), these data reveal a new role for these sequences in localization.

This hypothesis was confirmed when we examined another C-terminal truncation where the SH3 and third PDZ domain were removed in addition to the C-terminal part (L27-PDZ1/2; Fig. 5F). *tg.dlg-1^L27-PDZ1/2^* mRNA lateral localization was highly impaired and reached the levels of an unlocalized mRNA (Fig. 5F and Fig. S7B; mean=43%; Table S2). Together with the previous data, these observations indicate that the Hook and GuK domains are essential for *dlg-1* mRNA localization and account for the large majority of lateral membrane localization of *dlg-1* mRNA, in conjunction with the SH3 domain.

### The N-terminal region is involved in mRNA enrichment at the junction once the mRNA has reached the membrane

**Fig. 6.**
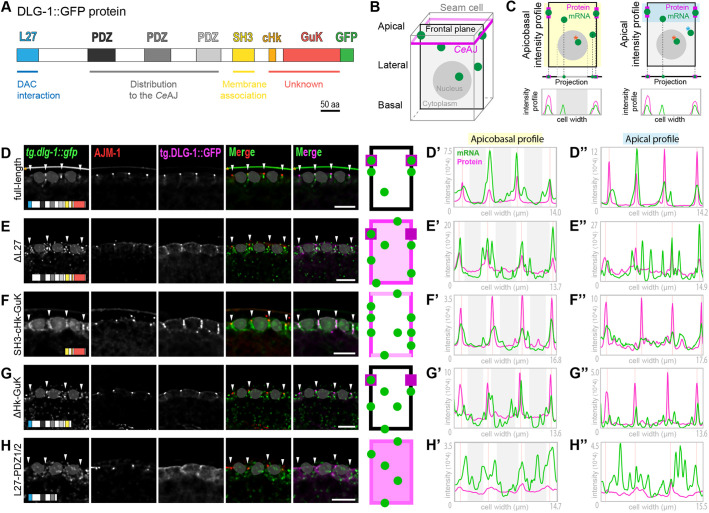
**Specific domain-coding sequences of *dlg-1* mRNA are required for its normal apicobasal localization.** (A) Schematic representation of the full-length transgenic DLG-1 protein as in Fig. 5A. GFP sequence is not to scale. (B) Schematic representation of a seam cell in 3D (gray cube). Magenta apical belt: *Ce*AJ. A black rectangle shows a frontal plane view in the middle of the cell used to analyze the images in the rest of the figure. Light gray represents the cytoplasm, a dark-gray filled circle represents the nucleus and green filled circles represent mRNAs. (C) Top: simplified schematics of the frontal view of a seam cell (B). Green circles indicate transgenic *dlg-1* mRNA. Magenta rectangles indicate transgenic DLG-1 protein. Highlighted in yellow (top left) and in blue (top right) are the regions of the cell used for apicobasal and apical intensity profile analyses, respectively. Orange asterisks indicate mRNAs in the nuclei that have not been considered in the intensity profile analyses as representing transcription sites. Blue asterisk indicates an example of a cytoplasmic mRNA that would be considered in the apicobasal analysis, but not in the apical. Projections of mRNA and protein (and nucleus in the left side) present in the schematics above (same color-code) are shown below. Intensity profile graphs are shown at the bottom, based on the projections above, where peaks show the positions of transgenic *dlg-1* mRNA (green line) and transgenic DLG-1 protein (magenta). *x*-axis, width of the cell; *y*-axis, fluorescence intensity. The gray box in the left graph represents the projection of the nucleus whose intensities have been removed from the analysis. (D-H) Frontal plane views of fluorescent micrographs of three adjacent seam cells at the bean stage of *C. elegans* embryos showing smFISH signal of transgenic *tg.dlg-1-gfp* mRNAs [full-length (D), ΔL27 (E), SH3-cHk-GuK (F), ΔcHk-GuK (G) and L27-PDZ1/2 (H) (green)], immunofluorescent signal of the endogenous AJM-1 protein (red), fluorescent signal of the corresponding transgenic GFP-tagged DLG-1 protein (magenta) and merges. Arrowheads indicate *Ce*AJ localization. Shaded gray shapes cover the nuclear regions to avoid focusing on transcriptional or general nuclear mRNA signals not relevant to the study. Orange asterisks indicate nonspecific signal staining the eggshell. Scale bars: 5 µm. Simplified schematics based on the fluorescent images are on the right. Frontal view of a seam cell (rectangle) modelling transgenic mRNA and protein localizations. Green circles indicate transgenic *dlg-1* mRNA. Shades of magenta indicate varying degrees of transgenic DLG-1 protein along the membrane (borders) and in the cytoplasm (middle part). (D′-H′) Intensity profile graphs of three contiguous cells [apicobasal profile (highlighted in yellow) explained in C (left)] for the corresponding fluorescent images in D-H). *x*-axis, cell width (µm); *y*-axis, measured fluorescent intensities. Green lines indicate transgenic *dlg-1* mRNA intensities. Magenta lines indicate transgenic DLG-1 protein intensities. Pink vertical lines indicate location of the *Ce*AJ, identified by peak values for the intensity profile of AJM-1 fluorescent signal (not shown). Light-gray panels indicate nuclei locations that have been evicted from the mRNA channel to avoid quantification of transcriptional signal, corresponding to the localization of the shaded gray shapes in the fluorescent images. (D″-H″) Intensity profile graphs of the sole apical part of the same cells analyzed on the left [apical profile (highlighted in blue) explained in C (right)]. Axes and color codes are as in D′-H′.

We examined the apicobasal and junctional distribution of *dlg-1* mRNAs, by analyzing frontal plane views, paired to apicobasal and apical intensity profile analyses (Fig. 6). Fluorescent images of our full-length control showed lateral and *Ce*AJ enrichment of *tg.dlg-1^FL^* mRNA and *Ce*AJ localization for the corresponding tg.DLG-1^FL^ protein (Fig. 6D). These data were confirmed by intensity profile analyses where mRNA peaks (green) largely overlapped with protein peaks (magenta), and these were located at the *Ce*AJ (pink vertical lines) in both apicobasal and apical profiles (Fig. 6D′,D″). A few minor mRNA peaks were observed in the apicobasal intensity profile between the *Ce*AJ peaks, indicating a few cytoplasmic mRNAs that might represent mRNA in transit from the nucleus to their final location (Fig. 6D′). Other RNAs were observed close to the *Ce*AJ peaks in the apical intensity profile (Fig. 6D″). These data indicate that the bulk of *dlg-1* mRNA is associated with the *Ce*AJ and lateral surfaces.

Next, we analyzed the same transgenic lines examined in Fig. 5, starting from the N-terminus. Deletion of the L27 domain (involved in DLG-1 multimerization and AJM-1 interaction; Firestein and Rongo, 2001; Lockwood et al., 2008) showed tg.DLG-1^ΔL27^ protein localized more broadly along the whole membrane compared with the full-length mRNA, with only partial enrichment at the *Ce*AJ (Fig. 6E). Similar to its encoded protein, *tg.dlg-1^ΔL27^* mRNA distribution was scattered along the whole membrane (Fig. 6E). Intensity profile analyses confirmed the broader membrane distribution of tg.DLG-1^ΔL27^ and the presence of *tg.dlg-1^ΔL27^* mRNA along the lateral (and partially apical and basal) membranes. Some *tg.dlg-1* mRNA was enriched at the *Ce*AJ, but less than the full-length, and some was cytoplasmic mRNA, more than the full-length (Fig. 6E′,E″). These data suggest that the L27 domain contributes to the accumulation of *dlg-1* mRNA and protein at the *Ce*AJ.

The larger N-terminal truncation, leaving the sole SH3, Hook and GuK domains intact, showed a broader distribution of tg.DLG-1^SH3-cHk-GuK^ protein at the lateral membrane without any enrichment at the *Ce*AJ. *tg.dlg-1^SH3-cHk-GuK^* mRNA was also distributed along the membrane, especially laterally (Fig. 6F,F′). This broader distribution along the whole membrane could not be addressed with the sole top views (Fig. 5). The apicobasal view suggests that the mRNA not seen laterally in Fig. 5 may reflect localization at apical or basal membranes. These data show that the C-terminal sequences are sufficient to direct *dlg-1* RNA and protein to the membrane (as also seen in Fig. 5D), but that the N-terminal L27 and PDZ domains are important for targeting mRNA and protein to the *Ce*AJ (Fig. 6F,F″).

The correlation between mRNA and protein localization for the constructs described above suggests that either protein dictates mRNA localization or translation occurs at defined locations. This correlation was lost for constructs lacking the Hook and GuK domain (Fig. 6G,G′,G″). Deletion of the Hook and GuK domains did not affect tg.DLG-1^ΔcHk-GuK^ protein localization but did impair *Ce*AJ enrichment of mRNA (Fig. 6G,G′,G″). The mRNA was detected at all membrane surfaces: lateral, apical and basal (Fig. 6G). Other mRNA was detected adjacent to the membrane, but not overlapping, and in the cytoplasm (Fig. 6G,G′). Similar to Fig. 6F, this apicobasal view suggests that mRNA that was not located laterally in top views may also reflect its location at the apical and basal membranes. These data suggest that sequences within the Hook and GuK domains help target *dlg-1* mRNA to the membrane. DLG-1 protein localized at the junction in this mutant strain may reflect localized translation for the subset of *dlg-1* mRNAs at lateral and junctional surfaces, or post-translational movement of protein to the junction.

Further removal of sequences from the C-terminal part impaired tg.DLG-1 protein and *tg.dlg-1* mRNA localization. The construct containing the L27 and the first two PDZ domains produced transgenic protein and mRNA present all over the membrane and in the cytoplasm (Fig. 6H). In intensity profile analysis, mRNA and protein peaks showed minimal overlap, confirming the broader and more randomized distribution observed in the fluorescent images (Fig. 6H′,H″). These data suggest that *dlg-1* mRNA and protein can accumulate in ectopic locations without degradation, at least for the N-terminal half of the protein.

In conclusion, the structure-function analyses revealed that the Hook and GuK domains were required to localize *dlg-1* mRNA and, together with the SH3 domain, were sufficient for both protein and mRNA localization to lateral membranes. In addition, PDZ domains together with the L27 were necessary, but not sufficient, to bring DLG-1 and *dlg-1* to the *Ce*AJ (Figs 5 and 6).

## DISCUSSION

This study has made three contributions towards understanding RNA localization in *C. elegans* embryos. First, the cytoplasmic mRNAs within adhesion system II of the *Ce*AJ are localized within epithelial cells. Second, localization of *dlg-1* mRNA depends on translation in *cis*, and not on UTR zip-codes. Third, specific regions within the *dlg-1*/DLG-1 C-terminal domain dictate localization at the membrane.

We conducted an smFISH-based survey, which identified endogenous mRNAs that are localized at or near the cell membrane (five out of 25 tested mRNAs). Localized mRNA in *C. elegans* has been observed for maternally provided mRNAs in early embryos (Parker et al., 2020) and for RNAs in synapses of adult neurons (Yan et al., 2009). Very recently, a new study addressed membrane-associated localization of mRNA through a PP7/PCP-tagging approach in later embryonic stages and found five mRNA (*erm-1*, *pgp-1*, *magu-2*, *let-413* and *ajm-1*) to be enriched at these loci (Li et al., 2021). Previous studies of *Drosophila* embryogenesis have shown that many mRNAs are localized subcellularly, including mRNAs coding for cytoskeletal and junctional components (Jambor et al., 2015; Lecuyer et al., 2007; Ryder and Lerit, 2018). Among them, β-actin, E-cadherin and *zo-1* mRNAs are localized at the cell cortex of epithelial cells (Ryder and Lerit, 2018). We did not observe orthologues of these mRNAs being cortically localized, suggesting species- or cell type-specific differences. Nevertheless, we identified other localized mRNAs, four of which (*dlg-1*, *ajm-1*, *erm-1* and *sma-1*) encode factors that are functionally linked within the AS-II. The transmembrane protein SAX-7 is also supposedly localized in the AS-II, but its mRNA was not membrane associated. Instead, we observed perinuclear *sax-7* mRNA, suggesting SAX-7 is localized via the ER.

mRNAs coded by orthologues of *dlg-1* also show a defined subcellular localization in other species in polarized cells such as embryonic cells or neurons. For example, *Drosophila dlg1* mRNA associates transiently with membranes during embryogenesis (mitotic cycle 14): laterally at stage 5 (cellularization), when membranes start to form around nuclei; and all around the cell membrane at stages 6 and 7 (cellularization and gastrulation). It becomes unlocalized at later stages (Lecuyer et al., 2007). In zebrafish, neuronal *dlg1* mRNA localizes stably in myelin sheets of fully differentiated oligodendrocytes (Yergert et al., 2021). These data suggest that *dlg1* mRNA localization may have pivotal roles in development. Although *dlg-1* is the most broadly studied, one other mRNA uncovered in our survey, *erm-1*, has been shown to localize in another context. Specifically, *erm-1* mRNA is targeted to the cell periphery in the early *C. elegans* embryo (Parker et al., 2020), providing an additional example of membrane enriched mRNA in different developmental contexts.

Localization of *dlg-1* mRNA depended on its translation rather than its UTRs. Substitution of both the 5′ and 3′UTRs with UTRs of unlocalized mRNAs did not disrupt association of *dlg-1* mRNA with the *Ce*AJ. This result mirrors orthologues of *dlg-1* in other species, which also do not require their UTRs for subcellular localization (Yergert et al., 2021). Thus, *dlg1* mRNAs are frequently localized within cells, but the mechanism of UTR-independent targeting of this transcripts is unknown for any species. We found that *dlg-1* mRNA required its coding sequences and translation in *cis*. We analyzed two *C. elegans* lines expressing a full-length *dlg-1*::GFP with the normal ATG deleted (ΔATG). One of these produced no protein and had no mRNA localization, demonstrating the importance of translation. This line demonstrated that DLG-1 protein supplied in *trans* was not sufficient for targeting to the *Ce*AJ, as all our transgenic lines were analyzed in embryos expressing wild-type, endogenous DLG-1. The second line fortuitously produced a little protein in some cells. In these expressing cells, both protein and mRNA were occasionally localized, suggesting that even a little translation was sufficient to target *dlg-1* mRNA to the membrane and *Ce*AJ.

Besides the ATG mutation, the remainder of the *dlg-1^ΔATG^* gene was wild type, including the intron-exon sequences. This wild-type configuration reveals that sequences and complexes associated with the EJC are not sufficient to localize *dlg-1* mRNA. Thus, *dlg-1* likely differs from mRNAs such as *oskar* in *Drosophila*, where splicing generates a localization element and EJC binding site, which together target *oskar* mRNA within oocytes (Ghosh et al., 2012).

Our structure and function analyses highlighted two pathways for mRNA localization: one dependent on the C-terminal part (SH3, Hook and GuK domains) and a second dependent on the N-terminus (L27-PDZ domains). The first pathway relied on protein sequences that are known to target and maintain DLG-1 protein at the membrane (SH3) and at the Hook and GuK domain with unknown function. A minimal sequence containing these three domains accounted for the vast majority of *dlg-1* mRNA localized at the membranes (mostly lateral). Complementarily, loss of SH3, Hook and GuK domains (together with the third PDZ) fully impaired *dlg-1* mRNA localization. We note that the location of the Hook and GuK domains at the C-terminal part of DLG-1 demonstrates that *dlg-1* mRNA is not delivered analogously to ER targeting, where translation is arrested after translation of N-terminal signal sequences and resumes when mRNAs are docked at the ER. However, a modified co-translational pathway is a possibility. For example, after the SH3 domain is translated, the translating complex, including the mRNA, could be delivered to the lateral membranes even in the absence of a full round of translation. This model fits with the known requirement of the SH3 domain for lateral distribution of DLG-1 protein (Lockwood et al., 2008), but does not yet explain the role of the Hook and GuK.

Loss of Hook and GuK sequences showed normal protein localization even in a mutant background. Because a small proportion of this mutant mRNA was found at or near lateral membranes, it is possible that only this cohort was translated. This model would explain the lack of mutant DLG-1 protein observed near mislocalized mRNA. Alternatively, mutant DLG-1 could be translated ubiquitously in epidermal cells and then transported rapidly to the *Ce*AJ. We note that previous studies have shown that the Hook and GuK domains are essential for viability, but the reason is unknown (Lockwood et al., 2008). One intriguing possibility is that the lack of mRNA localization affects DLG-1 levels or turnover. Alternatively, these domains have other physiological roles that have not yet been discovered.

Once at the lateral membrane, a second step may deliver mRNA and protein to the CeAJ. For example, DLG-1 associates with itself and with AJM-1 via the L27 domain, and the PDZ domains are involved in DLG-1 junctional association (Lockwood et al., 2008). Thus, these N-terminal sequences may either target DLG-1/*dlg-1* from lateral surfaces to the *Ce*AJ or retain DLG-1 at the junction.

In summary, our findings suggest a two-step model for targeting a translating *dlg-1* mRNA to the *Ce*AJ: first, the C-terminal part of the DLG-1 protein brings the translating complex to the membranes, then the N-terminal region pushes it apically to the *Ce*AJ. None of these domain combinations was sufficient to target *dlg-1* mRNA to the junction, suggesting that these processes synergize to place *dlg-1* mRNA at the *Ce*AJ during embryonic epithelium formation.

## MATERIALS AND METHODS

### Nematode culture

All animal strains were maintained as previously described (Brenner, 1974) at 20°C. Transgenic lines containing extrachromosomal arrays were grown at 15°C to reduce transgene overexpression. Some lines containing extrachromosomal arrays presented instances of mosaicism and differential expression levels among cells due to their extrachromosomal nature. Therefore, we focused on cells with consistent patterns of expression. For a full list of alleles and transgenic lines, see Table S4.

### Heat-shock experiments

Heat-shock experiments (duplicates) were performed on ML2615: the agar of each plate, with a high amount of laid embryos, was split into three new plates. Each new plate was placed for 1 h at the three different temperatures for the experiment (20°C, 34°C and 37°C). Embryos were then collected and processed following the smFISH protocol.

### Generation of transgenic lines

*dlg-1* deletion constructs ΔATG (SM2664 and SM2663) and ΔL27-PDZs (SM2641) were generated by overlap extension PCR using pML902 as a template. Oligos used to generate ΔATG: PCR1_Fw, agaggatccagctccacactaac; ΔATG_PCR1_Rv, tgactcgtgggatgcttccttcttcgg; ΔATG_PCR2_Fw, agaaggaagcatcccacgagtcatcgg; and PCR2_Rv, cgtacggccgactagtaggaaac. Oligos used to generate ΔL27-PDZs: PCR1_Fw, agaggatccagctccacactaac; ΔL27-PDZs_PCR1_Rv, tcaaaaatttgatactccatgcttccttcttcggtgagg; ΔL27-PDZs_PCR2_Fw, cgaagaaggaagcatggagtatcaaatttttgagtccaaaattgagaagct; and PCR2_Rv, cgtacggccgactagtaggaaac. For *dlg-1* 5′UTR replacement construct (“5′-3′UTRs”, SM2646), *sax-7* 5′UTR fragments were synthesized as ultramer duplex oligos ordered from IDT (sequence: aatttaattttttcaattttcaggatagaaaaagagtatcgaacgaagttcgacgcgattctagatcacgtcgaaagaccaccatcatgtcccacga). Oligos used to generate 5′-3′UTRs: PCR1_Fw, agaggatccagctccacactaac; 5′-3′UTRs_PCR1_Rv_a, tcgttcgatactctttttctatcctgaaaattgaaaaaattaaattgatcaacaagtttttgagact; 5′-3′UTRs_PCR1_Rv_b, tcgtgggacatgatggtggtctttcgacg; 5′-3′UTRs_PCR2_Fw, agaccaccatcatgtcccacgagtcatcgga; and PCR2_Rv, cgtacggccgactagtaggaaac. For all the constructs, the following primers have been used to amplify the final product in a nested PCR: Nested_Fw, agctccacactaactgtttgtgt; and Nested_Rv, gactagtaggaaacagttatgtttggtatattgggaa. All PCR reactions were column purified (High Pure PCR Product Purification kit, 11732668001, Sigma-Aldrich). 2 ng/μl of purified PCR products were injected into either N2 (for ΔL27-PDZs and 5′-3′UTRs) or SM507 (for ΔATG) strains, along with 100 ng/μl of pRF4 plasmid as a selection marker to create transgenic lines with extrachromosomal arrays.

### smFISH, immunostaining and microscopy

smFISH experiments were all carried out in triplicate besides those for the heat-shock experiments (Fig. S6; duplicates). smFISH was adapted from Tsanov et al. (2016). Custom Stellaris smFISH probes labeled with Quasar 570 dye were designed against *par-3, par-6*, *hmp-1* and *erm-1* mRNAs using the Stellaris FISH Probe Designer (Biosearch Technologies). Probes against the other mRNAs were designed following the smiFISH approach, as previously described (Tsanov et al., 2016). Each open reading frame was run through the Oligostan script in RStudio and 12-24 IDT primary smFISH probes were ordered for each mRNA (100 µM in IDTE, pH 8.0; IDT). All probes were designed against the endogenous mRNA sequences, besides *dlg-1*, *pkc-3*, *hmp-2*, *spc-1*, *let-805* and *vab-10a*, the mRNAs of which were detected with *gfp* probes in their corresponding transgenic lines (Table S4). Exceptions to this are Figs S1A and S3A, where we used probes against the endogenous *dlg-1* mRNA. For a full list of primary probe sequences, see Table S5. Secondary probes (FLAP-Y) with a 5′-acrydite modification and a 3′-Atto565 or a 3′-Atto637 labels were ordered from IDT. An equimolar amount of each set of primary probes was pooled in a 1.5 ml Eppendorf tube and diluted five times with IDTE (pH 8.0) to reach a final concentration of 0.833 µM per probe. An *in vitro* pre-hybridization reaction was set up as follow: 4 µl of primary probe-set pool, 1 µl of secondary FLAP-Y probe, 2 µl of 10x NEBuffer 3 (B7003, New England Biolabs) and 13 µl of water were incubated in a thermocycler (EP950040025, Eppendorf) at 85°C for 3 min, 65°C for 3 min and 25°C for 5 min. Pre-hybridized FLAP-Y smFISH probes could be placed at 4°C for storage. One 6 cm plate with gravid adults and laid embryos with a thin bacteria lawn left were washed with 1 ml of water. Adults and larvae were discarded. An additional 1 ml of water was added to the plate. Laid embryos were gently scrubbed off with a gloved finger and transferred to a 1.5 ml Eppendorf tube. A gentle ‘short’ 6 s spin was applied to the Eppendorf tube to pellet the embryos to minimize stress. Extra liquid was removed and embryos were allowed to rest for 10 min. Embryos were transferred on poly-L-lysine-coated (P8920, Sigma-Aldrich) slides (ER-303B-CE24, Thermo Scientific) and allowed to settle. Excess water was removed and a 50 µl fix (1% PFA in PBS with 0.05% Triton) was added and incubated for 15 min. After removing the fixative solution and adding a coverslip, slides were quickly transferred on a metal plate on dry ice and stored at −80°C overnight. After a freeze-crack, slides were immediately transferred in a Coplin jar with ice-cold methanol for 5 min. Subsequent washes of PBS (5 min), PBS with 0.5% Tween-20 (10 min and 20 min) and PBS again (5 min) were applied to the slides. 100 µl of hybridization solution [dextran sulfate (10% w/v) in one part formamide, one part 20×SSC and eight parts water) were applied to the sample area and slides were then transferred in a humidity chamber and incubated for 1 h at 37°C. After removal of the hybridization solution, 50 µl of new hybridization solution containing 1 µl of pre-hybridized FLAP-Y smFISH probes or 0.5 µl of Stellaris probes were applied to the sample area and slides were incubated again in a humidity chamber at 37°C for 4 h in the dark. After incubation, hybridization solution was wicked off, and samples were washed twice with wash buffer (one part formamide, one part 20×SSC and eight parts water). Slides with 100 µl of wash buffer were incubated for 1 h at 37°C in a humidity chamber in the dark. The wash buffer was finally wicked off and samples were washed twice with wash buffer and mounted with 12 µl Vectashield Antifade Mounting Medium with DAPI (H-1200, Vector Laboratories). We coupled AJM-1 antibody staining (MH27, DSHB, 1:100; Francis and Waterston, 1991) to our smFISH protocol. Primary antibodies were added to the hybridization solution during the 4 h incubation and secondary antibodies (Alexafluor 546 goat anti-mouse: A-11030, Invitrogen, 1:250) were added to the wash buffer in the last 1 h incubation. A widefield microscope FEI ‘MORE’ with total internal reflection fluorescence (TIRF) and a Hamamatsu ORCA flash 4.0 cooled sCMOS camera and a Live Acquisition 2.5 software was used for capturing images. Pictures were deconvolved with the Huygens software and then processed in OMERO (https://www.openmicroscopy.org/omero/) or ImageJ (https://imagej.net/). Figures were prepared in Adobe Illustrator (https://www.adobe.com/).

### Image analysis and quantitation

ImageJ (https://imagej.net/) was used for post processing [*z*-stack/channel extrapolation and transverse projections (as in Fig. S3B′,C′) and GFP intensity quantification (as in Fig. S5B)]. FISH-quant v.3 (Mueller et al., 2013) was used for image analysis and quantitation. The mRNA counts for each seam cell from top views in maximum intensity projections (Fig. S8A) were obtained by drawing outlines: (a) along the cell borders marked by DLG-1::GFP or AJM-1 (Fig. S89B,C) for ‘total’ mRNA (Fig. S8D); (b) parallel to the cell border for ‘cytoplasmic+nuclear’ mRNA (Fig. S8E,F); and (c) around the DNA marked by DAPI staining (Fig. S8G) for ‘nuclear’ mRNA (Fig. S8H). We implemented the FISHquant script (see Data availability section) to allow us to define units (single mRNAs) to estimate the amount of mRNA per identified dot. Units were identified by drawing outlines around five of the lightest mRNA dots and averaging their intensities (‘Amplitude’ in FISHquant; Fig. S8I). The intensities of each identified dot was divided by the intensity of our unit. The difference between ‘total’ and ‘cytoplasmic+nuclear’ mRNA provided the value for ‘membrane’ mRNA (enriched at or in the proximity of the lateral cell membrane). In all the analyses but the one for Fig. 5, the amount of localized mRNA has been calculated as ‘membrane’ mRNA divided by ‘total’ mRNA. In Fig. 5, owing to the high transcriptional signal in some of the transgenic lines, the amount of localized mRNA has been calculated as ‘membrane’ mRNA divided by ‘total’ minus ‘nuclear’ mRNA. All the lines, including positive (‘full-length’; Fig. 5B) and negative (*jac-1*; Fig. 5 and Fig. S7) controls, have undergone the same type of analysis. This type of calculation was made to avoid underestimation of localized mRNA in the lines with a high transcriptional signal compared with the others. It needs to be taken into account that this type of calculation determines an overestimation of localized mRNA in all the lines analyzed (e.g. Fig. S7B). The mRNA counts for each pharynx from transverse views in maximum intensity projections (Fig. 2D) were obtained by drawing an ‘apical’ outline along the apical side of all the pharyngeal cells marked by cr.DLG-1::GFP, and a ‘total’ outline boxing the whole body of the pharyngeal cells taken into account with the ‘apical’ outline. The amount of apically localized mRNA for pharynxes has been calculated as ‘apical’ mRNA divided by ‘total’ mRNA. Statistical analyses were performed in the software R (R Core Team, 2021; https://www.R-project.org/) and dot plots with box plots were generated with the ggplot2 package (Wickham, 2016; https://ggplot2.tidyverse.org). Each graph possesses a thick black line within the box that represents the median, two hinges for the first and third quartiles, two whiskers that define the upper and lower limits, and dots represent individual results. Red dots represent the mean. A *t*-test (one or two tails: see each figure legends for details) was employed to test the statistical differences between the conditions analyzed.

### Intensity profile analyses

Apicobasal and apical intensity profile analyses were performed extracting the data for signal intensities from the Multi Plot tool from ROI Manager of ImageJ (https://imagej.net/). Regions of interests (ROIs) were chosen as it has been schematically described in Fig. 6A,B. Signal intensities were acquired for *dlg-1::gfp* mRNA (channel 1), AJM-1 (channel 2), DLG-1::GFP (channel 3) and DNA (channel 4). To avoid the quantification of nuclear mRNA signal, which is not relevant for our analyses, we removed from the *dlg-1::gfp* channel the signal overlapping the DNA staining (Fig. S9).

### Protein sequence analysis

To identify putative signal peptide sequences in amino acid sequences of selected proteins, we took advantage of the SignalIP-5.0 Server (Center of Biological Sequence Analysis – CBS). Nucleotide or amino acid sequences analyses were performed with Expasy (Swiss Institute of Bioinformatic – SIB) or Clustal Omega (European Molecular Biology Laboratory-European Bioinformatic Institute – EMBL-EBI).

## Supplementary Material

Supplementary information

Reviewer comments
