# Supplementary material for: Translation-dependent mRNA localization to Caenorhabditis elegans adherens junctions
Source: Development. 2021 Dec 16;148(24):dev200027. doi: 10.1242/dev.200027 (PMC8722394; doi:10.1242/dev.200027)
Supplement: Supplementary information [file develop-148-200027-s1.pdf]

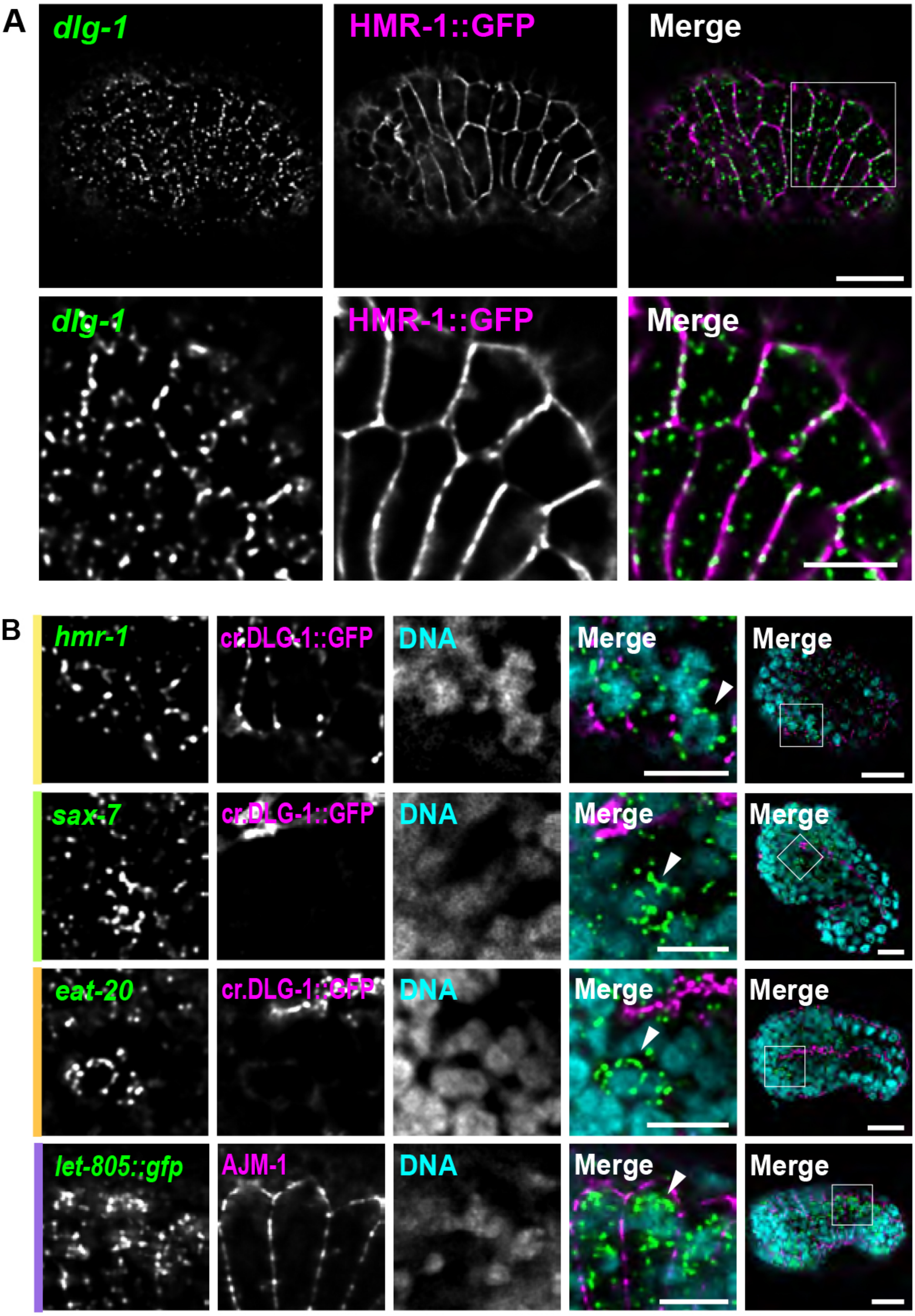
A
dlg-1
HMR-1::GFP
Merge
dlg-1
HMR-1::GFP
Merge
B
hmr-1
cr.DLG-1::GFP
DNA
Merge
Merge
sax-7
cr.DLG-1::GFP
DNA
Merge
Merge
eat-20
cr.DLG-1::GFP
DNA
Merge
Merge
let-805::gfp
AJM-1
DNA
Merge
Merge

Fig. S1. Endogenous *dlg-1* is enriched at the *Ce*AJ; transmembrane protein-coding mRNAs show instances of perinuclear localization. A. Fluorescent micrographs of the entire C. elegans embryos (upper panels) and zoom-ins (lower panels) showing smFISH signal of endogenous dlg-1 mRNAs (green) in epidermal and seam cells of a bean stage, fluorescent signal of the transgenic GFP-tagged HMR-1 protein (HMR-1::GFP, magenta), and merges. Endogenous/non-tagged dlg-1 mRNA shows CeAJ/membrane localization like its endogenous/GFP CRISPR-tagged version. Scale bar (upper panels): 10 µm. Scale bars (lower panels): 5 µm. **B.** Fluorescent micrographs of portions and entire C. elegans embryos showing instances of perinuclearly localized mRNAs coding for transmembrane proteins (HMR-1, SAX-7, EAT-20, and LET-805::GFP). smFISH signal of localized mRNAs hmr-1, sax-7, eat-20, and let-805::gfp (green), fluorescent signal of the CRISPR-engineered DLG-1::GFP (cr.DLG-1::GFP) or AJM-1 (magenta), and merges. To the left of each image, bars color-coded as in Fig. 1A to indicate to which sub-class of factors the mRNAs code for. Arrowheads: examples of cells with perinuclearly localized mRNAs. Scale bars: 5 µm.

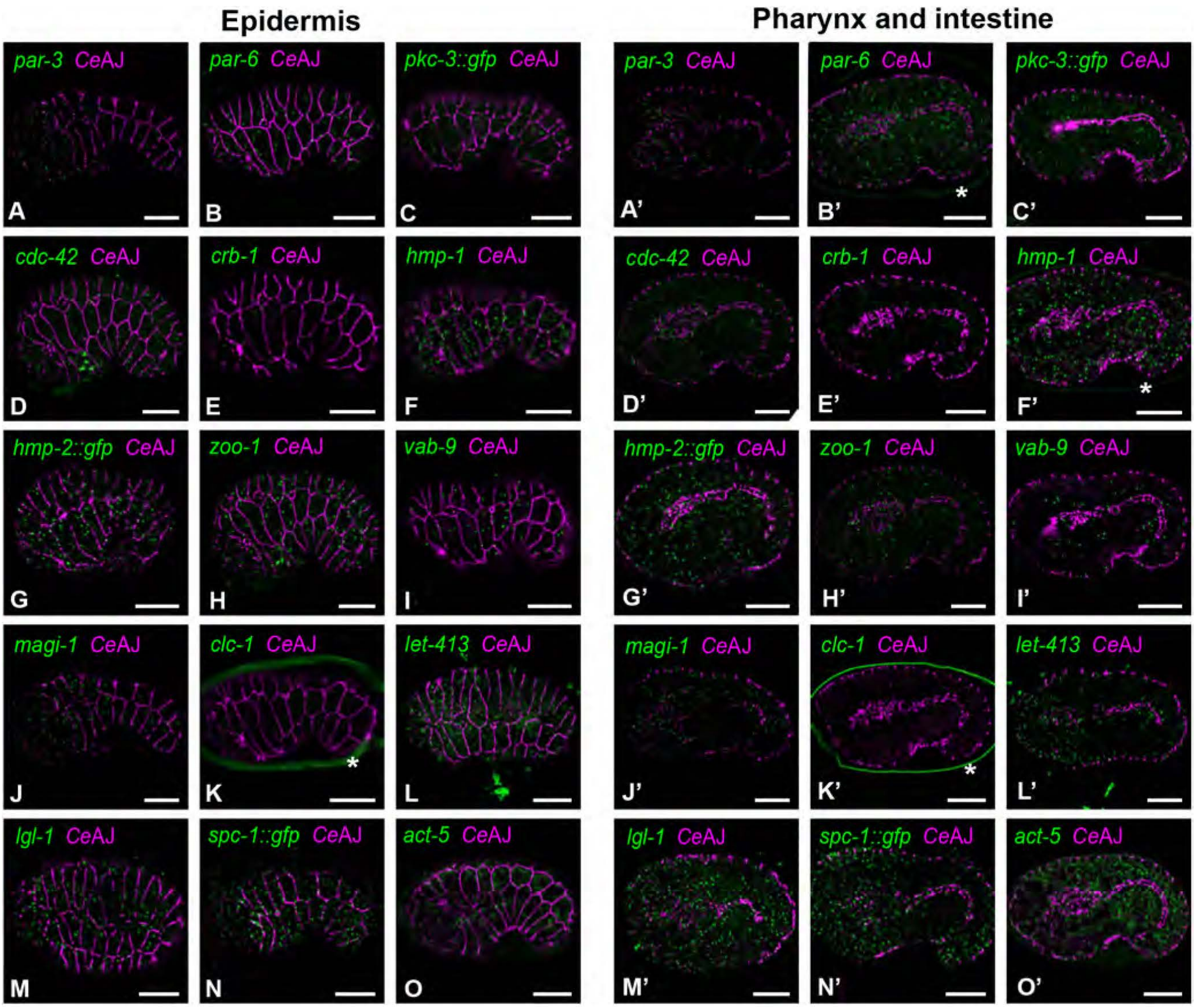

Fig. S2. Fifteen mRNAs coding for *Ce*AJ, cell polarity, and related components do not show any evident subcellular enrichment. A-O. Fluorescent micrographs of entire *C. elegans* embryos at the bean/comma stages focusing on the epidermis for the stated mRNAs in each image (green). Magenta: *Ce*AJ marker (cr.DLG-1::GFP or AJM-1 for GFP-tagged lines). White asterisks mark unspecific signal from eggshells. Scale bars: 10 µm. **A'-O'.** Same as in A-O but for the forming digestive track (pharynx and intestine).

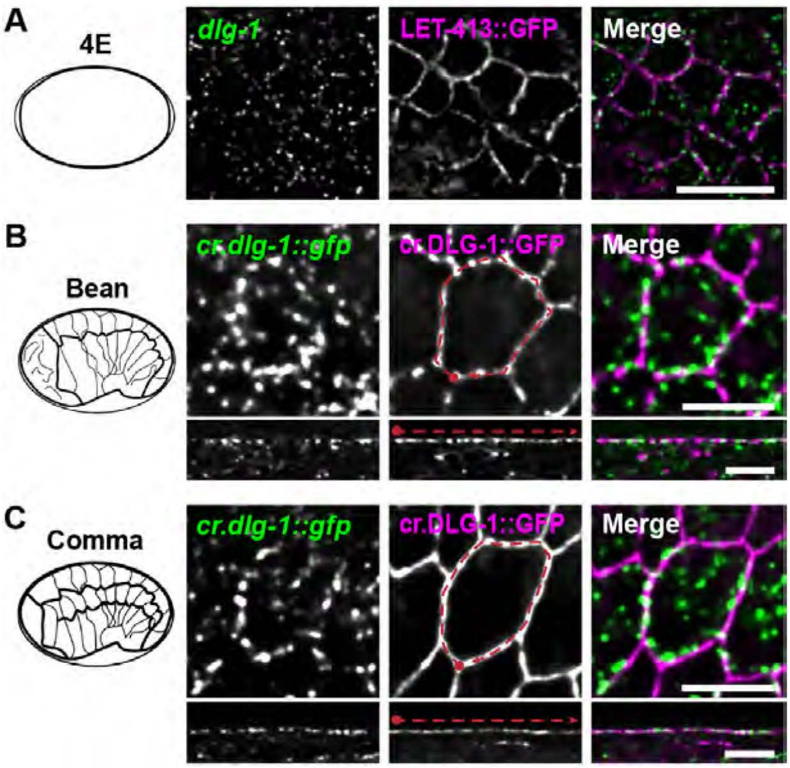

Fig. S3. *dlg-1* mRNA is enriched at the cell membrane prior to *Ce*AJ maturation. A. Fluorescent micrographs of developing epithelial cells of a portion of a *C. elegans* embryo at the 4E stage (schematics on the left) showing smFISH signal of the endogenous *dlg- 1* mRNA (green), fluorescent signal of the transgenic GFP-tagged LET-413 protein (LET- 413::GFP, magenta), and merges. Scale bars: 10 µm. **B-C.** Top views of fluorescent micrographs of a seam cell at the embryonic bean and comma stages (schematics on the left). Images show smFISH signal of the *dlg-1::gfp* mRNA (*cr.dlg-1::gfp*, green), fluorescent signal of the CRISPR-engineered GFP-tagged DLG-1 protein (cr.DLG-1::GFP, magenta), and merges. Red asterisks mark the site from which transverse projections shown below start from. Scale bar: 5 µm. Below: corresponding transverse sections on a flat plane of the sole rolled out membrane of the seam cell shown above. Red asterisks mark the horizontal location of *Ce*AJ and the starting site shown above. Scale bar: 5 µm.

Development • Supplementary information

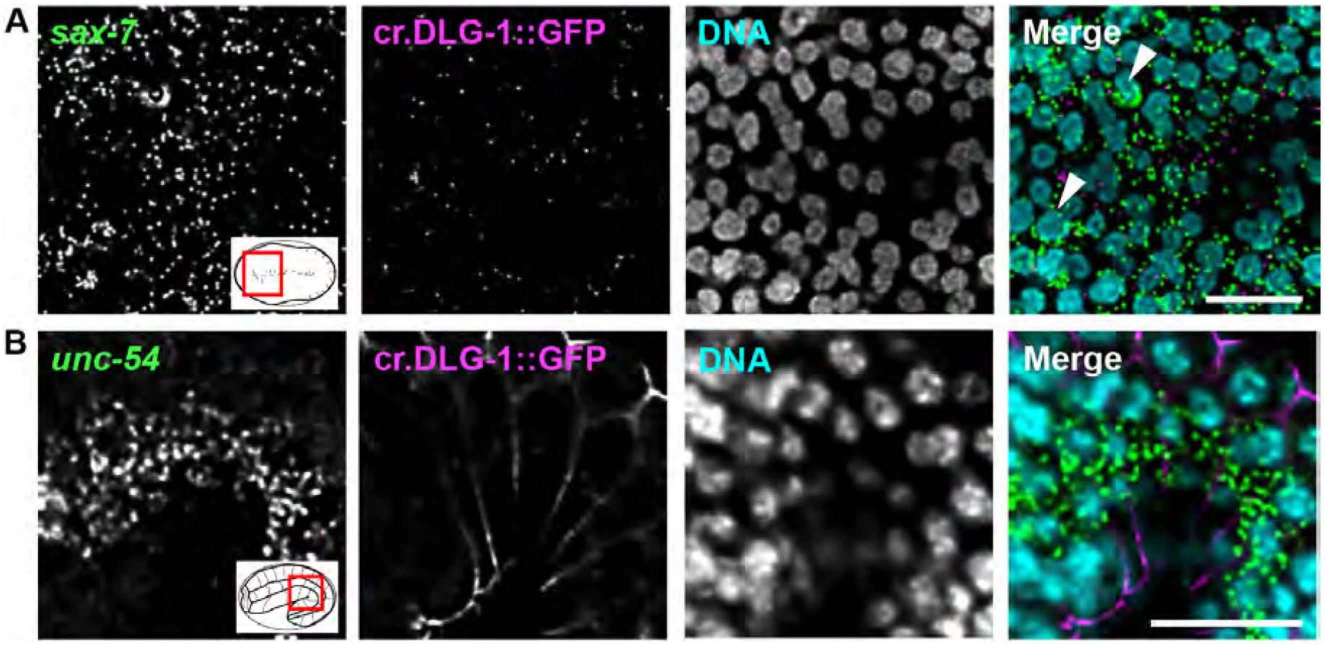

Fig. S4. The UTRs enrolled in transgenic lines derive from unlocalized mRNAs. A-B. Maximum intensity projections of 5 (A) and 3 (B) Z-stacks of fluorescent micrographs of portions (red box in the cartoon) of *C. elegans* embryos at the 16E (A) and 1.5-fold (B) stages showing smFISH signal of *sax-7* (A) and *unc-54* (B) mRNAs (green), fluorescent signal of the endogenous GFP-tagged DLG-1 protein (cr.DLG-1::GFP, magenta), DNA (cyan), and merges. Arrowheads: examples of cells with perinuclearly localized *sax-7* mRNA. Such a localization depends on the signal peptide located in the CDS of *sax-7* and not on its UTRs. Scale bars: 10 µm.

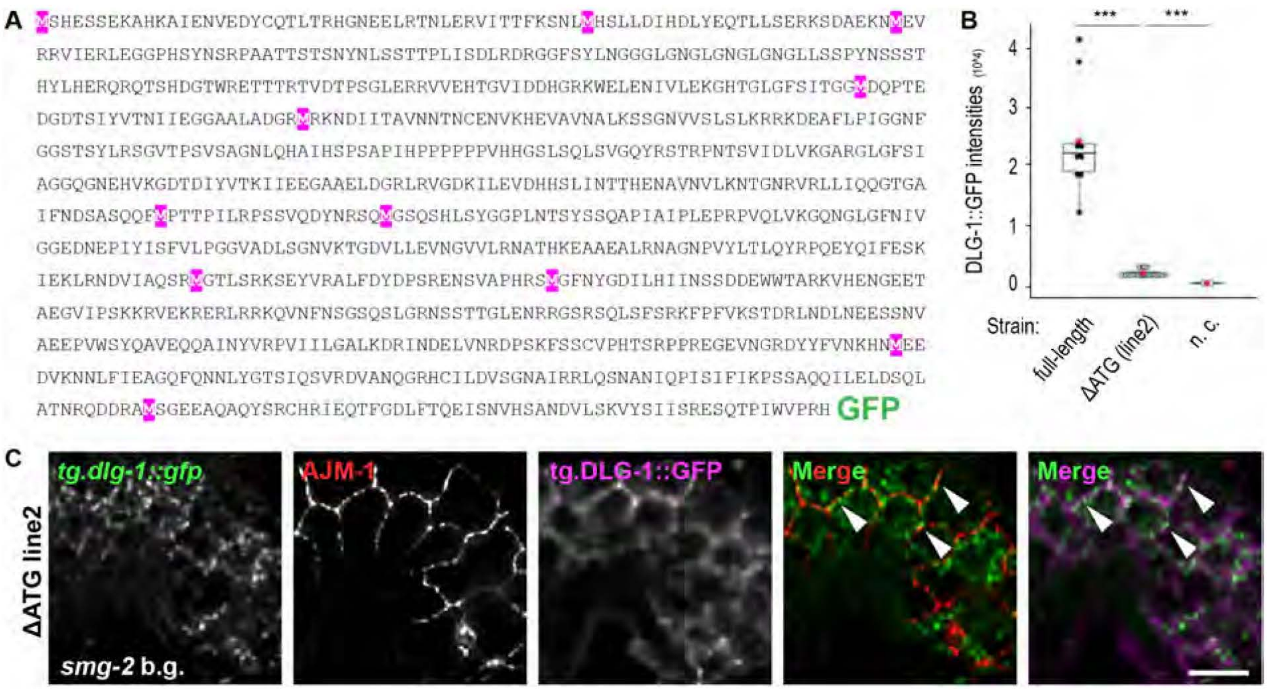

Fig. S5. *dlg-1* mRNA coding sequence possesses putative alternative start codons that may allow its partial translation. A. Transgenic GFP-tagged (green "GFP" at the carboxy-terminus) DLG-1 protein sequence. Highlighted in magenta are methionine amino acids corresponding to the green asterisks show in Fig. 4A. **B.** Dot plot with box plot: each dot represents the GFP intensity of pharynxes of adult worms carrying a "full-length" (n = 9; mean = 24.6 x 10^3; StDev = 9.4 x 10^3) or a "ΔATG line2" transgene (n = 15; mean = 2.0 x 10^3; StDev = 0.1 x 10^3). GFP intensities from animals of the "ΔATG line2" not carrying a transgene (recognizable through the "rolling" phenotype from the co-marker *rol-6*) were used as a negative control ("n. c.") (n = 3; mean = 0.4 x 10^3; StDev = 0.1 x 10^3). Red dots represent the mean. Significance of statistical analyses (*t*-test, one tail): *** = < 0.001. **C.** Fluorescent micrographs of a lateral portion of seam and epithelial cells at the late bean stage of a *C. elegans* embryo showing an example of a "ΔATG" transgene expressed in a null mutant background for an NMD component ("*smg-2* b.g.", bottom)., that is able to express its coded protein partially ("ΔATG line2"). As in Fig. 4C-E: smFISH signal of altered ATG ("ΔATG") *tg.dlg-1::gfp* mRNAs (green), immuno-fluorescent signal of the endogenous AJM-1 protein (red), fluorescent signal of the corresponding ΔATG tg.DLG-1::GFP protein (magenta), and merges. Arrowheads: examples of laterally localized mRNAs. Scale bar: 5 µm.

Development • Supplementary information

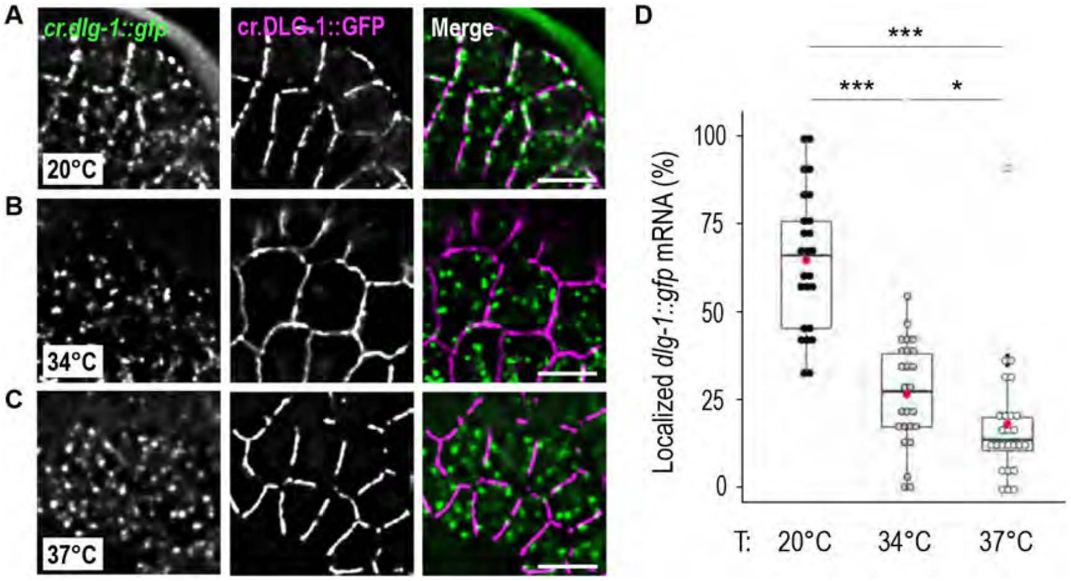

Fig. S6. *dlg-1* mRNA localization is lost upon heat stress. A-C. Fluorescent micrographs of seam cells of *C. elegans* embryos at the late bean stage that have been grown at 20°C (A) or subsequently subjected to a 1-hour heat-shock at 34°C (B) or 37°C (C) on plates. The images show smFISH signal of *cr.dlg-1::gfp* mRNA (green), fluorescent signal of the corresponding cr.DLG-1::GFP (magenta), and merges. Scale bars: 5 µm. **D.** Dot plot with box plot: each dot represents the percentage of laterally localized versus total cellular *cr.dlg-1::gfp* mRNA in each seam cell analyzed for embryos grown at 20°C (black; mean = 65.36; StDev = 19.78) or subsequently subjected to 34°C (grey; mean = 27.35; StDev = 14.85) or 37°C (white; mean = 18.70; StDev = 18.55) heat-shock (n = 25 for all conditions). Data derived from 5 different embryos. Significance of statistical analyses (*t*-test, one tail): * = < 0.05; *** = < 0.001.

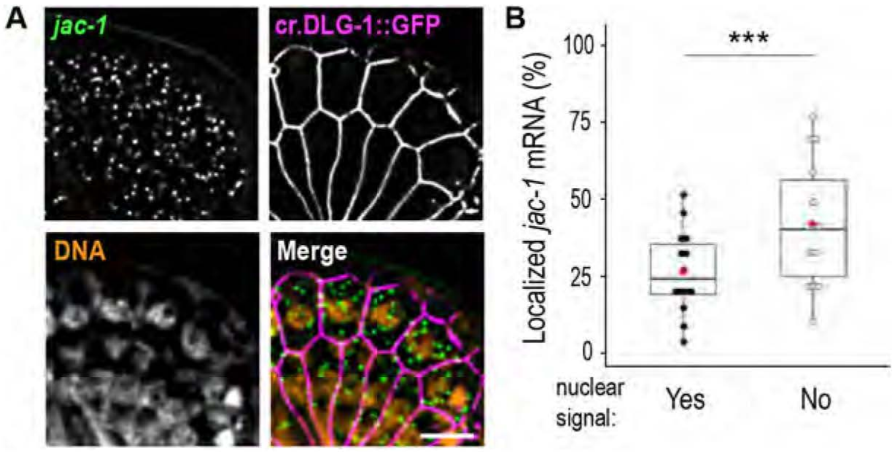

Fig. S7. The baseline percentage of lateral localization to call an mRNA unlocalized varies if the nuclear signal is taken under consideration or not. A. Fluorescent micrographs of the five posterior seam cells of a *C. elegans* embryo at the bean stage. The images show smFISH signal of *jac-1* mRNA (green), fluorescent signal of cr.DLG-1::GFP (magenta), DNA staining (orange), and merge. Scale bar: 5 µm. **B.** Dot plot with box plot: each dot represents the percentage of laterally localized versus "total" ("nuclear signal: yes"; black; mean = 25.87; StDev = 13.36) or versus "total" minus "nuclear" ("nuclear signal: no"; white; mean = 40.98; StDev = 20.05) *jac-1* mRNA in each seam cell at the bean stage analyzed (n = 14) (Table S2; Materials and methods). Data derived from 3 different embryos. Significance of statistical analyses (*t*-test, one tail): *** = < 0.001.

Development • Supplementary information

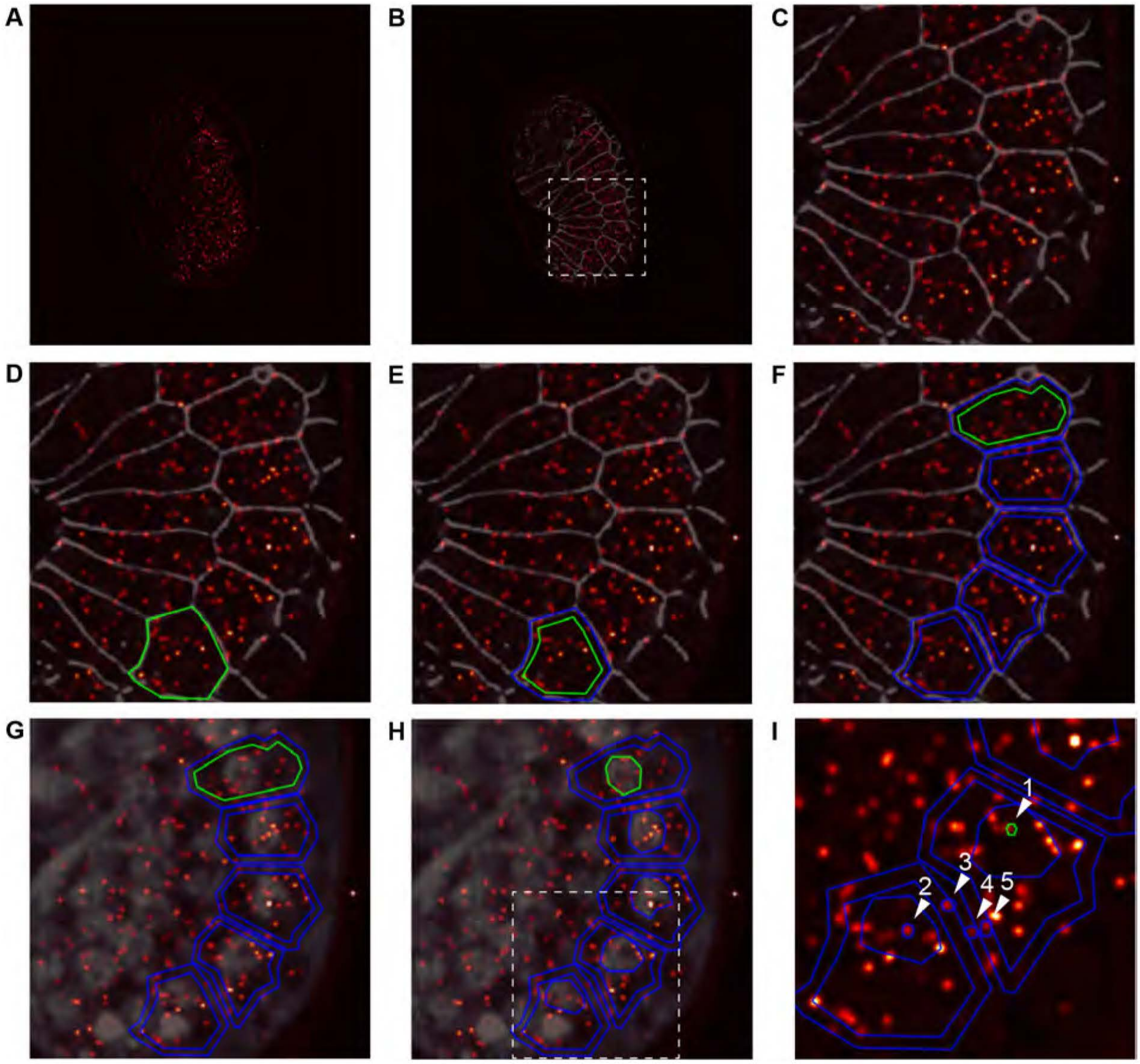

Fig. S8. Quantitation through FISH-quant provides total, cytoplasmic, and nuclear mRNA counts. A. Fluorescent micrograph (maximum intensity projection) of the same embryo shown in Fig. S7A showing smFISH signal of *jac-1* mRNA (red) from the FISH-quant platform. **B.** Same embryo as in (A) with cr.DLG-1::GFP signal (grey) to identify seam cells. Dashed square: portion of the embryo zoomed-in in (C). **C.** Zoom-in showing the five seam cells that will be undergoing the analysis through FISH-quant. **D.** Example of an outline (green) drawn along the border of a seam cell marked by cr.DLG-1::GFP signal. This outline provides the counts for the "total" cellular mRNA. **E.** Example of an outline (green) drawn parallel (± 500 nm) to the outer border (blue) of the same seam cell. This outline provides the counts for the "cytoplasmic + nuclear" mRNA. **F.** Outlines of the five seam cells that will be undergoing the analysis through FISH-quant. **G.** Same as (F), but showing the DNA signal (grey) instead of the cr.DLG-1::GFP signal. **H.** Same as in (G) showing the outlined DNA signals (small polygons in blue and green) to mark the "nuclear" mRNA. Dashed square: portion of the embryo zoomed-in in (I). **I.** Zoom-in on two of the five seam cells that will be undergoing the analysis through FISH-quant and showing the outlined mRNA dots chosen as units (small ellipses in blue and green).

Development • Supplementary information

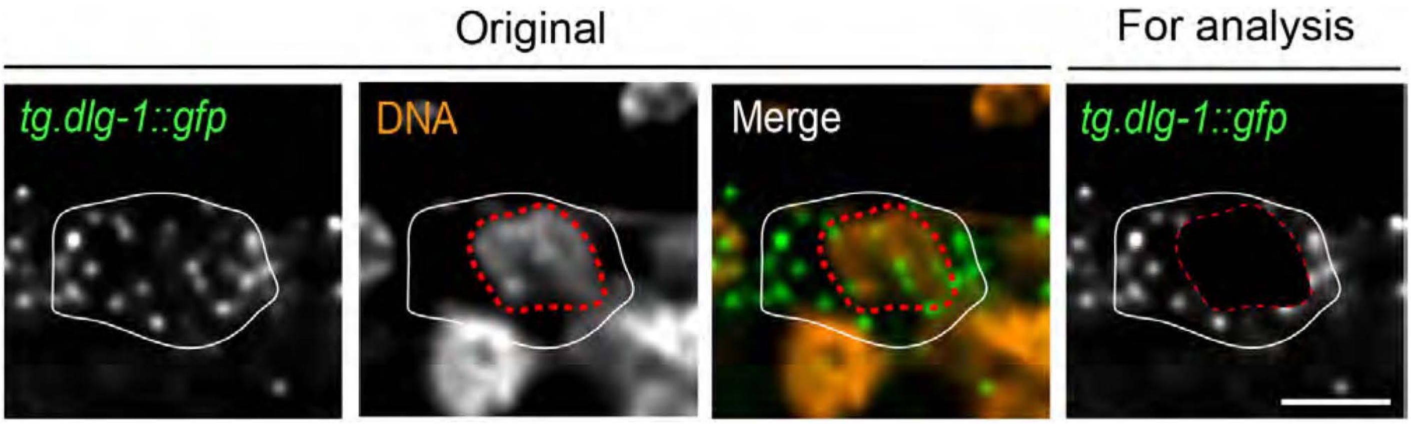

Fig. S9. Removal of smFISH signal overlapping with DNA in apicobasal and apical profile analyses. Fluorescent micrographs of a seam cell at the bean stage seen in frontal view (ΔL27 line). In the first three panels, from left to right: smFISH signal of *tg.dlg-1*$^{\Delta L27}$ mRNA (green), DNA staining (orange), and merge. White continuous lines: periphery of the cell. Red dashed lines: periphery of the DNA staining. On the very right: smFISH signal of *tg.dlg-1*$^{\Delta L27}$ mRNA, (same as on the very left) with the mRNA signal overlapping with the DNA staining being removed for profile analyses shown in Fig. 6 (Methods). Scale bar: 2.5 μm.

Table S1. List of factors enrolled in the smFISH survey. Names of the factors and their orthologues whose mRNAs were tested in our smFISH-based survey for localized mRNAs highlighted in the same color-code as in Fig. 1A to address to which group of localized protein they belong to (Factor); to what (if any) AS they belong to (Adhesion system); subcellular localization of the stated factor (Protein localization); WormBase identification number (WormBaseID); gene sequence name (Sequence name); mRNA localization observed in this study (Observed mRNA localization); target of smFISH probes (smFISH probes; for full list of probe sequences, see Table S4); strain that has been tested in our survey for the corresponding mRNA/factor (Strain tested); reference for the corresponding factor (Reference).

Click here to download Table S1

Table S2. Raw data for image quantitation. Raw data (mRNA counts), percentages of localization, means, and standard deviations, for all the quantification analyses in this study.

Click here to download Table S2

Table S3. List of methionine amino acid within the DLG-1 sequence. Amino acid positions and domain location of the 11 methionine amino acids found in the DLG-1 sequence, corresponding to the green asterisks in Fig. 4A.

Click here to download Table S3

Table S4. List of *C. elegans* strains. Detailed list of names, genotypes, and references of C. elegans strains used in this work.

Click here to download Table S4

Table S5. List of smFISH probes. Sequences of the smFISH primary probes used in this study.

Click here to download Table S5

Development • Supplementary information

Supplemental references

Achilleos, A., Wehman, A. M., & Nance, J. (2010). PAR-3 mediates the initial clustering and apical localization of junction and polarity proteins during C. elegans intestinal epithelial cell polarization. Development, 137(11), 1833-42. https://doi:10.1242/dev.047647.

Asano, A., Asano, K., Sasaki, H., Furuse, M., & Tsukita, S. (2003). Claudins in Caenorhabditis elegans: their distribution and barrier function in the epithelium. Curr Biol, 13(12), 1042-6. https://doi.org/10.1016/s0960-9822(03)00395-6

Carberry, K., Wiesenfahrt, T., Geisler, F., Stöcker, S., Gerhardus, H., Überbach, D., Davis, W., Jorgensen, E., Leube, R. E., & Bossinger, O. (2012). The novel intestinal filament organizer IFO-1 contributes to epithelial integrity in concert with ERM-1 and DLG-1. Development, 139(10), 1851-62. https://doi.org/10.1242/dev.075788

Croce, A., Cassata, G., Disanza, A., Gagliani, M. C., Tacchetti, C., Malabarba, M. G., Carlier, M. F., Scita, G., Baumeister, R., & Di Fiore, P. P. (2004). A novel actin barbed-end-capping activity in EPS-8 regulates apical morphogenesis in intestinal cells of Caenorhabditis elegans. Nat Cell Biol, 6(12), 1173-9. https://doi.org/10.1038/ncb1198

Jia, R., Li, D., Li., M., Chai, Y., Liu, Y., Xie, Z., Shao, W., Xie, C., Li, L., Huang, X., Chen, L., Li, W., & Ou, G. (2019). Spectrin-based membrane skeleton supports ciliogenesis. PLoS Biol, 17(7), e3000369. https://doi:10.1371/journal.pbio.3000369.

Marston, D. J., Higgins, C. D., Peters, K. A., Cupp, T. D., Dickinson, D. J., Pani, A. M., Moore, R. P., Cox, A. H., Kiehart, D. P., & Goldstein, B. (2016). MRCK-1 Drives Apical Constriction in C. elegans by Linking Developmental Patterning to Force Generation. Curr Biol, 26(16), 2079-89. https://doi:10.1016/j.cub.2016.06.010.

Morrissey, M. A., Keeley, D. P., Hagedorn, E. J., McClatchey, S. T. H., Chi, Q., Hall, D. H., & Sherwood, D. R. (2014). B-LINK: a hemicentin, plakin, and integrin-dependent adhesion system that links tissues by connecting adjacent basement membranes. Dev Cell, 31(3), 319-331. https://doi.org/10.1016/j.devcel.2014.08.024

Norman, K. R., & Moerman, D. G. (2002). Alpha spectrin is essential for morphogenesis and body wall muscle formation in Caenorhabditis elegans. J Cell Biol, 157(4), 665-77. https://doi.org/10.1083/jcb.200111051

Page, M. F., Carr, B., Anders, K. R., Grimson, A., & Anderson, P. (1999) SMG-2 is a phosphorylated protein required for mRNA surveillance in Caenorhabditis elegans and related to Upf1p of yeast. Mol Cell Biol, 19(9), 5943-51. https://doi:10.1128/mcb.19.9.5943.

Quintin, S., Wang, S., Pontabry, J., Bender, A., Robin, F., Hyenne, V., Landmann, F., Gally, C., Oegema, K., & Labouesse, M. (2016). Non-centrosomal epidermal microtubules act in parallel to LET-502/ROCK to promote C. elegans elongation. Development, 143(1), 160-73. https://doi.10.1242/dev.126615.

Suman, S. K., Daday, C., Ferraro, T., Vuong-Brender, T., Tak, S., Quintin, S., Robin, F., Gräter, F., & Labouesse, M. (2019). The plakin domain of C. elegans VAB-10/plectin acts as a hub in a mechanotransduction pathway to promote morphogenesis. Development, 146(24), dev183780. https://doi.org/10.1242/dev.183780

Yang, Y., Zhang, Y., Li, W. J., Jiang, Y., Zhu, Z., Hu, H., Li, W., Wu, J. W., Wang, Z. X., Dong, M. Q., Huang, S., & Ou, G. (2017). Spectraplakin Induces Positive Feedback between Fusogens and the Actin Cytoskeleton to Promote Cell-Cell Fusion. Dev Cell, 41(1), 107-120. https://doi:10.1016/j.devcel.2017.03.006.

Development • Supplementary information
