# Supplementary material for: Translation-dependent mRNA localization to Caenorhabditis elegans adherens junctions
Source: Development. 2021 Dec 16;148(24):dev200027. doi: 10.1242/dev.200027 (PMC8722394; doi:10.1242/dev.200027)
Supplement: Reviewer comments [file develop-148-200027_review_history.pdf]

Development

Review COMMONS

Translation-dependent mRNA localization to *Caenorhabditis elegans* adherens junctions

Cristina Tocchini, Michèle Rohner, Laurent Guerard, Poulomi Ray, Stephen E. Von Stetina and Susan E. Mango
DOI: 10.1242/dev.200027

Editor: Swathi Arur

Review timeline

Submission to Review Commons:	25 May 2021
Submission to Journal of Cell Science:	16 July 2021
Editorial decision:	20 July 2021
First revision received:	12 October 2021
Editorial decision:	1 November 2021
Second revision received:	5 November 2021
Accepted:	8 November 2021

Reviewer 1

Evidence, reproducibility and clarity

Summary

In the current study Tocchini et al analyze mRNA localization during development of Caenorhabditis elegans embryonic epithelia. Using smFISH-based method they have identified mRNAs associated with the cell membrane or cortex, and with apical junctions. They showed that most of mRNAs involved in AS-II cell adhesion system localize to the membrane. To examine how epithelial morphogenesis affects mRNA localization, authors studied two transcripts encoding DLG-1 and AJM-1 that form a complex. Data showed that studied mRNAs enrichment at the CeAJ varies at distinct stages and cell types of embryogenesis. Then the study was focused on one of the identified transcripts - dlg-1/discs large. Using transgenic lines authors demonstrated that dlg-1 localization to the CeAJ is UTRs-independent, but requires active translation. Moreover, authors mapped protein domains involved in that process.

Major comments:

Fig. 1: Main and supplementary figures present smFISH signals for eight localized mRNAs, while in the results section authors describe that they analyzed twenty-five transcripts. Authors should explain the choice of transcripts presented in the paper. Moreover, smFISH signal of different localized mRNAs in epidermal cells was visualized at different stages (bean, comma or late comma), and authors did not comment what was the reason of such conditions. This may make transcripts localization results difficult to interpret, as further analysis showed that mRNA localization varied in a stage-specific manner. Did author used smFISH probes designed against endogenous mRNAs for all tested transcripts? Marking dlg-1 mRNA as dlg-1-gfp suggests that smFISH probe was specific for gfp transcript. Is it true? If yes, authors should compare localization of wild-type endogenous dlg-1 mRNA with that of the transcript encoding a fusion protein, to confirm that fusion does not affect mRNA localization.

Fig. 2B: Authors conclude that at later stages of pharyngeal morphogenesis mRNA enrichment at the CeAJ decreased gradually in comparison to comma stage. Data do not show statistically significant decrease in ratio of localized mRNAs - for dlg-1: bean: 0.39{plus minus}0.09, comma:

© 2021. Published by The Company of Biologists under the terms of the Creative Commons Attribution License (https://creativecommons.org/licenses/by/4.0/).

0.29{plus minus}0.08, 1.5-fold: 0.30{plus minus}0.09; for ajm-1: bean: 0.36{plus minus}0.08, comma: 0.30{plus minus}0.05, 1.5-fold: 0.28{plus minus}0.09.

Fig. 4: What was the difference between the first and the second ΔATG transgenic line? Authors should analyze the size of the truncated DLG-1 protein that is expressed from the second ΔATG transgenic line that localizes to CeAJ. Knowing alternative ATGs and protein size may suggest domain composition of the truncated protein. This will allow to confront truncated protein localization with the results from Fig. 5. Moreover, to prove that the localization of dlg-1 mRNA at the CeAJ is translation-dependent, additional experiment should be performed where transcripts localization will be analyzed in embryos treated with translation inhibitors such as cycloheximide (translation elongation inhibitor) and puromycin (that induces premature termination).

Minor comments:

In the introduction section authors should emphasize the main goal and scientific significance of the paper.
Fig 1A: It's hard to distinguish different colors on the schematics. Schematics presents intermediate filaments that are not included in the Table 1.

Fig. 1C: dlg-1 transcript is marked as dlg-1-gfp on the left panel and dlg-1 on the right panel.

Fig. 2B: Axis labels and titles are not visible, larger font size should be used.

Fig. 5C: Enlarge the font size.

Fig. S2: Embryonic stages should be marked on the figure for easier interpretation.

Significance

This study provides a few contributions into understanding mRNA localization in Caenorhabditis elegans during embryo development. Firstly, it identifies adhesion system II mRNAs associated with epithelial cells. Secondly, it demonstrates a case study of translation-dependent dlg-1/DLG-1 mRNA localization mechanism that does not involve zip codes. Finally, it provides a model showing the roles of different DLG-1 domains in dlg-1 localization. The results are compelling and experiments are well presented, although in my opinion authors should provide a stronger evidence to support the idea that active translation is essential for dlg-1 localization.

Overall, I believe the work will have a wide appeal covering areas such as mRNA localization, developmental biology and embryogenesis.

My field of expertise is in the RNA-protein interactions and mRNA turnover using biochemical methods as well as in vivo studies in C. elegans and mammalian cell lines. I do not have an expertise in smFISH-based methods.

Reviewer 2

Evidence, reproducibility and clarity

Summary:

Tocchini et al. screened apical junction and cell membrane proteins for mRNA localization. They identified multiple proteins that are translated from localized mRNAs. Of these, dlg-1 (Discs large) mRNA localizes to cell cortices of dorsal epithelial cells, endoderm cells, and epidermal (seam) cells and is dependent on active translation for transport. The manuscript dissects the contributions of different DLG-1 protein domains to mRNA localization.

A major strength of the paper is the way it assesses translational-dependence in a transcript-specific way without perturbing translation globally. The authors cleverly combine mutations in ATG start sites with a knock down of the non-sense mediated decay pathway. This allows Tocchini

© 2021. Published by The Company of Biologists under the terms of the Creative Commons Attribution License (https://creativecommons.org/licenses/by/4.0/).

et al to examine whether dlg-1 mRNA depends on active translation for localization, which it does. The authors observe an interesting finding, that the domains required for protein localization can be separated from those required for mRNA localization. Namely, mRNA localization (but not protein localization) requires C-terminal domains of the protein.

My major points of concern focus on the presentation and interpretation of Figure 5. In this figure, the blocking approach used seems confounding, the observations described by the authors are not visible, the quantification is confusing, and the interpretations seem like an over-reach.

Major comments:

- Figure 2 requires a negative (or uniformly distributed) mRNA control for comparison. Figure 2C should be quantified. The plot quality should be improved, and appropriate statistical tests should be employed to strengthen the claimed findings.

- Most claims of perinuclear mRNA localization are difficult to see and not well supported visually or statistically. The usage of DAPI markers, membrane markers, 3D rendering, or a quantified metric would bolster this claim. Also, sax-7 is claimed to be perinuclear and elsewhere claimed to be uniform then used as a uniform control. Please explain or resolve these discrepancies more clearly.

- The major concern about the paper is the data display and interpretation of Figure 5C. I'm not comfortable with the approach the authors took of blurring out the nucleus. A more faithful practice would be to use an automated mask over DAPI staining or to quantify the entirety of the cell. If the entirety of the cell were quantified, one could still focus analysis on specific regions of relevance. The interpretations distinguishing membrane versus cytoplasmic localization (or mislocalization) are hard to differentiate in these images especially since they are lacking a membrane marker. The ability to make these distinctions forms the basis of Tocchini et al's two pathways of dlg-1 mRNA localization. These interpretations also heavily rely on how the image was processed through the different Z- stacks, and it's not clear to me how that was done. For example, the diffusion of mRNA in figure 5F and 5I are indistinguishable to my eye but are claimed to be different.

- To my eye, it seems that Figure 5 could be more faithfully interpreted to state that DGL-1 protein localization depends on the L27-SH3 domains. The Huk/Guk domains are dispensable for DLG-1 protein localization; however, through other studies, we know they are important for viability. In contrast, dlg-1 mRNA localization requires all domains of the protein (L27-Guk). It is exceptionally interesting to find a mutant condition in which the mRNA and protein localizations are uncoupled. It would be very interesting to explore in the discussion or by other means what the purpose of localized translation may be. Because, in this instance, proper mRNA localization and protein function are closely associated, it may suggest that DLG-1 needs to be translated locally to function properly.

- The manuscript requires an improve materials & methods description of the quantification procedures and statistics employed.

Minor & Major comments together:

Text

- Summary statement: Is "adherent junction" supposed to be "adherens junction?"

- Abstract: Sentence 1, I think they should add a caveat word to this sentence. Something like "...phenomenon that can facilitate sub-cellular protein targeting." In most instances this isn't very well characterized or known.

- In the first paragraph, it might be good to mention that Moor et al also showed that mRNA localize to different regions to alter their level of translation (to concentrate them in high ribosome dense regions of the cell).

© 2021. Published by The Company of Biologists under the terms of the Creative Commons Attribution License (https://creativecommons.org/licenses/by/4.0/).

- There are some new studies of translation-dependent mRNA localization - that might be good to highlight - Li et al., Cell Reports (PMID: 33951426) 2021; Sepulveda et al., 2018 (PCM), Hirashima et al., 2018; Safieddine, et al 2021. Also, Hughes and Simmonds, 2019 reviews membrane associated mRNA localization in Drosophila. And a new review by Das et al (Nat Rev MCB) 2021 is also nice.

Parker et al. did not show that the 3'UTR was dispensable for mRNA localization. They showed the 3'UTR was sufficient for mRNA localization.

- In the second paragraph, the sentence about bean stages is missing one closing parenthesis.

- Last paragraph: FISH is fluorescence, not fluorescent.

- Both "subcellular" and "sub-cellular" are used.

Minor comments - Figures

- Figure 1

  - Figure 1A is confusing. It's not totally clear what the rectangles and circles signify. There are many acronyms within the figure. Which of the cell types depicted in the figure are shown here? For example, for the dorsal cells, which is the apical v. basal side?
  - Some of the colors are difficult to distinguish, particularly when printed out or for red/green colorblind readers. Is erm-1 meant to be a cytoskeletal associated or a basolateral polarity factor?
  - The nomenclature for dlg-1 is inconsistent within "C".
  - Please specify what the "cr" is in "cr.dlg-1:-gfp" in the legend

- Figure 2

  - Can Figure 2C be quantified in a similar manner to 2A/2B?
  - 2B - please jitter the dots to better visualize them when they land on top of one another
  - Please include a negative control example, a transcript that is not peripherally localized for comparison.
  - There is no place in the text of the document where Fig 2C is referenced
  - I can't see any discernable ajm-1 localization in Fig 2A.
  - I can't see any dlg-1 pharangeal localization in Fig2C.
  - More details on how the quantification was performed would be welcome. Particularly, in 2B, what is the distance from the membrane in which transcripts were called as membrane-associated? What statistics were used to test differences between groups?

- Figure 3

  - Totally optional but might be nice: can you make a better attempt to approximate the scale of the cartoon depiction?
  - The GFP as an asterisk illustration may be confusing for some readers. Could you add another rectangular box to depict the gfp coding sequence?
  - This microscopy is beautiful!
  - Were introns removed? Is the endogenous copy still present?
  - The wording in the legend "CRISPR or transgenic" may be confusing as Cas9 genome editing is still a form of transgenesis.
  - The authors state that the 5'-3'UTR construct produces perinuclear dlg-1 transcripts but in the absence of DAPI imaging, it's not clear that this is the case.
  - Which probeset was used? The gfp probe?
  - Here, sax-7 is used as a uniform control, but sax-7 is claimed in Fig S1B-D as being perinuclear. This is a bit confusing.

- Figure 4

  - Excellent results! Really nice!
  - Fig 4A. The GFP depicted as a circle is strange.

© 2021. Published by The Company of Biologists under the terms of the Creative Commons Attribution License (https://creativecommons.org/licenses/by/4.0/).

- Fig 4A. Can you include the gene/protein name for easy skimming?
- Fig 4B. the color here is too faint and it is unclear what is being depicted. Overall, this part of the figure could be improved.
- Were the introns removed?

- Figure 5

- Fig 5A. can you add the gene/protein name
- Fig 5B. Can you you make the example apicobasal (non-apical) mRNA more distinctive? If it had its own peak in the lower trace, the reader would more clearly understand that this mRNA will be excluded from apical measurements whereas it will be included in apicobasal measurements.
- D' - I' The grey font is too light.
- D' - I' The inconsistent y-axis scaling makes it difficult to compare across these samples. Can you set them to the same maximum number?
- D' - I' The x-axis labels are formatted incorrectly
- The practice of masking out the nucleus appears to remove potentially important mRNAs that are not nuclear localized. This could really impact the findings and interpretation. Instead, consider an automated DAPI mask.
- I can't see what the authors are calling membrane diffuse versus cytoplasmic. This is making it hard for me to see their "two step" pathway to localization.
- "F" looks the same as "I" to me, but the authors claim they represent different patterns and use these differences as the basis for their claim that X.
- Can more details of the quantification be included? How were Z-sections selected, chosen for inclusion? Which Z-sections and how many were selected?
- Also, why do these measurements focus on what I think are the seam cells when Lockwood et al., 2008 show the entire epithelium that is much easier to see?
- Please name these constructs to correlate the text more explicitly to the figures.
- How many embryos were analyzed for each trace? How many embryos showed consistent patterns?
- Why were these cells used for study here? Lockwood et al., 2008 use a larger field of epithelial cells for visualization.

- Figure 6

- There are major discrepancies between what this figure is depicting graphically and what is described in the text. Again, I'm not comfortable making the "two step" claims this figure purports given the data shared in Figure 5.

Minor comments - Tables & Supplemental Figures

Table 1

- I think this table could be improved to more clearly illustrate which mRNAs were tested and what their mRNA localization patterns were (for example, gene name identifiers included, etc). Could the information that is depicted by gray shading instead be added as its own column? For example, have a column for "Observed mRNA localization"

- Can you add distinct column names for the two columns that are labeled as "protein localization - group"

- Can you also add which of these components are part of ASI v. ASII (as described in the introduction?

Supplemental Figure 1

- It is hard to see that some of these spots are perinuclear. More information (membrane marker, 3D rendering, improved metrics) is required to support this claim.

© 2021. Published by The Company of Biologists under the terms of the Creative Commons Attribution License (https://creativecommons.org/licenses/by/4.0/).

- What do these images look like over the entire embryo, not just in the zoomed in section?

- sax-7 localization in S4 looks similar but a different localization claim is made.

Supplemental Figure 2

- Before adherens junctions even exist dlg-1 go to the membrane - this is really neat!

Supplemental Figure 3

- Technical question: If either 5 or 3 stack images are used, how does this work? Do they have different z-spacings? Or do they do 5-stack images represent a wider Z-space?

Supplemental Figure 4

- Line #2 retains translation and keeps mRNA localization.

- Totally optional, but consider showing both lines in the main figure to illustrate the two possibilities.

- Materials and methods - how did they created the ATG mutations? Is it an array? - why does one translate, and one doesn't?

Significance

The authors discover that dlg-1, ajm-1, and hmr-1 mRNAs (among others) are locally translated, and this represents an important conceptual advance in the field as these are well studied proteins and important markers. This is the first study to illustrate translation- dependent mRNA localization in C. elegans, to my knowledge. The mechanisms transporting these mRNAs and their associated translational complexes to the membrane may represent a new pathway of mRNA transport and is therefore significant. The authors identify domains within DLG-1 responsible which is a nice advance. If they are unable to order the events of association as they claim in Figure 5 (and that I dispute), this doesn't detract from the impact of the paper.

Other high-profile studies have recently been published that echo how mRNA localization to membranes can be observed for transcripts that encode membrane-associated proteins (Choaib et al., Dev Cell, 2020; Li et al., Cell Reports, 2021 (PMID: 33951426); and Reviewed in Hughes & Simmonds, Front Gen, 2019). These recent findings underscore the impact of Tocchini et al.'s paper. Similar studies have identified mRNAs localizing through translation dependent mechanisms to a variety of different regions of the cell (Sepulveda et al., eLife, 2018; Hirashima et al., Sci Reports, 2018; Safieddine, et al., Nat Comm, 2021; and reviewed in Ryder et al., JCB 2020). Given the timely nature of these findings and the recent interest in these concepts, a broad readership of readers should be interested in this paper.

My field of expertise is in mRNA localization imaging and quantification. I feel sufficiently qualified to evaluate the manuscript on all its merits.

Reviewer 3

Evidence, reproducibility and clarity

Subcellular localization of mRNAs plays a critical role in gene regulation and ultimately cellular function. While mRNA untranslated regions often serve as key regulatory codes for expression, mRNA translation can also have a significant effect, a notable example being secretory peptides delivering translating transcripts to the endoplasmic reticulum. A complete understanding of the signals that organize mRNAs in the cell remains an open question. Here, Tocchini, et al. use the C. elegans embryo and single molecule FISH (smFISH) to determine the subcellular localization of key mRNAs involved in epithelial morphogenesis. This survey identifies several mRNAs that appear to localize to specific regions of the cell, such as the plasma membrane or apical junction, and in a

© 2021. Published by The Company of Biologists under the terms of the Creative Commons Attribution License (https://creativecommons.org/licenses/by/4.0/).

developmental stage-specific manner. Dissection of the mRNA of dlg-1/discs large, an apical junction component, provides evidence that mRNA localization requires active translation, but surprisingly the untranslated regions are dispensable. Further mRNA truncation mapping supports the model that the N-terminal coding region helps target mRNAs to the apical junctions, but the C-terminal coding regions are sufficient to localize dlg-1 mRNA to the plasma membrane. The manuscript describes a two-step model for dlg-1 localization and recruitment to the apical junction that depends on translation.

MAJOR:

1. The smFISH results are striking and implications exciting. The conclusions made from the smFISH results reported in all Figures will be strengthened considerably by quantifying the mRNA localized to the defined specific subcellular regions. At the very least, localization to the cytoplasm versus the plasma membrane should be determined as performed in Figure 2B, but quantifying finer localization will enhance the conclusions made about regional localization (e.g. CeAJ versus plasma membrane mRNA localization in Figure 5). Inclusion of a non-localizing control in Figures 1-4 will enable statistical comparisons between Mrna localizing and non-localizing groups.
2. The script used for smFISH quantitation should be included in the methods or published in an accessible forum (Github, etc). Criteria for mRNA "dot" calling should be defined in the methods. All raw smFISH counts should also be reported.
3. Figure 2: What is the localizing ratio of a non-localizing control mRNA (e.g. jac-1)? Including an unlocalized control with quantitation would strengthen the localization arguments presented.
4. Figure 5: Quantifying colocalization of mRNA and protein (+/- AFM-1) will strengthen the arguments made about mRNA/protein localization.
5. Discussion of the CeAJ mRNA localization mechanism is warranted. Do the authors speculate that the newly translated protein drives localization during translation, similar in concept to SRP-mediated localization to the ER, or ribosome association is a trigger to permit a secondary factor to drive mRNA localization, or another model?

MINOR:

- Please complete the following sentence: "We identified transcripts enriched at the CeAJ in a stage- and cell type-specific."
- It would be helpful to provide reference(s) for the protein localization summary in Table 1.
- Figure 2B: Did dlg-1 and ajm-1 localize at similar ratios? Appropriate statistics comparing the different ratios may be informative.
- Figure 2: In the paragraph that begins, "Morphogenesis of the digestive track," the text should refer to Figure 2C? If not, the text requires further clarification.
- Figure 2: Reporting the smFISH localizing ratios of 8E and 16E will be informative.
- Please include citations when summarizing the nonsense-mediated decay NMD mechanism and AJM-1 identifying the CeAJ.
- The sentence, "Embryos from our second ΔATG transgenic line displayed a little GFP protein and some dlg-1::gfp mRNA," should refer to Figure S4. An immunoblot of this reporter versus wild type may be informative regarding the approximate position of putative alternative start codon.
- Figure 5: N's and repetitions performed should be included for localization experiments.
- Please clarify that the "the mechanism of UTR-independent targeting is unknown in any species" refers to dlg-1 mRNA localization.
- "Our findings suggest..." discussion paragraph should reference Figure 6.

Significance

This well-written, well-cited manuscript describes the striking subcellular localization pattern of a critical, conserved gene involved in both animal development and human disease. The observation that the start codon, and thus translation, is necessary for transcript localization is a complete surprise, and opens exciting doors to investigate how translation leads to mRNA organization and its connection to tissue development. As such, this manuscript will be of broad interest to RNA, cell and developmental biologists, particularly those who investigate post-transcriptional gene regulation and protein complex assembly. However, while the images are indeed supportive of the

© 2021. Published by The Company of Biologists under the terms of the Creative Commons Attribution License (https://creativecommons.org/licenses/by/4.0/).

manuscript's claims, the conclusions will be markedly strengthened by quantifying the subcellular localization of mRNAs in the smFISH experiments, paired with negative controls (e.g. non-localizing, cytoplasmic mRNA). Addition of more quantitative smFISH analyses will enhance the experimental reproducibility, rigor, and statistical significance. The text, figures, and methods should also be revised to include more details about the smFISH analyses, in particular the inclusion of n's, descriptions of how spots were identified, descriptions of scripts used, and the raw mRNA counts.
Regardless, the reporter genes tested were well conceived and dlg-1 shows promise to be a fantastic model to further investigate the mechanisms underlying translation-dependent mRNA localization.

My expertise covers post-transcriptional gene regulation, the C. elegans model organism, and fluorescent imaging with smFISH.

Author response to reviewers' comments

1. General Statements

We thank the reviewers for their thoughtful comments. We were delighted the reviewers found our results "*compelling*", "*striking*", "*well presented*", "*implications exciting*", "*excellent results! really nice!*", "*this microscopy is beautiful*!" and "*translational-dependence* (of mRNA localization) *in a transcript-specific way without perturbing translation globally*", which is a "*complete surprise, and opens exciting doors to investigate how translation leads to mRNA organization and its connection to tissue development*" and "*may represent a new pathway of mRNA transport*".

We also appreciated the comments regarding the "*wide appeal*", "*broad readership of readers*", and "*broad interest*" the reviewers gave to our manuscript regarding its impact, and also the comments of "*well-written* (and) *well-cited*".

2. Description of the planned revisions

We can address all the concerns raised by the reviewers. In addition to textual changes, we will add the following to the Results section:

1. Additional quantitation of smFISH beyond Figure 2;
2. Addition of a negative (uniformly distributed) mRNA control and its quantitation;
3. Western blots for our ΔATG lines to determine what and how much protein is made.
4. Unbiased nuclear masking.

Our specific responses are shown below, in blue.

Reviewer #1

Major comments

Fig. 1: Main and supplementary figures present smFISH signals for eight localized mRNAs, while in the results section authors describe that they analyzed twenty-five transcripts. Authors should explain the choice of transcripts presented in the paper.

We will include a panel in Fig. S1E to show every mRNA that we tested, and we will edit Table 1 to describe the observed subcellular localization.

We will edit the text, adding a few sentences to clarify, along the lines of: "***Our survey revealed mRNAs with varying degrees of localization within epithelia that we divided into three classes: CeAJ/membrane localized, perinuclearly localized, and unlocalized (Fig. 1 and S1 and Table 1).***" and "***The rest of our tested mRNAs did not possess any evident subcellular localization at any of the analyzed embryonic stages/tissues and were not further investigated (Fig. S1E and Table 1).***"

© 2021. Published by The Company of Biologists under the terms of the Creative Commons Attribution License (https://creativecommons.org/licenses/by/4.0/).

Moreover, smFISH signal of different localized mRNAs in epidermal cells was visualized at different stages (bean, comma or late comma), and authors did not comment what was the reason of such conditions. This may make transcripts localization results difficult to interpret, as further analysis showed that mRNA localization varied in a stage-specific manner.

We have clarified this point now in Figure legend 1: "***Specific embryonic stages were selected for each transcript based on the highest degree of mRNA localization they exhibited.***"

Did author used smFISH probes designed against endogenous mRNAs for all tested transcripts?

We did not. We clarify this point now in Materials and methods: "*All probes were designed against the endogenous mRNA sequences except* dlg-1 *(some constructs),* pkc-3, hmp-2, spc-1, let-805, *and* vab-10a, *whose mRNA were detected with gfp probes in their corresponding transgenic lines (Table S2). An exception to this is Fig. S1A where we used probes against the endogenous* dlg- 1 *mRNA.*".

Marking dlg-1 mRNA as dlg-1-gfp suggests that smFISH probe was specific for gfp transcript. Is it true? If yes, authors should compare localization of wild-type endogenous dlg-1 mRNA with that of the transcript encoding a fusion protein, to confirm that fusion does not affect mRNA localization.

Yes, in Fig. 1C we show smFISH for GFP (*i.e.*, the tagged *dlg-1* only). In Fig. S1A, we show smFISH against endogenous *dlg-1*. Tagged and endogenous *dlg-1* mRNAs are both localized. We clarified this point in the main text: "*Five of these transcripts were enriched at specific loci at or near the cell membrane: laterally and at the CeAJ for dlg-1 (Fig. 1C for endogenous/GFP CRISPR-tagged* dlg-1::gfp *mRNA and S1A for endogenous/non-tagged* dlg-1 *mRNA), (…)*". And in the Supplemental figure legend (Fig. S1A): "*Endogenous/non-tagged* dlg-1 *mRNA shows CeAJ/membrane localization like its endogenous/GFP CRISPR-tagged counterpart.*"

Fig. 2B: Authors conclude that at later stages of pharyngeal morphogenesis mRNA enrichment at the CeAJ decreased gradually in comparison to comma stage. Data do not show statistically significant decrease in ratio of localized mRNAs - for dlg-1: bean: 0.39{plus minus}0.09, comma: 0.29{plus minus}0.08, 1.5-fold: 0.30{plus minus}0.09; for ajm-1: bean: 0.36{plus minus}0.08, comma: 0.30{plus minus}0.05, 1.5-fold: 0.28{plus minus}0.09.

t-test (one-tailed) analysis revealed a significant difference between bean and comma stages for both *dlg-1* and *ajm-1* mRNAs. Statistical analysis and data will be provided.

Fig. 4: What was the difference between the first and the second ΔATG transgenic line? Authors should analyze the size of the truncated DLG-1 protein that is expressed from the second ΔATG transgenic line that localizes to CeAJ. Knowing alternative ATGs and protein size may suggest domain composition of the truncated protein. This will allow to confront truncated protein localization with the results from.

We will perform a Western blot to determine the size and levels of proteins produced.

Fig. 5. Moreover, to prove that the localization of dlg-1 mRNA at the CeAJ is translation-dependent, additional experiment should be performed where transcripts localization will be analyzed in embryos treated with translation inhibitors such as cycloheximide (translation elongation inhibitor) and puromycin (that induces premature termination).

We believe this comment might refer to Fig. 4. If this is the case: drugs like cycloheximide and puromycin affect the translation of the whole transcriptome, whereas with our ΔATG experiment, we aimed to target the translation of one specific transcript and avoid secondary effects. Nevertheless, we understand Reviewer #1's concern and will include a second experiment. In our hands, cycloheximide and puromycin have never worked in older embryos (it's hard to get past the eggshell and into the embryo). Instead, we will use stress conditions, which induce a "ribosome drop-off" (Spriggs *et al.*, 2010). Heat stress has been shown to decrease polysome occupancy (Arnold *et al.*, 2014). We, therefore, have used heat-shock at 33°C for 30', and the results are now shown in Fig. S4. These show the loss of RNA localization upon heat shock.

© 2021. Published by The Company of Biologists under the terms of the Creative Commons Attribution License (https://creativecommons.org/licenses/by/4.0/).

Minor comments

In the introduction section authors should emphasize the main goal and scientific significance of the paper.

We added this sentence to state the significance before summarizing the results: "*To investigate the impact of mRNA localization during embryonic development, we conducted a single molecule fluorescence in situ hybridization (smFISH)-based survey* (…)" and "*Our data demonstrate that the* dlg-1 *UTRs are dispensable, whereas translation is required for localization, therefore providing an example of a translation-dependent mechanism for mRNA delivery in* C. elegans." To state the significance.

Fig 1A: It's hard to distinguish different colors on the schematics. Schematics presents intermediate filaments that are not included in the Table 1**.**

We modified Table 1 based on this and other reviewers' comments.

Fig. 1C: dlg-1 transcript is marked as dlg-1-gfp on the left panel and dlg-1 on the right panel.

Corrected.

Fig. 2B: Axis labels and titles are not visible, larger font size should be used.

We will modify the graph (following Reviewer #2's suggestion) and axes label and title sizes will be taken into account.

Fig. 5C: Enlarge the font size.

Will do.

Fig. S2: Embryonic stages should be marked on the figure for easier interpretation.

Added.

Reviewer #2

Major comments

•Figure 2 requires a negative (or uniformly distributed) mRNA control for comparison. Figure 2C should be quantified. The plot quality should be improved, and appropriate statistical tests should be employed to strengthen the claimed findings.

We will add a negative control (*jac-1* mRNA), and quantify Fig. 2C as well. Plots will be changed accordingly to the suggestion.

•Most claims of perinuclear mRNA localization are difficult to see and not well supported visually or statistically. The usage of DAPI markers, membrane markers, 3D rendering, or a quantified metric would bolster this claim. Also, sax-7 is claimed to be perinuclear and elsewhere claimed to be uniform then used as a uniform control. Please explain or resolve these discrepancies more clearly.

Regarding perinuclear mRNAs:

We are not trying to make a big statement out of these data as perinuclear (ER) localization of mRNAs coding for transmembrane/secreted proteins is well known. The aim of our study was to describe transcript localized at or in the proximity of the junction. However, we thought it was worth mentioning these examples of perinuclearly localized mRNAs (*hmr-1*, *sax-7*, and *eat-20*) for two reasons: scientific correctness – show accessory results that might be interesting for other scientists – and use as positive controls for our smFISH survey – these mRNAs were expected to

© 2021. Published by The Company of Biologists under the terms of the Creative Commons Attribution License (https://creativecommons.org/licenses/by/4.0/).

localize perinuclearly for the reasons mentioned above. We will rewrite the text to make these points clearer.

Regarding *sax-7* mRNA:

sax-7 mRNA localizes perinuclearly in sporadic instances (Fig S1C), but it is predominantly scattered throughout the cytoplasm (*i.e.*, unlocalized). It presumably localizes perinuclearly in a translation-dependent manner as *sax-7* codes for a transmembrane protein that would be targeted to the ER. We have described this ER-type of localization in the introduction and reiterated it partially in the first paragraph of the results. *sax-7* UTRs are therefore presumably not responsible for subcellular localization, which would instead depend on a signal sequence. We will better clarify this point in the main text.

- **The major concern about the paper is the data display and interpretation of Figure 5C. I'm not comfortable with the approach the authors took of blurring out the nucleus. A more faithful practice would be to use an automated mask over DAPI staining or to quantify the entirety of the cell. If the entirety of the cell were quantified, one could still focus analysis on specific regions of relevance. The interpretations distinguishing membrane versus cytoplasmic localization (or mislocalization) are hard to differentiate in these images especially since they are lacking a membrane marker. The ability to make these distinctions forms the basis of Tocchini et al's two pathways of dlg-1 mRNA localization. These interpretations also heavily rely on how the image was processed through the different Z-stacks, and it's not clear to me how that was done. For example, the diffusion of mRNA in figure 5F and 5I are indistinguishable to my eye but are claimed to be different.**

In the images, the nuclei have been blurred to allow the reader to focus on the cytoplasmic signal and not on the nuclear (transcriptional) signal as it is not meaningful for this study. In the quantitation, the nuclear signal has been unbiasedly and specifically removed from the analysis by cropping out the DNA signal from the other channels. The frontal plane views of the seam cells in Fig. 5 show maximum intensity projections (MIPs) of 3 Z-stacks (0.54 µm total) that each contain nuclei and, therefore, the transcriptional signal (schematics in Fig. 5B). We will clarify these points in the text.

Regarding cytoplasmic versus membrane-associated mRNAs, although we did not have a membrane marker, we relied on the brightness of the DLG-1::GFP signal to identify the cell borders (*i.e.*, membranes) after over-exposure. This approach allowed us to discern apicobasal and apical sides for the intensity profile analyses. We will clarify this point as well in the text and, in parallel, we will try a different approach using transverse sections on top views to clarify our data.

- **To my eye, it seems that Figure 5 could be more faithfully interpreted to state that DGL-1 protein localization depends on the L27-SH3 domains. The Huk/Guk domains are dispensable for DLG-1 protein localization; however, through other studies, we know they are important for viability. In contrast, dlg-1 mRNA localization requires all domains of the protein (L27-Guk). It is exceptionally interesting to find a mutant condition in which the mRNA and protein localizations are uncoupled. It would be very interesting to explore in the discussion or by other means what the purpose of localized translation may be. Because, in this instance, proper mRNA localization and protein function are closely associated, it may suggest that DLG-1 needs to be translated locally to function properly.**

We will rewrite the Results and Discussion to clarify our model. We agree that L27 and SH3 domains are critical, but we also detected effects of the HooK/GuK domains. We have refined our model to describe functions of the N and C termini for membrane or junctional localization.

- **The manuscript requires an improve materials & methods description of the quantification procedures and statistics employed.**

We will add these points.

Minor & Major comments together - text

© 2021. Published by The Company of Biologists under the terms of the Creative Commons Attribution License (https://creativecommons.org/licenses/by/4.0/).

- **Summary statement: Is "adherent junction" supposed to be "adherens junction?"**

Corrected.

- **Abstract: Sentence 1, I think they should add a caveat word to this sentence. Something like "...phenomenon that can facilitate sub-cellular protein targeting." In most instances this isn't very well characterized or known.**

Corrected.

- **In the first paragraph, it might be good to mention that Moor et al also showed that mRNA localize to different regions to alter their level of translation (to concentrate them in high ribosome dense regions of the cell).**

Added as follows: ***"For example, a global analysis of localized mRNAs in murine intestinal epithelia found that 30% of highly expressed transcripts were polarized and that their localization coincided with highly abundant regions in ribosomes (Moor, 2017)."***

- **There are some new studies of translation-dependent mRNA localization - that might be good to highlight - Li et al., Cell Reports (PMID: 33951426) 2021; Sepulveda et al., 2018 (PCM), Hirashima et al., 2018; Safieddine, et al 2021. Also, Hughes and Simmonds, 2019 reviews membrane associated mRNA localization in Drosophila. And a new review by Das et al (Nat Rev MCB) 2021 is also nice.**

We will add them to the text.

- **Parker et al. did not show that the 3'UTR was dispensable for mRNA localization. They showed the 3'UTR was sufficient for mRNA localization.**

Quoting from the paper Parker *et al.*: *"3'UTRs of erm-1 and imb-2 were not sufficient to drive mRNA subcellular localization. Endogenous erm-1 and imb-2 mRNAs localize to the cell or nuclear peripheries, respectively, but mNeonGreen mRNA appended with erm-1 or imb- 2 3'UTRs failed to recapitulate those patterns (Fig. 4A-D)."* We will make this point clearer in the rewritten text.

- **In the second paragraph, the sentence about bean stages is missing one closing parenthesis.**

Corrected.

- **Last paragraph: FISH is fluorescence, not fluorescent.**

Corrected.

- **Both "subcellular" and "sub-cellular" are used.**

Corrected.

Minor comments – Figures

- **Figure 1**

○ **Figure 1A is confusing. It's not totally clear what the rectangles and circles signify. There are many acronyms within the figure. Which of the cell types depicted in the figure are shown here? For example, for the dorsal cells, which is the apical v. basal side?**

We tried to simplify the cartoon for a general *C. elegans* epithelial cell. We followed schematics already shown in previous publications to maintain consistency. Acronyms and color-codes are listed in the corresponding figure legend and have been better clarified.

© 2021. Published by The Company of Biologists under the terms of the Creative Commons Attribution License (https://creativecommons.org/licenses/by/4.0/).

○ **Some of the colors are difficult to distinguish, particularly when printed out or for red/green colorblind readers. Is erm-1 meant to be a cytoskeletal associated or a basolateral polarity factor?**

We understand the issue, but unfortunately, with 8 classes of factors, shades of gray might not solve the problem. We tried to circumvent the red-green issue changing red to dark grey. Furthermore, we added details about shapes to the figure legends. We will work to make the colors work better.

ERM-1 is a cytoskeletal-associated factor.

○ **The nomenclature for dlg-1 is inconsistent within "C".**

Corrected.

○ **Please specify what the "cr" is in "cr.dlg-1:-gfp" in the legend.**

Added.

- **Figure 2**

○ **Can Figure 2C be quantified in a similar manner to 2A/2B?**

Currently our script cannot do that, but we will try to optimize it to be able to quantify this type of images.

○ **2B - please jitter the dots to better visualize them when they land on top of one another**

Yes, we will.

○ **Please include a negative control example, a transcript that is not peripherally localized for comparison.**

○ Yes, we will.

○ **There is no place in the text of the document where Fig 2C is referenced**

Corrected (it was wrongly referred to as “2B”).

○ **I can't see any discernable ajm-1 localization in Fig 2A.**

We added some arrowheads to point at specific examples and increased the intensities of the corresponding smFISH signal for better visualization.

○ **I can't see any dlg-1 pharyngeal localization in Fig2C.**

We added some arrowheads to point at specific examples and increased the intensities of the corresponding smFISH signal for better visualization.

○ **More details on how the quantification was performed would be welcome. Particularly, in 2B, what is the distance from the membrane in which transcripts were called as membrane-associated? What statistics were used to test differences between groups?**

We will add a full description of the script used as well as the statistic details.

- **Figure 3**

○ **Totally optional but might be nice: can you make a better attempt to approximate the scale of the cartoon depiction?**

© 2021. Published by The Company of Biologists under the terms of the Creative Commons Attribution License (https://creativecommons.org/licenses/by/4.0/).

The UTRs, especially the 5' one, are much smaller than the *dlg-1* gene sequence. A proper scaling of the cartoon to the actual sequences, would draw the attention away from the main subjects of this figure, the UTRs. Nevertheless, we made sure it is clear in the corresponding figure legend that the cartoon is not in scale: "*The schematics are not in scale with the actual size of the corresponding sequences. UTR lengths:* dlg-1 *5'UTR: 61 nucleotides;* sax-7 *5'UTR: 63 nucleotides;* dlg-1 *3'UTR: 815 nucleotides;* unc-54 *3'UTR: 280 nucleotides.*"

○ **The GFP as an asterisk illustration may be confusing for some readers. Could you add another rectangular box to depict the gfp coding sequence?**

Corrected.

○ **This microscopy is beautiful!**

Thanks Reviewer #2!

○ **Were introns removed? Is the endogenous copy still present?**

All the transgenes were analyzed in a wild-type background, therefore, yes, the endogenous copy was still present. All the transgenes possessed introns. We will change the corresponding text as follows: "*To test whether the localization of one of the identified localized mRNAs,* dlg-1, *relied on zip codes, we generated extrachromosomal transgenic lines carrying a* dlg-1 *gene whose sequence was fused to an in-frame GFP and to exogenous UTRs.*". In the figure "*dlg-1* ORF" has been replaced with "*dlg-1* gene".

○ **The wording in the legend "CRISPR or transgenic" may be confusing as Cas9 genome editing is still a form of transgenesis.**

We added "extrachromosomal" to clarify the nature of the mRNA.

○ **The authors state that the 5'-3'UTR construct produces perinuclear dlg-1 transcripts but in the absence of DAPI imaging, it's not clear that this is the case.**

We could not find such a statement, but we tried to clarify the localization of these mRNAs in the text: "*The mRNA localization patterns of the two UTR reporters were compared to the localization of* dlg-1 *transcripts from the CRISPR line ("wild-type", Fig. 3A; Heppert* et al.*, 2018), described in Fig. 2. Both reporter strains showed enrichment at the* CeAJ *and localization dynamics of their transcripts that were comparable to the wild-type* cr.dlg-1 *(Fig. 3B). These results indicate that the UTR sequences of* dlg-1 *mRNA are not required for its localization.*"

○ **Which probe set was used? The gfp probe?**

Yes, please see the main text: "*Given that the transgenic constructs were expressed in a wild-type background, smFISH experiments were conducted with probes against GFP RNA sequences to focus on the transgenic* dlg-1::GFP *mRNAs (*cr.dlg-1 *and* tg.dlg-1*).*"

○ **Here, sax-7 is used as a uniform control, but sax-7 is claimed in Fig S1B-D as being perinuclear. This is a bit confusing.**

sax-7 mRNA localizes perinuclearly in sporadic instances (Fig S1C), but it is predominantly scattered throughout the cytoplasm (*i.e.*, unlocalized). It presumably localizes perinuclearly in a translation-dependent manner as *sax-7* codes for a transmembrane protein that would be targeted to the ER. We have described this ER-type of localization in the introduction and reiterated it partially in the first paragraph of the results. *sax-7* UTRs are therefore presumably not responsible for any subcellular localization, which would instead rely on a signal sequence. We will better clarify this point in the main text.

- **Figure 4**

○ **Excellent results! Really nice!**

© 2021. Published by The Company of Biologists under the terms of the Creative Commons Attribution License (https://creativecommons.org/licenses/by/4.0/).

Thanks Reviewer #2!

○ **Fig 4A. The GFP depicted as a circle is strange.**

We changed it into a rectangle.

○ **Fig 4A. Can you include the gene/protein name for easy skimming?**

Added.

○ **Fig 4B. the color here is too faint and it is unclear what is being depicted. Overall, this part of the figure could be improved.**

We are optimizing the coloring and simplifying the schematics.

○ **Were the introns removed?**

No, the introns were maintained in this and in all our transgenic lines. We described our transgenic lines in the materials and methods section (now with more detail). What we depict in the scheme (Fig. 4A) is the mature RNA (now specified in the figure), therefore no introns depicted. We will also specify this in the main text.

- **Figure 5**

○ **Fig 5A. can you add the gene/protein name**

Added.

○ **Fig 5B. Can you make the example apicobasal (non-apical) mRNA more distinctive? If it had its own peak in the lower trace, the reader would more clearly understand that this mRNA will be excluded from apical measurements whereas it will be included in apicobasal measurements.**

We actually wanted to show this specific example: a cytoplasmic mRNA and a junctional mRNA may seem close from the apicobasal analysis (partially overlapping peaks that Reviewer #2 mentioned). With the apical analysis, instead, we can show that these mRNAs are actually not close, and they belong to two different compartments (cytoplasm and junction). We would therefore like to keep the current scheme, while better clarifying this point in the corresponding figure legend.

○ **D' - I' The grey font is too light.**

Noted. We will change it.

○ **D' - I' The inconsistent y-axis scaling makes it difficult to compare across these samples. Can you set them to the same maximum number?**

The values are indeed quite different. We tried to use the same scale, but this would make some of the data unappreciable. The idea was to evaluate, within each graph, how mRNA and protein are localized relative to the junctional marker. We will make this clearer in the text.

○ **D' - I' The x-axis labels are formatted incorrectly**

Corrected.

○ **The practice of masking out the nucleus appears to remove potentially important mRNAs that are not nuclear localized. This could really impact the findings and interpretation. Instead, consider an automated DAPI mask.**

The masking on the images is not the same used for the analysis: in the images, a shaded circle has

© 2021. Published by The Company of Biologists under the terms of the Creative Commons Attribution License (https://creativecommons.org/licenses/by/4.0/).

been drawn on the DNA channel and moved onto its corresponding location in the other channels or merges. For the analysis, the DNA signal has been specifically removed in the channel with the smFISH signal. Given that the analysis has been performed on maximum intensity projections of 3 Z-stacks, we believe we did not remove any non-nuclear mRNA. We will clarify this point in Materials and methods.

○ **I can't see what the authors are calling membrane diffuse versus cytoplasmic. This is making it hard for me to see their "two step" pathway to localization.**

We will add in Fig. 5B-C an example of a membrane localized mRNA. Furthermore, we will add transverse sections of membrane and cytoplasm to make the date clearer to the reader.

○ **Can more details of the quantification be included? How were Z-sections selected, chosen for inclusion? Which Z-sections and how many were selected?**

We will add the details to Materials and methods.

○ **Also, why do these measurements focus on what I think are the seam cells when Lockwood et al., 2008 show the entire epithelium that is much easier to see?**

We are focusing on the seam cells at the bean stage as these are the cells and the embryonic stage where we see the highest localization of *dlg-1* mRNA in the wild-type.

○ **Please name these constructs to correlate the text more explicitly to the figures.**

Added.

○ **How many embryos were analyzed for each trace? How many embryos showed consistent patterns?**

We will add the details of the analysis to Materials and methods.

○ **Why were these cells used for study here? Lockwood et al., 2008 use a larger field of epithelial cells for visualization.**

As stated before: we are focusing on the seam cells at the bean stage as these are the cells and the embryonic stage where we see the highest localization of *dlg-1* mRNA in the wild-type.

- **Figure 6**

There are major discrepancies between what this figure is depicting graphically and what is described in the text. Again, I'm not comfortable making the "two step" claims this figure purports given the data shared in Figure 5.

We are planning to re-write the last part of the results to better clarify our two-step model. A two-step model had been previously suggested in McMahon *et al.*, 2001, where they could show that DLG-1 and AJM-1 (referred to in that publication as JAM-1) are initially localized laterally and only later in development are then enriched apically. Our data agree with McMahon very well, so we used the earlier study as a start. We will cite and explain this paper in greater depth during the rewriting.

Minor comments - Tables & Supplemental Figures

Table 1

- **I think this table could be improved to more clearly illustrate which mRNAs were tested and what their mRNA localization patterns were (for example, gene name identifiers included, etc). Could the information that is depicted by gray shading instead be added as its own column? For example, have a column for "Observed mRNA localization"**

© 2021. Published by The Company of Biologists under the terms of the Creative Commons Attribution License (https://creativecommons.org/licenses/by/4.0/).

We modified Table 1 based on these and the other reviewers' comments.

- **Can you add distinct column names for the two columns that are labeled as "protein localization - group"**

We modified Table 1 based on these and the other reviewers' comments.

- **Can you also add which of these components are part of ASI v. ASII (as described in the introduction?)**

A new table has been added with the factors belonging to the two adhesion systems (same color code as in Table 1).

Supplemental Figure 1

- **It is hard to see that some of these spots are perinuclear. More information (membrane marker, 3D rendering, improved metrics) is required to support this claim.**

We are not trying to make a big statement out of these data as perinuclear localization for mRNAs coding for transmembrane/secreted proteins is well known. The aim of our study was to describe transcript localized at or in the proximity of the junction. We thought it was worth mentioning these examples of perinuclearly localized mRNAs (*hmr-1*, *sax-7*, and *eat-20*) for two reasons: scientific correctness - show accessory results that might be interesting for other scientists - and use as positive controls for our smFISH survey - these mRNAs were expected to have a somewhat perinuclear localization for the reasons mentioned above.

- **What do these images look like over the entire embryo, not just in the zoomed in section?**

We added a column with the zoom-out embryos.

- **sax-7 localization in S4 looks similar but a different localization claim is made.**

sax-7 mRNA can localize perinuclearly in sporadic instances (Fig S1C), but is predominantly scattered throughout the cytoplasm (*i.e.*, unlocalized). It presumably localizes perinuclearly in a translation-dependent manner as *sax-7* codes for a transmembrane protein that would be targeted to the ER. We have described this ER-type of localization in the introduction and reiterated it partially in the first paragraph of the results. *sax-7* UTRs are therefore presumably not responsible for any subcellular localization, which would instead rely on a signal sequence. We will better clarify this point in the main text.

Supplemental Figure 2

- **Before adherens junctions even exist dlg-1 go to the membrane - this is really neat!**

Thanks Reviewer #2!

Supplemental Figure 3

- **Technical question: If either 5 or 3 stack images are used, how does this work? Do they have different z-spacings? Or do they do 5-stack images represent a wider Z-space?**

This is the sentence under question: "*Maximum intensity projections of 5 (1.08 μm) (A) and 3 (0.54 μm) (B) Z-stacks*". The space between each Z-stack image is constant in all our imaging and its value is 270 nm. When we consider 5 planes, the distance from the 1st to the 5th is 4 x 270 nm = 1.08 μm, whereas for 3 planes will be 2 x 270 nm = 0.54 μm.

Supplemental Figure 4

- **Line #2 retains translation and keeps mRNA localization.**

© 2021. Published by The Company of Biologists under the terms of the Creative Commons Attribution License (https://creativecommons.org/licenses/by/4.0/).

- **Totally optional, but consider showing both lines in the main figure to illustrate the two possibilities.**

Noted.

- **Materials and methods - how did they created the ATG mutations? Is it an array? - why does one translate, and one doesn't?**

We will clarify this point in Materials and methods: "dlg-1 *deletion constructs ΔATG (SM2664 and SM2663) and ΔL27-PDZs (SM2641) were generated by overlap extension PCR using pML902 as a template.*".

We will perform a Western blot to clarify Reviewer #2's last point. Currently we do not know what peptide is translated, but the comparison with our full-length control will probably shed some light on the issue.

Reviewer #3

Major comments

1. **The smFISH results are striking and implications exciting. The conclusions made from the smFISH results reported in all Figures will be strengthened considerably by quantifying the mRNA localized to the defined specific subcellular regions. At the very least, localization to the cytoplasm versus the plasma membrane should be determined as performed in Figure 2B, but quantifying finer localization will enhance the conclusions made about regional localization (e.g. CeAJ versus plasma membrane mRNA localization in Figure 5). Inclusion of a non-localizing control in Figures 1-4 will enable statistical comparisons between mRNA localizing and non-localizing groups.**

   We will add more quantitation, statistics, and negative controls.

2. **The script used for smFISH quantitation should be included in the methods or published in an accessible forum (Github, etc). Criteria for mRNA "dot" calling should be defined in the methods. All raw smFISH counts should also be reported.**

   We will add the full description of the script in Materials and methods, and we will provide the raw data in an additional supplementary table.

3. **Figure 2: What is the localizing ratio of a non-localizing control mRNA (e.g. jac-1)? Including an unlocalized control with quantitation would strengthen the localization arguments presented.**

   Yes, we will add quantitation for an unlocalized mRNA.

4. **Figure 5: Quantifying colocalization of mRNA and protein (+/- AJM-1) will strengthen the arguments made about mRNA/protein localization.**

   Yes, we will quantify Fig. S5 to have a full picture of the cells (the images in Fig. 5 represent only a portion of the cell).

5. **Discussion of the CeAJ mRNA localization mechanism is warranted. Do the authors speculate that the newly translated protein drives localization during translation, similar in concept to SRP-mediated localization to the ER, or ribosome association is a trigger to permit a secondary factor to drive mRNA localization, or another model?**

   Unfortunately, this is hard to say at the moment as we do not have any data regarding where translation actually occurs. We will add a conjecture to the Discussion.

© 2021. Published by The Company of Biologists under the terms of the Creative Commons Attribution License (https://creativecommons.org/licenses/by/4.0/).

Minor comments

1. Please complete the following sentence: "We identified transcripts enriched at the CeAJ in a stage- and cell type-specific."

Corrected.

2. It would be helpful to provide reference(s) for the protein localization summary in Table 1.

Added.

3. Figure 2B: Did dlg-1 and ajm-1 localize at similar ratios? Appropriate statistics comparing the different ratios may be informative.

We will modify the graph (following Reviewer #2's suggestion) and add the requested details.

4. Figure 2: In the paragraph that begins, "Morphogenesis of the digestive track," the text should refer to Figure 2C? If not, the text requires further clarification.

Corrected.

5. Figure 2: Reporting the smFISH localizing ratios of 8E and 16E will be informative.

We will add the information.

6. Please include citations when summarizing the nonsense-mediated decay NMD mechanism and AJM-1 identifying the CeAJ.

Added.

7. The sentence, "Embryos from our second ΔATG transgenic line displayed a little GFP protein and some dlg-1::gfp mRNA," should refer to Figure S4.

Added.

8. An immunoblot of this reporter versus wild type may be informative regarding the approximate position of putative alternative start codon.

We will perform a Western blot to verify the size of the protein product produced.

9. Figure 5: N's and repetitions performed should be included for localization experiments.

Yes, we will add them here and in all the other quantifications we will add to the manuscript.

10. Please clarify that the "the mechanism of UTR-independent targeting is unknown in any species" refers to dlg-1 mRNA localization.

Added.

11. "Our findings suggest..." discussion paragraph should reference Figure 6.

Added.

3. Description of the revisions that have already been incorporated in the transferred manuscript

4. Description of analyses that authors prefer not to carry out

© 2021. Published by The Company of Biologists under the terms of the Creative Commons Attribution License (https://creativecommons.org/licenses/by/4.0/).

Original submission

First decision letter

MS ID#: DEVELOP/2021/200027

MS TITLE: Translation-dependent mRNA localization to Caenorhabditis elegans adherens junctions

AUTHORS: Cristina Tocchini, Michèle Rohner, Stephen E Von Stetina, and Susan E Mango

Thank you submitting your manuscript to Development with the assessment from Review Commons and a potential revision plan. I have reviewed the reviewer comments and your response with the revision plan. I invite you to submit a revision of your manuscript following the revision plan that you have outlined.

Please attend to all of the reviewers' comments and ensure that you clearly highlight all changes made in the revised manuscript. Please avoid using 'Tracked changes' in Word files as these are lost in PDF conversion. I should be grateful if you would also provide a point-by-point response detailing how you have dealt with the points raised by the reviewers in the 'Response to Reviewers' box. If you do not agree with any of their criticisms or suggestions please explain clearly why this is so.

First revision

Author response to reviewers' comments

We were delighted the reviewers found our results "*compelling*", "*striking*", "*well presented*", "*implications exciting*", "*excellent results! really nice!*", "*this microscopy is beautiful*!" and "*translational-dependence* (of mRNA localization) *in a transcript-specific way without perturbing translation globally*", which is a "*complete surprise, and opens exciting doors to investigate how translation leads to mRNA organization and its connection to tissue development*" and "*may represent a new pathway of mRNA transport*".

We have added the following to the Results section, as requested:

1. Additional quantitation of smFISH beyond Figure 2;
2. Addition of a negative (uniformly distributed) mRNA control and its quantitation;
3. Unbiased nuclear masking;
4. Additional experiment to confirm the involvement of translation in *dlg-1* mRNA localization (inhibition of total translation; Fig. S6);
5. Addition of quantitation of DLG-1::GFP intensities in ΔATG line2 compared to controls (Fig. S5).

Our specific responses are shown below, in blue.

Reviewer #1

Major comments

Fig. 1: Main and supplementary figures present smFISH signals for eight localized mRNAs, while in the results section authors describe that they analyzed twenty-five transcripts. Authors should explain the choice of transcripts presented in the paper.

We included an additional supplemental figure (Fig. S2) to show the additional mRNAs that we tested (looking at examples for both epidermal and pharynx/intestine).

We edited Table 1 to describe the observed subcellular localization plus additional columns, as

© 2021. Published by The Company of Biologists under the terms of the Creative Commons Attribution License (https://creativecommons.org/licenses/by/4.0/).

requested by the other reviewers.

We edited the text, adding a few sentences to clarify our choice:

1. *"Our survey revealed mRNAs with varying degrees of localization within epithelia that we divided into three classes: CeAJ/membrane localized, perinuclearly localized, and unlocalized (Fig. 1B-G, S1, and S2 and Table 1)"*
2. *"The rest of our tested mRNAs did not possess any evident subcellular localization at any of the analyzed embryonic stages/tissues and were not further investigated (Fig. S2 and Table 1)."*

Moreover, smFISH signal of different localized mRNAs in epidermal cells was visualized at different stages (bean, comma or late comma), and authors did not comment what was the reason of such conditions. This may make transcripts localization results difficult to interpret, as further analysis showed that mRNA localization varied in a stage-specific manner.

We have clarified this point now in Figure legend 1: "*Specific embryonic stages were selected for each transcript based on the highest degree of mRNA localization they exhibited.*"

Did author used smFISH probes designed against endogenous mRNAs for all tested transcripts?

We clarify this point now in Table 1 and Materials and methods: "*All probes were designed against the endogenous mRNA sequences except* dlg-1 *(some constructs),* pkc-3, hmp-2, spc-1, let-805, *and* vab-10a, *whose mRNA were detected with gfp probes in their corresponding transgenic lines (Table S3). Exception to this are Fig. S1A and S3A where we used probes against the endogenous* dlg-1 *mRNA.*".

Marking dlg-1 mRNA as dlg-1-gfp suggests that smFISH probe was specific for gfp transcript. Is it true? If yes, authors should compare localization of wild-type endogenous dlg-1 mRNA with that of the transcript encoding a fusion protein, to confirm that fusion does not affect mRNA localization.

Yes, in Fig. 1C we show smFISH for GFP (*i.e.*, the tagged *dlg-1* only). In Fig. S1A, we show smFISH against endogenous *dlg-1*. Tagged and endogenous *dlg-1* mRNAs are both localized. We clarify this point in the main text: "*Five of these transcripts were enriched at specific loci at or near the cell membrane: laterally and at the CeAJ for dlg-1 (Fig. 1C for endogenous/GFP CRISPR-tagged* dlg-1::gfp *mRNA and S1A for endogenous/non-tagged* dlg-1 *mRNA), (…)*". And in the Supplemental figure legend (Fig. S1A): "*Endogenous/non-tagged* dlg-1 *mRNA shows CeAJ/membrane localization like its endogenous/GFP CRISPR-tagged counterpart.*"

Fig. 2B: Authors conclude that at later stages of pharyngeal morphogenesis mRNA enrichment at the CeAJ decreased gradually in comparison to comma stage. Data do not show statistically significant decrease in ratio of localized mRNAs - for dlg-1: bean: 0.39{plus minus}0.09, comma: 0.29{plus minus}0.08, 1.5-fold: 0.30{plus minus}0.09; for ajm-1: bean: 0.36{plus minus}0.08, comma: 0.30{plus minus}0.05, 1.5-fold: 0.28{plus minus}0.09.

We re-analyzed our images using the well-established software FISH-quant to analyze the images, and we ran t-tests on the new data for statistical significance, now shown in Fig. 2B,D (plus corresponding figure legend) and Table S1.

Fig. 4: What was the difference between the first and the second ΔATG transgenic line? Authors should analyze the size of the truncated DLG-1 protein that is expressed from the second ΔATG transgenic line that localizes to CeAJ. Knowing alternative ATGs and protein size may suggest domain composition of the truncated protein. This will allow to confront truncated protein localization with the results from.

The construct and its co-injection markers used to generate the two lines were the same, the lines show slight differences, which can happen with c. elegans transgenics.
We tried to perform Western blots to identify the length of the poorly translated DLG-1::GFP protein in the second line described in the paper ("line2"), but unfortunately had no success. This may be due to the low level of protein produced. Currently, we cannot explain why one of the two

© 2021. Published by The Company of Biologists under the terms of the Creative Commons Attribution License (https://creativecommons.org/licenses/by/4.0/).

lines is able to express DLG-1::GFP protein to a very small extent while the other is not (now quantified in Fig. S5B). Perhaps line 2 has more RNA expression, making the probability for some protein production higher. More mRNA could be due to a higher copy number of the transgene or a more active configuration of the array.

Fig. 5. Moreover, to prove that the localization of dlg-1 mRNA at the CeAJ is translation-dependent, additional experiment should be performed where transcripts localization will be analyzed in embryos treated with translation inhibitors such as cycloheximide (translation elongation inhibitor) and puromycin (that induces premature termination).

Drugs like cycloheximide and puromycin affect the translation of the whole transcriptome, whereas with our ΔATG experiment, we aimed to target the translation of one specific transcript and avoid secondary effects. Nevertheless, we understand Reviewer #1's concern and included a second experiment. In our hands, cycloheximide and puromycin have never worked in older embryos (it is hard to get past the eggshell and into the embryo). Instead, we used stress conditions, which induce a "ribosome drop-off" (Spriggs *et al.*, 2010). Heat stress has been shown to decrease polysome occupancy (Arnold *et al.*, 2014). We, therefore, used two heat-shock conditions: one at 34°C and another one at 37°C for one hour on plates. The results (images and quantitation) are now shown in Fig. S6. These results show the loss of RNA localization upon heat-shock, as expected.

Minor comments

In the introduction section authors should emphasize the main goal and scientific significance of the paper.

We added this sentence to state the significance before summarizing the results: "*To investigate the existence of mRNA localization during embryonic development, we conducted a single molecule fluorescence in situ hybridization (smFISH)-based survey* (…)" and "*Our data demonstrate that the* dlg- 1 *UTRs are dispensable, whereas translation is required for localization, therefore providing an example of a translation-dependent mechanism for mRNA delivery in* C. elegans." to state the significance.

Fig 1A: It's hard to distinguish different colors on the schematics. Schematics presents intermediate filaments that are not included in the Table 1.

We modified Table 1 based on this and other reviewers' comments.

Fig. 1C: dlg-1 transcript is marked as dlg-1-gfp on the left panel and dlg-1 on the right panel.

Corrected.

Fig. 2B: Axis labels and titles are not visible, larger font size should be used.

Corrected.

Fig. 5C: Enlarge the font size.

Corrected (now Fig. 6)

Fig. S2: Embryonic stages should be marked on the figure for easier interpretation.

Added (now Fig. S3).

Reviewer #2

Major comments

- Figure 2 requires a negative (or uniformly distributed) mRNA control for comparison. Figure 2C should be quantified. The plot quality should be improved, and appropriate statistical tests should

© 2021. Published by The Company of Biologists under the terms of the Creative Commons Attribution License (https://creativecommons.org/licenses/by/4.0/).

be employed to strengthen the claimed findings.

Done.

• Most claims of perinuclear mRNA localization are difficult to see and not well supported visually or statistically. The usage of DAPI markers, membrane markers, 3D rendering, or a quantified metric would bolster this claim. Also, sax-7 is claimed to be perinuclear and elsewhere claimed to be uniform then used as a uniform control. Please explain or resolve these discrepancies more clearly.

Regarding perinuclear mRNAs:

We are not trying to make a big statement out of these data as perinuclear (ER) localization of mRNAs coding for transmembrane/secreted proteins is well known. The aim of our study was to describe transcripts localized at or in the proximity of the junction. However, we thought it was worth mentioning these examples of perinuclearly localized mRNAs (*hmr-1*, *sax-7*, and *eat-20* – now also *let- 805::gfp* that had escaped our previous analysis) for two reasons: scientific correctness – show accessory results that might be interesting for other scientists – and use as positive controls for our smFISH survey – these mRNAs were expected to localize perinuclearly but such localization depends on the signal peptide located in the coding sequence and not on UTRs.

Regarding *sax-7* mRNA, specifically:

sax-7 mRNA localizes perinuclearly in sporadic instances (Fig S1B), but it is predominantly scattered throughout the cytoplasm (*i.e.*, unlocalized). Perhaps these unlocalized RNAs are not being translated. *sax-7* RNA presumably localizes perinuclearly in a translation-dependent manner as *sax-7* codes for a transmembrane protein that would be targeted to the ER. We have described this ER-type of localization in the introduction and reiterated it partially in the first paragraph of the results. *sax-7* UTRs are therefore presumably not responsible for subcellular localization, which would instead depend on a signal sequence.

We added sentences to clarify these points:

1. *"We used UTRs from mRNAs that do not localize near cell membranes, namely sax-7 and unc- 54 (Fig. S4A,B)." (main text)*
2. *"Such a localization depends on the signal peptide located in the CDS of sax-7 and not on its UTRs." (Figure legend S4)*

• The major concern about the paper is the data display and interpretation of Figure 5C. I'm not comfortable with the approach the authors took of blurring out the nucleus. A more faithful practice would be to use an automated mask over DAPI staining or to quantify the entirety of the cell. If the entirety of the cell were quantified, one could still focus analysis on specific regions of relevance. The interpretations distinguishing membrane versus cytoplasmic localization (or mislocalization) are hard to differentiate in these images especially since they are lacking a membrane marker. The ability to make these distinctions forms the basis of Tocchini et al's two pathways of dlg-1 mRNA localization. These interpretations also heavily rely on how the image was processed through the different Z-stacks, and it's not clear to me how that was done. For example, the diffusion of mRNA in figure 5F and 5I are indistinguishable to my eye but are claimed to be different.

In the images, the nuclei have been blurred to allow the reader to focus on the cytoplasmic signal and not on the nuclear (transcriptional/nuclear tansport) signal as it is very bright and yet not meaningful for this study. In the quantitation, the nuclear signal has been unbiasedly and specifically removed from the analysis by cropping out the DNA signal from the other channels (see Fig. S9 and its corresponding legend, and Materials and methods). In short, signal that overlapped with DAPI in Z- stack sections was removed (*i.e.*, not the max intensity projection because that would erroneously remove cytoplasmic signal as well). The frontal plane views of the seam cells in Fig. 6 show maximum intensity projections (MIPs) of 3 Z-stacks (0.54 µm total) that each contain nuclei and, therefore, the transcriptional signal (schematics in Fig. 6B). We clarified these points in the text.

© 2021. Published by The Company of Biologists under the terms of the Creative Commons Attribution License (https://creativecommons.org/licenses/by/4.0/).

Regarding cytoplasmic versus membrane-associated mRNAs, although we did not have a membrane marker, over-exposure of the DLG-1::GFP signal was used to identify the cell borders (*i.e.*, membranes). This approach allowed us to discern apicobasal and apical sides for the intensity profile analyses. Note that over-exposure was not used to analyze localization, only to define the cell.

- To my eye, it seems that Figure 5 could be more faithfully interpreted to state that DGL-1 protein localization depends on the L27-SH3 domains. The Huk/Guk domains are dispensable for DLG-1 protein localization; however, through other studies, we know they are important for viability. In contrast, dlg- 1 mRNA localization requires all domains of the protein (L27-Guk). It is exceptionally interesting to find a mutant condition in which the mRNA and protein localizations are uncoupled. It would be very interesting to explore in the discussion or by other means what the purpose of localized translation may be. Because, in this instance, proper mRNA localization and protein function are closely associated, it may suggest that DLG-1 needs to be translated locally to function properly.

We have re-written the last two paragraphs of the result part and added another main figure (Fig.5) to better clarify our interpretation of the data. We agree that L27 and SH3 make contributions, and we clarify this now.

- The manuscript requires an improve materials & methods description of the quantification procedures and statistics employed.

Done.

Minor & Major comments together - text

- Summary statement: Is "adherent junction" supposed to be "adherens junction?"

Corrected.

- Abstract: Sentence 1, I think they should add a caveat word to this sentence. Something like "...phenomenon that can facilitate sub-cellular protein targeting." In most instances this isn't very well characterized or known.

Corrected.

- In the first paragraph, it might be good to mention that Moor et al also showed that mRNA localize to different regions to alter their level of translation (to concentrate them in high ribosome dense regions of the cell).

Added as follows: "*For example, a global analysis of localized mRNAs in murine intestinal epithelia found that 30% of highly expressed transcripts were polarized and that their localization coincided with highly abundant regions in ribosomes (Moor, 2017).*"

- There are some new studies of translation-dependent mRNA localization - that might be good to highlight – Li et al., Cell Reports (PMID: 33951426) 2021; Sepulveda et al., 2018 (PCM), Hirashima et al., 2018; Safieddine, et al 2021. Also, Hughes and Simmonds, 2019 reviews membrane associated mRNA localization in Drosophila. And a new review by Das et al (Nat Rev MCB) 2021 is also nice.

Safieddine *et al.*, 2021 and Hirashima *et al.*, 2018 were already present in the text. We added most of the other suggested references.

- Parker et al. did not show that the 3'UTR was dispensable for mRNA localization. They showed the 3'UTR was sufficient for mRNA localization.

Quoting from the paper Parker *et al.*: "*3'UTRs of* erm-1 *and* imb-2 *were not sufficient to drive mRNA subcellular localization. Endogenous* erm-1 *and* imb-2 *mRNAs localize to the cell or nuclear peripheries, respectively, but* mNeonGreen *mRNA appended with* erm-1 *or* imb-2 *3'UTRs failed to*

© 2021. Published by The Company of Biologists under the terms of the Creative Commons Attribution License (https://creativecommons.org/licenses/by/4.0/).

recapitulate those patterns (Fig. 4A-D).” We clarified this better in the text: *“A recent study on the early C. elegans embryo demonstrated the dispensability of 3’UTR to localize at least two mRNAs (erm-1 and imb-2 (Parker et al., 2020)).”*

- In the second paragraph, the sentence about bean stages is missing one closing parenthesis.

Corrected.

- Last paragraph: FISH is fluorescence, not fluorescent.

Corrected.

- Both "subcellular" and "sub-cellular" are used.

Corrected.

Minor comments – Figures

- Figure 1

○ Figure 1A is confusing. It's not totally clear what the rectangles and circles signify. There are many acronyms within the figure. Which of the cell types depicted in the figure are shown here? For example, for the dorsal cells, which is the apical v. basal side?

We tried to simplify the cartoon for a general *C. elegans* epithelial cell. We followed schematics already shown in previous publications to maintain consistency. Acronyms and color-codes are listed in the corresponding figure legend and have been better clarified.

○ Some of the colors are difficult to distinguish, particularly when printed out or for red/green colorblind readers. Is erm-1 meant to be a cytoskeletal associated or a basolateral polarity factor?

We understand the issue, but unfortunately, with 8 classes of factors, shades of gray might not solve the problem. We tried to circumvent the red-green issue changing red to black. Furthermore, we added details about shapes to the figure legends and changed additional colors/shapes. ERM-1 is a cytoskeletal-associated factor.

○ The nomenclature for dlg-1 is inconsistent within "C".

Corrected.

○ Please specify what the "cr" is in "cr.dlg-1:-gfp" in the legend.

Added.

- Figure 2

○ Can Figure 2C be quantified in a similar manner to 2A/2B?

We employed now FISH-quant for all our analyses and managed to add the 16E stage to Fig. 2B and analyze comma and 1.5-fold in Fig. 2D (for panel C - pharynx).

○ 2B - please jitter the dots to better visualize them when they land on top of one another
Corrected.

○ Please include a negative control example, a transcript that is not peripherally localized for comparison.

Added.

© 2021. Published by The Company of Biologists under the terms of the Creative Commons Attribution License (https://creativecommons.org/licenses/by/4.0/).

○ There is no place in the text of the document where Fig 2C is referenced

Corrected (it was wrongly referred to as “2B”).

○ I can't see any discernable ajm-1 localization in Fig 2A.

We added some arrowheads to point at specific examples and increased the intensities of the corresponding smFISH signal for better visualization.

○ I can't see any dlg-1 pharyngeal localization in Fig2C.

We added some arrowheads to point at specific examples and increased the intensities of the corresponding smFISH signal for better visualization.

○ More details on how the quantification was performed would be welcome. Particularly, in 2B, what is the distance from the membrane in which transcripts were called as membrane-associated? What statistics were used to test differences between groups?

As mentioned before, we re-analyzed all our images with FISH-quant as being a well-established tool to quantify smFISH signals. We have now fully described how quantification was performed in Materials and methods.

- Figure 3

○ Totally optional but might be nice: can you make a better attempt to approximate the scale of the cartoon depiction?

The UTRs, especially the 5’ one, are much smaller than the *dlg-1* gene sequence. A proper scaling of the cartoon to the actual sequences, would draw the attention away from the main subjects of this figure, the UTRs. Nevertheless, we made sure it is clear in the corresponding figure legend that the cartoon is not in scale: “*The schematics are not in scale with the actual size of the corresponding sequences. UTR lengths:* dlg-1 *5’UTR: 61 nucleotides;* sax-7 *5’UTR: 63 nucleotides;* dlg-1 *3’UTR: 815 nucleotides;* unc-54 *3’UTR: 280 nucleotides*.”

○ The GFP as an asterisk illustration may be confusing for some readers. Could you add another rectangular box to depict the gfp coding sequence?

Corrected.

○ This microscopy is beautiful!

Thanks Reviewer #2!

○ Were introns removed? Is the endogenous copy still present?

All the transgenes were analyzed in a wild-type background, therefore, yes, the endogenous copy was still present. All the transgenes possessed introns. We changed the corresponding text as follows: “*To test whether the localization of one of the identified localized mRNAs,* dlg-1*, relied on zip codes, we generated extrachromosomal transgenic lines carrying a* dlg-1 *gene whose sequence was fused to an in-frame GFP and to endogenous or exogenous UTRs*.”. In Fig. 3A “*dlg-1* ORF” has been replaced with “*dlg-1* (ex+int)”.

○ The wording in the legend "CRISPR or transgenic" may be confusing as Cas9 genome editing is still a form of transgenesis.

We added “extrachromosomal” to clarify the nature of the transgene.

○ The authors state that the 5'-3'UTR construct produces perinuclear dlg-1 transcripts but in the absence of DAPI imaging, it's not clear that this is the case.

© 2021. Published by The Company of Biologists under the terms of the Creative Commons Attribution License (https://creativecommons.org/licenses/by/4.0/).

We could not find such a statement, but we tried to clarify the localization of these mRNAs in the text (additionally edited due to the inclusion of image quantification plus statistical analysis): "*The mRNA localization patterns of the two UTR reporters were compared to the localization of* dlg-1 *transcripts from the CRISPR line ("wild-type", Fig. 3A; Heppert* et al.*, 2018). Both reporter strains didn't show a decrease in enrichment at the* CeAJ *and localization dynamics of their transcripts compared to the wild- type cr.dlg-1::gfp (Fig. 3B,C) as it would be expected if dlg-1 UTRs were involved in its localization. These results indicate that the UTR sequences of dlg-1 mRNA are not required for its localization.*"

- Which probe set was used? The gfp probe?

Yes, please see the main text: "*Given that the transgenic constructs were expressed in a wild-type background, smFISH experiments were conducted with probes against the GFP RNA sequence to assess specifically the localization of the transgenic* dlg-1::gfp *mRNAs (*cr.dlg-1::gfp *and* tg.dlg-1::gfp*).*"

- Here, sax-7 is used as a uniform control, but sax-7 is claimed in Fig S1B-D as being perinuclear. This is a bit confusing.

sax-7 mRNA localizes perinuclearly in sporadic instances (Fig S1B), but it is predominantly scattered throughout the cytoplasm (*i.e.*, unlocalized). Perhaps these unlocalized RNAs are not being translated. It presumably localizes perinuclearly in a translation-dependent manner as *sax-7* codes for a transmembrane protein that would be targeted to the ER (*i.e.*, its localization depends on the signal peptide coded in the ORF and not on its UTRs). We have described this ER-type of localization in the introduction and reiterated it partially in the first paragraph of the results. *sax-7* UTRs are therefore presumably not responsible for any subcellular localization, which would instead rely on a signal sequence.

We added sentences to clarify this point:

1. *"We used UTRs from mRNAs that do not localize near cell membranes, namely sax-7 and unc- 54 (Fig. S4A,B)." (main text)*
2. *"Such a localization depends on the signal peptide located in the CDS of sax-7 and not on its UTRs." (Figure legend S4)*

- Figure 4

- Excellent results! Really nice!

Thanks Reviewer #2!

- Fig 4A. The GFP depicted as a circle is strange.

We changed it into a rectangle.

- Fig 4A. Can you include the gene/protein name for easy skimming?

Added.

- Fig 4B. the color here is too faint and it is unclear what is being depicted. Overall, this part of the figure could be improved.

We changed Fig. 4A,B into a more intuitive schematics than the previous one.

o Were the introns removed?

No, the introns were maintained in this and in all our transgenic lines. We described our transgenic lines in the materials and methods section (now with more detail). What we depict in the scheme (Fig. 4A) is the mature RNA (now specified in the figure), therefore no introns depicted. Clarified now also in the main text: *"To determine whether dlg-1 mRNA localization occurs co-translationally,*

© 2021. Published by The Company of Biologists under the terms of the Creative Commons Attribution License (https://creativecommons.org/licenses/by/4.0/).

we designed a transgene to inhibit normal translation by deleting two nucleotides (TG) within the start codon of an otherwise wild-type sequence containing both exons and introns (Fig. 4A,B) and generated two transgenic lines."

- Figure 5

○ Fig 5A. can you add the gene/protein name

Added.

○ Fig 5B. Can you make the example apicobasal (non-apical) mRNA more distinctive? If it had its own peak in the lower trace, the reader would more clearly understand that this mRNA will be excluded from apical measurements whereas it will be included in apicobasal measurements.

We actually wanted to show this specific example: a cytoplasmic mRNA and a junctional mRNA may seem close from the apicobasal analysis (partially overlapping peaks that Reviewer #2 mentioned). With the apical analysis, instead, we can show that these mRNAs are actually not close, and they belong to two different compartments (cytoplasm and junction). We would therefore like to keep the current scheme, while better clarifying this point in the corresponding figure legend.

○ D' - I' The grey font is too light.

Changed.

○ D' - I' The inconsistent y-axis scaling makes it difficult to compare across these samples. Can you set them to the same maximum number?

The values are indeed quite different. We tried to use the same scale, but this would make some of the data unappreciable. The idea was to evaluate, within each graph, how mRNA and protein are localized relative to the junctional marker

○ D' - I' The x-axis labels are formatted incorrectly

Corrected.

○ The practice of masking out the nucleus appears to remove potentially important mRNAs that are not nuclear localized. This could really impact the findings and interpretation. Instead, consider an automated DAPI mask.

We have now fully described how the analyses were performed in Materials and methods (see also Fig. S8 and its corresponding legend). The analyses were fully unbiased.

○ I can't see what the authors are calling membrane diffuse versus cytoplasmic. This is making it hard for me to see their "two step" pathway to localization.

We have re-written the last two paragraphs of the result part and added another main figure (Fig.5) to better clarify your points.

○ Can more details of the quantification be included? How were Z-sections selected, chosen for inclusion? Which Z-sections and how many were selected?

We have now fully described how the analyses were performed in Materials and methods.

○ Also, why do these measurements focus on what I think are the seam cells when Lockwood et al., 2008 show the entire epithelium that is much easier to see?

We are focusing on the seam cells at the bean stage as a model: these are the cells and the embryonic stage where we see the highest localization of *dlg-1* mRNA in the wild-type.

○ Please name these constructs to correlate the text more explicitly to the figures.

© 2021. Published by The Company of Biologists under the terms of the Creative Commons Attribution License (https://creativecommons.org/licenses/by/4.0/).

Added.

○ How many embryos were analyzed for each trace? How many embryos showed consistent patterns?

We have now fully described how the analyses were performed in Materials and methods and in the respective figure legends.

○ Why were these cells used for study here? Lockwood et al., 2008 use a larger field of epithelial cells for visualization.

As stated before: we are focusing on the seam cells at the bean stage as a model: these are the cells and the embryonic stage where we see the highest localization of *dlg-1* mRNA in the wild-type.

- Figure 6

There are major discrepancies between what this figure is depicting graphically and what is described in the text. Again, I'm not comfortable making the "two step" claims this figure purports given the data shared in Figure 5.

We have re-written the last two paragraphs of the result part and added another main figure (Fig.5) to better clarify your points. Schematics and respective quantifications were added next to the images in Fig. 5.

Minor comments - Tables & Supplemental Figures

Table 1

- I think this table could be improved to more clearly illustrate which mRNAs were tested and what their mRNA localization patterns were (for example, gene name identifiers included, etc). Could the information that is depicted by gray shading instead be added as its own column? For example, have a column for "Observed mRNA localization"

We modified Table 1 based on these and the other reviewers' comments.

- Can you add distinct column names for the two columns that are labeled as "protein localization - group"

We modified Table 1 based on these and the other reviewers' comments.

- Can you also add which of these components are part of ASI v. ASII (as described in the introduction?)

We modified Table 1 based on these and the other reviewers' comments.

Supplemental Figure 1
- It is hard to see that some of these spots are perinuclear. More information (membrane marker, 3D rendering, improved metrics) is required to support this claim.

We are not trying to make a big statement out of these data as perinuclear localization for mRNAs coding for transmembrane/secreted proteins is well known. The aim of our study was to describe transcript localized at or in the proximity of the junction. We thought it was worth mentioning these examples of perinuclearly localized mRNAs (*hmr-1*, *sax-7*, and *eat-20* - now also *let-805*) for two reasons: scientific correctness - show accessory results that might be interesting for other scientists - and use as positive controls for our smFISH survey - these mRNAs were expected to have a somewhat perinuclear localization for the reasons mentioned above.

© 2021. Published by The Company of Biologists under the terms of the Creative Commons Attribution License (https://creativecommons.org/licenses/by/4.0/).

- What do these images look like over the entire embryo, not just in the zoomed in section?

We added a column with the zoom-out embryos.

- sax-7 localization in S4 looks similar but a different localization claim is made.

sax-7 mRNA localizes perinuclearly in sporadic instances (Fig S1B), but it is predominantly scattered throughout the cytoplasm (*i.e.*, unlocalized). Perhaps these unlocalized RNAs are not being translated. It presumably localizes perinuclearly in a translation-dependent manner as *sax-7* codes for a transmembrane protein that would be targeted to the ER (*i.e.*, its localization depends on the signal peptide coded in the ORF and not on its UTRs). We have described this ER-type of localization in the introduction and reiterated it partially in the first paragraph of the results. *sax-7* UTRs are therefore presumably not responsible for any subcellular localization, which would instead rely on a signal sequence.

We added sentences to clarify this point:

1. *"We used UTRs from mRNAs that do not localize near cell membranes, namely sax-7 and unc- 54 (Fig. S4A,B)." (main text)*
2. *"Such a localization depends on the signal peptide located in the CDS of sax-7 and not on its UTRs." (Figure legend S4)*

Supplemental Figure 2
- Before adherens junctions even exist dlg-1 go to the membrane - this is really neat!

Thanks Reviewer #2!

Supplemental Figure 3
- Technical question: If either 5 or 3 stack images are used, how does this work? Do they have different z-spacings? Or do they do 5-stack images represent a wider Z-space?

This is the sentence under question: "*Maximum intensity projections of 5 (1.08 µm) (A) and 3 (0.54 µm) (B) Z-stacks*". The space between each Z-stack image is constant in all our imaging and its value is 270 nm. When we consider 5 planes, the distance from the 1st to the 5th is 4 x 270 nm = 1.08 µm, whereas for 3 planes will be 2 x 270 nm = 0.54 µm.

Supplemental Figure 4
- Line #2 retains translation and keeps mRNA localization.

"Line2" retains translation only partially and examples of mRNA localization can be found. Quantification can be now found in Fig. S5B.

- Totally optional, but consider showing both lines in the main figure to illustrate the two possibilities.

We preferred to keep the two lines split in main and supplemental figures to avoid confusion.

- Materials and methods - how did they created the ATG mutations? Is it an array? - why does one translate, and one doesn't?

We clarified this point in Materials and methods: "dlg-1 *deletion constructs ΔATG (SM2664 and SM2663) and ΔL27-PDZs (SM2641) were generated by overlap extension PCR using pML902 as a template.*".
The construct and its co-injection markers used to generate the two lines were the same.

We tried to perform Western blots to identify the length of the poorly translated DLG-1::GFP protein in the second line described in the paper ("line2"), but unfortunately without having it working. Currently, we do not have data to explain the reason(s) why one of the two lines is able to express DLG-1::GFP protein to a very small extent (now quantified in Fig. S5B). Perhaps line 2

© 2021. Published by The Company of Biologists under the terms of the Creative Commons Attribution License (https://creativecommons.org/licenses/by/4.0/).

has more RNA expression, making the probability for some protein production higher. More mRNA could be due to a higher copy number of the transgene or a more active configuration of the array.

Reviewer #3

Major comments

1. The smFISH results are striking and implications exciting. The conclusions made from the smFISH results reported in all Figures will be strengthened considerably by quantifying the mRNA localized to the defined specific subcellular regions. At the very least, localization to the cytoplasm versus the plasma membrane should be determined as performed in Figure 2B, but quantifying finer localization will enhance the conclusions made about regional localization (e.g. CeAJ versus plasma membrane mRNA localization in Figure 5). Inclusion of a non-localizing control in Figures 1-4 will enable statistical comparisons between mRNA localizing and non-localizing groups.

   We added more quantitation, statistics, and negative controls.

2. The script used for smFISH quantitation should be included in the methods or published in an accessible forum (Github, etc). Criteria for mRNA "dot" calling should be defined in the methods. All raw smFISH counts should also be reported.

   We employed now FISH-quant for all our analyses (now added in Materials and methods) and the raw data are provided in an additional supplementary table (Table S1).

3. Figure 2: What is the localizing ratio of a non-localizing control mRNA (e.g. jac-1)? Including an unlocalized control with quantitation would strengthen the localization arguments presented.

   Added.

4. Figure 5: Quantifying colocalization of mRNA and protein (+/- AJM-1) will strengthen the arguments made about mRNA/protein localization.

   We re-adjusted Fig. 5, showing apical views previously shown in Fig. S5. Quantifications of the localized mRNA have been added.

5. Discussion of the CeAJ mRNA localization mechanism is warranted. Do the authors speculate that the newly translated protein drives localization during translation, similar in concept to SRP- mediated localization to the ER, or ribosome association is a trigger to permit a secondary factor to drive mRNA localization, or another model?

   We added a conjecture regarding this topic to the Discussion.

Minor comments

1. Please complete the following sentence: "We identified transcripts enriched at the CeAJ in a stage- and cell type-specific."

Corrected.

2. It would be helpful to provide reference(s) for the protein localization summary in Table 1.

Added.

3. Figure 2B: Did dlg-1 and ajm-1 localize at similar ratios? Appropriate statistics comparing the different ratios may be informative.

© 2021. Published by The Company of Biologists under the terms of the Creative Commons Attribution License (https://creativecommons.org/licenses/by/4.0/).

We modified the graph (also following Reviewer #2's suggestion) and added the requested details.

4. Figure 2: In the paragraph that begins, "Morphogenesis of the digestive track," the text should refer to Figure 2C? If not, the text requires further clarification.

Corrected.

5. Figure 2: Reporting the smFISH localizing ratios of 8E and 16E will be informative.

We added the quantitation for 16E, but we could not add 8E for consistency reasons: we quantified mRNA localization in seam cells which are not established yet at this embryonic stage.

6. Please include citations when summarizing the nonsense-mediated decay NMD mechanism and AJM-1 identifying the CeAJ.

Added.

7. The sentence, "Embryos from our second ΔATG transgenic line displayed a little GFP protein and some dlg-1::gfp mRNA," should refer to Figure S4.

Added.

8. An immunoblot of this reporter versus wild type may be informative regarding the approximate position of putative alternative start codon.

The construct and its co-injection markers used to generate the two lines were the same.

We tried to perform Western blots to identify the length of the poorly translated DLG-1::GFP protein in the second line described in the paper ("line2"), but unfortunately it did not work (perhaps the level of DLG-1 is too low). Currently, we do not have data to explain the reason(s) why one of the two lines is able to express DLG-1::GFP protein to a very small extent (now quantified in Fig. S5B). Perhaps line 2 has more RNA expression, making the probability for some protein production higher. More mRNA could be due to a higher copy number of the transgene or a more active configuration of the array.

9. Figure 5: N's and repetitions performed should be included for localization experiments.

We have included the requested information in the main text, figure legends, and materials and methods. Table S1 also shows the raw data for the quantifications.

10. Please clarify that the "the mechanism of UTR-independent targeting is unknown in any species" refers to dlg-1 mRNA localization.

Added.

11. "Our findings suggest..." discussion paragraph should reference Figure 6.

We simplified our model and removed Fig. 6. Please, find all the changes in the last paragraph of the results and in the discussion.

Second decision letter

MS ID#: DEVELOP/2021/200027

MS TITLE: Translation-dependent mRNA localization to Caenorhabditis elegans adherens junctions

© 2021. Published by The Company of Biologists under the terms of the Creative Commons Attribution License (https://creativecommons.org/licenses/by/4.0/).

AUTHORS: Cristina Tocchini, Michèle Rohner, Laurent Guerard, Poulomi Ray, Stephen E Von Stetina, and Susan E Mango

I have now received all the referees reports on the above manuscript, and have reached a decision. The overall evaluation is positive and we would like to publish a revised manuscript in Development, however, there are some minor concerns that the reviewers point out that would need to be addressed. I do not expect to send the manuscript to the reviewers, but their comments will need to be addressed.

Reviewer 1

Advance summary and potential significance to field

The connection between transcript localization and translation are established but the specific regulatory ques for both localization and expression remain an important question. Here, Tocchini et al. use the C. elegans embryo and single molecule FISH (smFISH) to determine the subcellular localization of key mRNAs involved in epithelial morphogenesis. In particular, analyses of dlg-1/discs large mRNA, an apical junction component, provides evidence that mRNA localization requires active translation, but the 5' and 3' untranslated regions are dispensable. Transcript mapping supports that the L27 N-terminal coding region is necessary to target mRNAs to the apical junctions, but the C-terminal Hook and GuK regions help localize dlg-1 mRNA to the plasma membrane.

The authors were challenged with a monumental re-analysis of their smFISH data and present here a "revision de force." I thought the heat shock experiment was particularly clever and the results support their model. I did have one minor criticism that should be addressed. But overall, the smFISH quantitation was well performed, displayed, and described. Thus, this well written, well-conceived study will be of great interest to those interested in post-transcriptional regulation, mRNA subcellular localization, and animal development. The previous statement holds true that dlg-1 shows promise to be a fantastic model to further investigate the mechanisms underlying translation-dependent mRNA localization.

My expertise again covers post-transcriptional gene regulation, the C. elegans model organism, and fluorescent imaging with smFISH.

Scott Aoki

Comments for the author

MINOR:
1. Figure 3C, Lines 190-191: The authors state that "both reporter strains were enriched at the CeAJ and resembled the type cr.dlg-1::gfp," but the statistics argue that localization is enhanced with the 5' and 3' UTR mutants. Please revise this statement and consider adding a plausible explanation for enhancement.

Reviewer 2

Advance summary and potential significance to field

The revised manuscript presents a clear and nicely done study demonstrating mRNA localization during development of Caenorhabditis elegans embryonic epithelia. Tocchini, et al used smFISH-based method to identify mRNAs associated with the cell membrane or cortex, and with apical junctions. Authors provided an additional and novel insight into the function of one of the identified transcripts – dlg-1/discs large. Using transgenic lines Authors demonstrated that dlg-1 localization to the CeAJ requires active translation. Moreover, Authors mapped protein domains involved in that process and proposed a model in which dlg-1 transcripts are co-translationally localized with the nascent protein. This work will be of significant interest to many workers in the field, and will serve as a reference for future studies of mRNA localization during development of

© 2021. Published by The Company of Biologists under the terms of the Creative Commons Attribution License (https://creativecommons.org/licenses/by/4.0/).

Caenorhabditis elegans. I strongly recommend the manuscript for publication in Development, provided that the Authors consider the following point below.

Comments for the author

The authors responded to all my comments and suggestions from the prior review. I appreciate including FISH-quant analysis and statistics, it markedly improved the interpretation of the data presented in the manuscript.

Minor comment:
Figure 6 – enlarge and unify font size – e.g. it’s impossible to see axis titles from Fig. 6C in the printed version

Reviewer 3

Advance summary and potential significance to field

Tocchini et al. report that mRNA encoding components of the cellular adhesion system localize to membranes. They explore the mechanisms responsible for this location, largely focusing on dlg-1 mRNA as a proxy for the group. They find 1) the 3’UTR of dlg-1 is dispensable for its localization (Figure 3), 2) translation of DLG-1 is required (Figure 4), 3) a C-terminal set of protein domains are required (Figure 4), and 4) that the localization of the mRNA to its final destination occurs in a 2-step process that requires the C-terminal domains to direct mRNA to the membrane and an N-terminal region that further refines their location to the apical junction (Figure 5).

Overall, I found the outlined evidence supported the authors' claims. I found the paper clear, concise, and compelling. The idea of the 2-step localization mechanism is novel and will be of great interest to the local translation community, generally. It also yields some thought-provoking implications for how cellular structures may develop and how those structures, in turn, impact the morphological development of epithelia. The authors went above and beyond to address the reviewer’s previous comments and the paper benefited from their efforts. The images are aesthetically beautiful, the figures are expertly depicted for maximum impact, the supplemental material is thorough, and all quantification is well executed. Indeed, the quantification and statistics add to the manuscript’s rigor and readability.

Comments for the author

Minor questions

- The authors mutated the dlg-1 gene and assessed how different mutations affected mRNA and protein localization but did not comment on any additional phenotypes on epithelial development or the organism's survival. Were any other phenotypes noteworthy?
- Tocchini et al. identified many mRNAs that localize to CeAJ but only dissect dlg-1 for required domains in this paper. Can the authors speculate in the discussion about what domains may be localizing those other transcripts? Are there any interesting regions of conservation between them?

Minor comments

- Line 56 – “… to certain cellular subcellular locations… “ (extra word)
- Line 64 – subject-verb agreement – mechanisms is known
- Figure 1 – missing labels on “G” panel. Inconsistent with the rest of the figure
- Figure 2 – B and D really help to clarify the observations in A and C.

Very nice.

- Line 332 – “Junctional protein in this mutant strain” – just an awkward sentence that could be improved
- Figure 6 – very nice depiction in B & C helped me to quickly read understand the figures D – H
- Line 368 – Start a new paragraph here on “mRNAs coded by orthologs of dlg-1”

© 2021. Published by The Company of Biologists under the terms of the Creative Commons Attribution License (https://creativecommons.org/licenses/by/4.0/).

Second revision

Author response to reviewers' comments

Reviewer 1 Comments for the Author:

MINOR:
1. Figure 3C, Lines 190-191: The authors state that “both reporter strains were enriched at the CeAJ and resembled the type cr.dlg-1::gfp,” but the statistics argue that localization is enhanced with the 5’ and 3’ UTR mutants. Please revise this statement and consider adding a plausible explanation for enhancement.

We added the following sentence: “A slight increase in mRNA localization for the two reporter strains may reflect their different transgenic nature (extra-chromosomal) compared to the wild-type reference (CRISPR).”

Reviewer 2 Comments for the Author:
The authors responded to all my comments and suggestions from the prior review. I appreciate including FISH-quant analysis and statistics, it markedly improved the interpretation of the data presented in the manuscript.

Minor comment:

Figure 6 – enlarge and unify font size – e.g. it’s impossible to see axis titles from Fig. 6C in the printed version

Corrected.

Reviewer 3 Comments for the Author:

Minor questions

•The authors mutated the dlg-1 gene and assessed how different mutations affected mRNA and protein localization but did not comment on any additional phenotypes on epithelial development or the organism's survival. Were any other phenotypes noteworthy?

All our transgenic lines were analyzed in embryos expressing wild-type, endogenous DLG-1, as stated in the main text and materials and methods. No gain-of-function phenotypes were observed in any transgenic line.

•Tocchini et al. identified many mRNAs that localize to CeAJ but only dissect dlg-1 for required domains in this paper. Can the authors speculate in the discussion about what domains may be localizing those other transcripts? Are there any interesting regions of conservation between them?

These aspects will be part of our future research, therefore we would prefer to talk about these topics in our next work.

Minor comments

•Line 56 – “… to certain cellular subcellular locations…” (extra word)
Corrected.

•Line 64 – subject-verb agreement – mechanisms is known

Corrected.

•Figure 1 – missing labels on “G” panel. Inconsistent with the rest of the figure

Corrected.

© 2021. Published by The Company of Biologists under the terms of the Creative Commons Attribution License (https://creativecommons.org/licenses/by/4.0/).

•Figure 2 – B and D really help to clarify the observations in A and C. Very nice.

Thanks Reviewer #3!

•Line 332 – "Junctional protein in this mutant strain" – just an awkward sentence that could be improved

Corrected into: "DLG-1 protein localized at the junction in this mutant strain may reflect…".

•Figure 6 – very nice depiction in B & C helped me to quickly read understand the figures D – H

Thanks Reviewer #3!

•Line 368 – Start a new paragraph here on "mRNAs coded by orthologs of dlg-1"

Corrected.

Third decision letter

MS ID#: DEVELOP/2021/200027

MS TITLE: Translation-dependent mRNA localization to Caenorhabditis elegans adherens junctions

AUTHORS: Cristina Tocchini, Michèle Rohner, Laurent Guerard, Poulomi Ray, Stephen E Von Stetina, and Susan E Mango
ARTICLE TYPE: Research Article

Thank you for sending your manuscript to Development through Review Commons.

I am happy to tell you that your manuscript has been accepted for publication in Development, pending our standard ethics checks.

© 2021. Published by The Company of Biologists under the terms of the Creative Commons Attribution License (https://creativecommons.org/licenses/by/4.0/).
